# Social media promotion improves job market outcomes

**DOI:** 10.1073/pnas.2528289123

**Published:** 2026-05-04

**Authors:** Jingyi Qiu, Yan Chen, Alain Cohn, Alvin E. Roth

**Affiliations:** ^a^ School of Information, University of Michigan, Ann Arbor, MI 48109; ^b^ Department of Economics, Stanford University, Stanford, CA 48109

**Keywords:** field experiment, social media, academic job market

## Abstract

Social media has transformed how academics disseminate research, but its effect on academic job outcomes remains unclear. Previous research has shown correlations between social media exposure and metrics like citation counts, but these relationships may be confounded by unobserved factors such as researcher quality or access to professional networks. We examine whether social media promotion causally affects job market outcomes in economics through a field experiment on Twitter (now X). We first collect tweets about job market papers from 519 candidates and post them from a dedicated account. We then randomize half of the posts to be quote-tweeted by established economists in the candidates’ fields, and measure the effects on both online visibility and hiring outcomes. We find that posts in the treatment group receive 441% more views and 303% more likes than those in the control group. Candidates whose posts were assigned to be quote-tweeted receive one additional flyout invitation compared to the control group average of 5.4 flyouts. Furthermore, women in the treatment group receive 0.9 more job offers than women in the control group, who receive 3 offers on average. Exploring mechanisms, we find that academic reputation drives these results, with stronger effects for quote-tweets from highly cited scholars and for candidates from top institutions. Our findings suggest social media promotion causally increases research visibility and improves academic job market outcomes.

## Significance

Social media has transformed the speed and scope of information dissemination. While academics use these platforms to promote their own and others’ research, their causal impact on hiring remains unclear. Furthermore, persistent disparities hinder women and underrepresented groups in academic hiring. The potential of social media to overcome these barriers is also unclear. Our field experiment on Twitter (now X) demonstrates that social media promotion by established scholars causally increases research visibility and improves job market outcomes. Women, who often self-promote less, received significantly more job offers when their work was promoted by senior scholars in their field. Our findings suggest this low-cost intervention can help mitigate inequities in academic hiring, with implications for understanding how scientific ideas are spread.

Social media platforms have profoundly reshaped how people connect, both personally and professionally. With over 5 billion users worldwide, these platforms reach nearly two-thirds of the global population and 95% of Internet users (1). Users spend an average of 2.4 h daily on social media (2), highlighting their pervasive role in modern life. Beyond connecting people, social media has also become a key source of information for users, with dissemination occurring at unparalleled speed and scope (3). The impact of this information flow extends beyond mere access, as endorsements from influential figures on social media can increase message reach and affect beliefs and behaviors (4). Through these information channels, users become better informed about global events and current affairs (5).

Social media platforms have been especially useful for specialized groups to share information. Among academics, these platforms have become increasingly popular for disseminating their research quickly and widely (6, 7). This trend is particularly evident during the academic job market season in fields such as economics, where candidates—along with their advisors and placement officers—often use platforms like Twitter (now X) to bring their job market papers to a broader audience.^*^

While job candidates routinely use social media to promote themselves during job search, the causal effect of this promotion on job outcomes remains unclear. Prior work shows that social media exposure correlates with academic recognition, such as higher citation counts (8–11). However, this relationship may be confounded by unobserved factors. For example, researchers who actively promote their work online may produce higher-quality papers or have better access to professional networks, making it difficult to disentangle the impact of social media activity from these underlying factors. Social media promotion could even backfire if excessive self-promotion is perceived negatively by hiring committees or if candidates face public criticism on these platforms.

In this paper, we examine whether social media promotion has a causal impact on job market outcomes in the field of economics. We conduct a field experiment on Twitter where we promote candidates’ job market papers through quote-tweets (retweets with comments) from established scholars. In addition to estimating the overall effects, we investigate whether this intervention can help address underrepresentation in academia.

In economics, persistent gender and racial disparities exist throughout the academic pipeline despite efforts by professional associations (12–15). For example, women face higher standards in the peer review process (16), publish fewer papers in leading journals (17), and receive less recognition in coauthored papers (18). Furthermore, women tend to self-promote their work less than men, a gap observed both in general performance evaluations (19) and specifically in scholarly communication on social media. For instance, Peng et al. (7) find that women are 28% less likely to share their own papers on Twitter, with the gap widening among high-performing researchers at top institutions. Racial and ethnic minorities encounter similar challenges within the profession. For example, while minorities comprise 8.3% of economics faculty (20), only 1% of invited talks are given by economists from underrepresented racial and ethnic groups (21). Disparities also extend to social media networks. Ajzenman et al. (22) find that the median number of Twitter followers is 582 for White economists compared to 371 for non-White economists, and 1,224 for those at top-ten institutions compared to 558 for others. Social media promotion by established scholars may therefore help amplify underrepresented candidates who face greater barriers to visibility.

In our experiment, we ask candidates to create tweets about their job market paper, which we share from a dedicated Twitter account. A random subset of these posts are then quote-tweeted by prominent economists working in the same subfield as the respective candidates. The results show that our intervention substantially increases job market paper visibility. Posts assigned to be quote-tweeted receive 441% more views and 303% more likes than those in the control group (which were only posted on the dedicated account without amplification). We also find positive effects on job market outcomes. Candidates in the treatment group receive one more flyout invitation on average than those in the control group, who receive 5.4 flyout invitations. This effect is comparable to having at least one published or revise-and-resubmit (R&R) paper vs. having neither, and roughly half the advantage of graduating from a top 30 institution, which likely reflects multiple benefits including institutional reputation, resources, and advisor networks. We also find a significant positive effect for women, with women in the treatment group receiving 0.9 more job offers than their counterparts in the control group, who receive 3 offers.

Overall, our findings provide causal evidence that social media promotion enhances job market candidates’ online visibility and improves their job market outcomes. These results highlight the potential of social media promotion as a low-cost, scalable tool that may help address disparities for underrepresented groups in academia.

## Experimental Design

Our field experiment was implemented during the 2022–2023 economics job market season. The study was approved by the University of Michigan IRB (HUM00221663) and preregistered at the AEA RCT Registry on November 4, 2022 (23).^†^ Before participating, job market candidates provided informed consent at the beginning of the premarket survey to participate in a study of social media exposure and job market outcomes. The full consent form is reproduced in *SI Appendix*, section 2 (p.11) and states that participants’ job market papers (JMPs) would be promoted on Twitter.

To conduct our experiment, we first constructed a pool of 2,714 job market candidates (JMCs) from the American Economic Association’s (AEA) Job Openings for Economists (JOE), the European Job Market for Economists (EJME), and department websites in September 2022. In October, we created a dedicated Twitter account, Econ Job Market Helper, as the vehicle to post JMP tweets. As academic economists, we then invited our friends and colleagues to follow this account. Throughout the experiment, the account accumulated more than 2,000 followers, placing it well above the 85th percentile of all Twitter users in terms of follower count.

After creating our dedicated Twitter account and building its visibility through the development of a follower base, we reached out to a group of academic economists in late October and early November. We sent invitations to 251 established economists with more than 4,000 Twitter followers,^‡^ of whom 80 participated in our experiment (referred to as “influencers” henceforth).^§^ These influencers had a median of 13,450 Twitter followers (mean: 20,989) and a median of 8,590 Google Scholar citations (mean: 21,223). *SI Appendix*, Fig. S1 plots influencers’ Google Scholar citations against their Twitter follower counts. The figure reveals a weak positive correlation (ρ=0.286) between their academic citations and social media visibility. Given that academic economists tend to follow each other on Twitter, tweets from these influencers have the potential to amplify the reach and impact of shared content within this network. With the participation of these economists secured, we proceeded to conduct our experiment in three phases.

### Phase 1: Premarket Survey.

In early November 2022, we distributed a premarket survey to all 2,714 JMCs in our pool, collecting information on their academic background, job preferences, demographics, and Twitter handles. We also provided them with empirically grounded guidance on what drives attention on social media (24) and asked them to compose a tweet about their JMPs. *SI Appendix*, section 2 contains the premarket survey and response statistics.

We offered two benefits to encourage participation: the opportunity to join a virtual workshop on job market preparation^¶^ and the promise that their JMP tweet would be posted on our dedicated Twitter account. Of the 849 JMCs who completed the premarket survey, 590 also submitted a JMP tweet. We posted these 590 JMP tweets on our Twitter account between November 9 and December 1, 2022. Posting from a single dedicated account ensures that all tweets reach a similar audience, as each candidate’s tweet benefits from the same follower base. Our final sample consists of 519 participants who submitted tweets and had posted their JMPs online before the intervention period.^#^

### Phase 2: Intervention.

Phase 2 consists of assigning participants to either the treatment or control group and implementing our intervention for those in the treatment group.

#### Randomization.

From our pool of 519 JMCs, we categorize participants into one of two groups: Underrepresented Groups (URG, including women, racial and ethnic minorities, and LGBTQ+ individuals) and non-Underrepresented Groups (non-URG).^‖^ We classify JMCs as being at a top 30 institution if they are at a top 30 institution in the United States (*SI Appendix*, Table S41) or a top 30 institution outside the United States (*SI Appendix*, Table S42).^**^ These two classifications create four strata: 1) URG top 30, 2) URG nontop 30, 3) non-URG top 30, and 4) non-URG nontop 30. Within each stratum, we sorted candidates by their main advisor’s Google Scholar citation count and formed triplets of adjacent candidates. For URG strata, we randomly assigned two members of each triplet to treatment and one to control; for non-URG strata, we assigned one to treatment and two to control. The higher treatment probability for URG candidates reflects our aim to provide support to underrepresented candidates. We use this matched-triplet design, an extension of the matched-pair design (25), to increase precision.

#### Matching.

After the randomization, we match the JMCs in the treatment group with influencers in their respective fields. For each JMP, we calculate the cosine similarity between its abstract and the abstracts of each influencer’s top 20 most cited papers on Google Scholar. We match each JMP with the influencer whose work produced the highest cosine similarity score.^††^ Cosine similarity is widely used in natural language processing and text analysis to measure semantic similarity (26, 27). We describe the computation method and provide pseudocode in *SI Appendix*, section 5. After the algorithmic matching process, we manually review the matches and adjust 49 of 247 JMPs to ensure closer alignments in research topics.

#### Intervention.

Between November 28 and December 8, 2022, we emailed each of our 80 influencers, informing them of their assigned JMPs, and asking them to quote-tweet the relevant JMP tweets posted on our dedicated Twitter account, using either a modifiable template we provide or their own wording. Our templates are constructed to be neutral (see *SI Appendix*, Fig. S2 for an example). On average, each influencer is tasked with quote-tweeting three JMP tweets. To ensure a more organic spread of the tweets, we time the emails to avoid a bunching of quote-tweets within a small timeframe. The compliance rate is high, with 88% of assigned tweets quote-tweeted by the influencers.

#### Data.

In January 2023, we collected Twitter metrics including views and likes for the JMP tweets posted on our dedicated Twitter account directly from Twitter Analytics. Following the job market season, in April 2023, we revisited Twitter Analytics to update these metrics for the JMP tweets. We also collected the Twitter API to collect engagement data (such as likes) on the quote-tweets posted by the influencers to examine whether the quote-tweets affect the visibility of JMP tweets relative to the control group.^‡‡^ We measure visibility through the number of views for the JMP tweets (which include views of the quote-tweets). We also examine whether the influencer quote-tweets impact people’s responses to JMP tweets. We measure this type of user engagement through the number of likes received by the JMP tweets and the influencer quote-tweets. Views indicate the reach of the tweets, reflecting how widely the JMP tweets are exposed, while likes reflect endorsements or quality signals of the JMP tweets’ content. We note that 98% of the Twitter activity related to the JMP tweets and influencer quote-tweets occurs in December 2022, around the time of our intervention.

### Phase 3: Postmarket Survey.

We conducted a postmarket survey in May 2023 to collect data on job market outcomes, including the number of interviews, flyouts, and job offers, as well as the salary ranges and level of satisfaction with job placement for those with an offer. The postmarket survey is provided in *SI Appendix*, section 8.

**Fig. 1.**
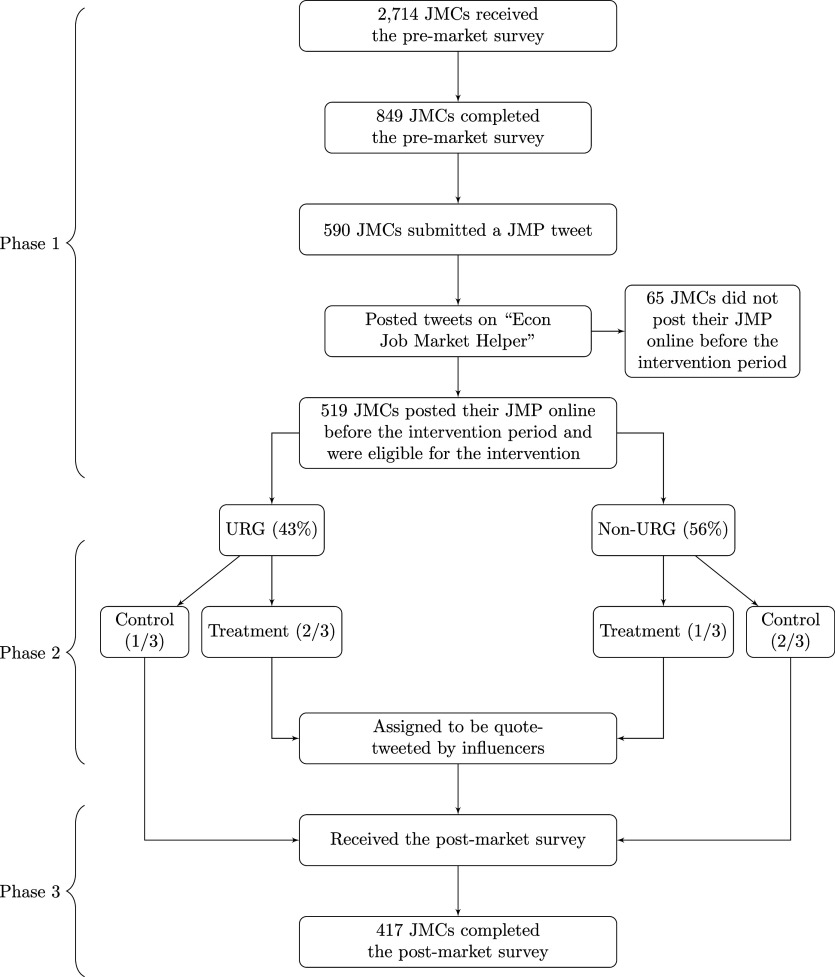
Experiment design.

Fig. 1 summarizes our experimental design and the number of participants in each phase of the experiment. Of the 519 participants who posted their JMP online before the intervention and were thus assigned to either the treatment or control group, 417 (80%) completed the postmarket survey (henceforth, “premarket” and “postmarket” samples).^§§^*SI Appendix*, Table S1 shows that the treatment group was 4 percentage points more likely to complete the postmarket survey compared to the control group, but the difference is not statistically significant (P>0.257). We further find that US citizens and those with a Twitter account were significantly more likely to complete the postmarket survey (P=0.005 and 0.048, column 2). However, we find no evidence of imbalances in observed characteristics between treatment and control groups for either URG or non-URG candidates in both the pre- and postmarket samples (*SI Appendix*, Tables S2 and S3).

Descriptive statistics for the premarket and postmarket samples are presented in *SI Appendix*, Table S4. In the premarket sample, the average participant is 31.6 y old and from a middle class background (mean = 2.2 on a 0 to 4 scale).^¶¶^ Underrepresented groups comprise 43% of the participants, including women (29.7%), Black (2.3%), Hispanic (13.1%), American Indian or Alaskan Native (0.4%), and LGBTQ+ (6.1%) individuals. (Note that there are overlaps between identities, e.g., a participant can be both a woman and Hispanic.) Moreover, 18% are US citizens, and 70% have a Twitter account. These statistics resemble those of the AEA survey of economists on the job market in 2022–2023 (n=623), with a few exceptions (*SI Appendix*, Table S5). (We thank John Cawley for sharing the summary demographics statistics of the AEA survey with us.)

Regarding academic background, 46% of our participants either have obtained or would obtain their PhD degree from a university ranked in the top 30 in the United States or top 30 outside the United States, 87% are on the job market for the first time, 18% have predoctoral research experience, and 15% have postdoctoral experience. 59% of our participants have at least one article published or under revision at the time of entering the job market, compared to 28% in ref. 28’s sample. Most of our participants apply for “101 to 150 jobs,” and 76% use the AEA signaling mechanism to send two signals to express their interest in specific employers (29). (We asked participants to select a range from multiple choice options, as it may be difficult for them to keep track of the specific number of applications submitted.) Two-thirds of participants report the salary of their accepted job in the postmarket survey, and the average pretax annual salary is $107,969 (*SI Appendix*, Fig. S3). Participants were asked to report their pre-tax annual salaries in thousands of US dollars. We identified 148 participants who appear to have reported their salaries in raw dollars instead (indicated by implausibly high values that would exceed $600,000) and thus divided their responses by 1,000.

#### Prediction studies.

To compare actual treatment effects with expert predictions, we conduct two incentivized prediction studies. On December 11, 2023, we distributed the prediction survey through the Social Science Prediction Platform (SSPP) to 73 primarily early-career economists. A few days later, we extended the survey to our 80 influencers and received predictions from 31 of them. *SI Appendix*, section 9 presents the prediction survey and response statistics.

#### Ethical considerations.

After the release of our working paper on the Social Science Research Network (SSRN) on May 20, 2024, a vigorous discussion arose on both social and mainstream media, particularly on Twitter, about the ethics of our experiment and of field experiments more generally (e.g., ref. 30). The main concern suggested that job markets are essentially constant sum, so that randomly promoting some candidates through having their JMPs quote-tweeted by influencers would necessarily (and unethically) disadvantage both those who were in the control condition of the experiment and those who did not participate in the experiment.

We understand the importance of considering the ethical implications of any experiment and that ethicality is connected to the underlying economics of the job market. In this latter respect, given the information friction and congestion in the interview process, job markets are unlikely to be constant sum. Aside from the possibility of welfare gains from improved match quality, we note that, typically in matching markets, many employers fail to fill all their positions while at the same time qualified candidates fail to find one, so that welfare can also be improved by filling more positions. [In the 2022–2023 job market, the total number of jobs listed on JOE was 3,608, including 933 (1,083) full-time academic jobs in (outside) the United States and 718 full-time nonacademic jobs (any location). On the supply side, 1,386 Ph.D. students and postdocs applied to at least one job through JOE from August to December 2022 (31).] In economics, the job market often has unfilled positions by the end of February, leading to a scramble round each year starting in March. Similarly, the annual National Resident Matching Program (NRMP) for new physicians in the United States also leads to some positions being unfilled, despite having far more applicants than available positions. [For example, in 2024, 38,494 positions were offered to 44,853 active applicants and 2,510 positions were unfilled (6.5%), at the end of both the main match (a deferred acceptance algorithm, see ref. 32) and a centralized postmatch scramble called the Supplemental Offer and Acceptance Program (33).]

The phenomenon of unfilled positions in a thick labor market may reflect congestion in the interview process. In such a market, since many positions receive more applications than the number of candidates who can feasibly be interviewed, the matching of interviews to jobs may be imperfect in the sense that an employer can find that none of the people interviewed can be successfully hired, but could have filled the position if more appropriate interviewees had been chosen. To mitigate this issue, signaling mechanisms have been introduced in both the economics and medical markets to facilitate a better matching of interviewees and employers (29, 34). In our context, the quote-tweeting of JMPs may similarly serve to help employers find better matches with their selection of interviewees who can be hired.

We also propose that highlighting suitable candidates from underrepresented groups for a position could potentially expand the overall number of job openings. A notable example is the President’s Postdoctoral Fellowship Program, implemented across multiple institutions including the University of Michigan and the University of California system. This program seeks to recruit future faculty members “with the potential to bring to their research and undergraduate teaching the critical perspective that comes from their nontraditional educational background or understanding of the experiences of groups historically underrepresented in higher education.” (See, e.g., https://presidentspostdoc.umich.edu/, retrieved on August 29, 2025.)

Finally, we consider trends in the broader context of job search in evaluating the ethical considerations related to our study. Social media has become a common channel for academics to advertise the JMPs of their students. Thus, we are not introducing a new channel for candidate promotion, nor are we excluding others outside of our experiment from availing themselves of this channel. Our goal is to understand the extent to which this channel may create visibility or improve outcomes for job candidates, especially since not all candidates may have equal access. Our paper belongs to the class of natural field experiment (35), a class that has seen a growing number of studies in which field experiments are used to assess the effects of market interventions. [A natural field experiment is one “where the subjects do not know that they are in an experiment” (35). In our context, participants were told only that we would arrange for their JMPs to be tweeted, but not that there would be a quote-tweet treatment.] One of the main benefits of conducting a natural field experiment is that it minimizes possible Hawthorne effects (36). These studies are widely accepted and even recognized, with the 2019 Nobel Prize for experiments in development economics. If it is ethical for economists to use experiments to evaluate interventions in other markets, it should also be ethical for economists to study the market for economists. And if it is ethical to promote students who are on the job market, then it should be ethical to study the effects of such promotion.

In sum, from a normative perspective (should scholars promote candidates?), we argue that such promotion can reduce information friction and job market congestion, potentially leading to more efficient matching. From a positive perspective (does promotion matter?), we demonstrate in Results that it increases candidate visibility and improves job market outcomes, especially for women who are traditionally underrepresented in economics.

## Empirical Strategy

To estimate the intent-to-treat (ITT) effects of our intervention, we use ordinary least squares (OLS) and estimate the following regression:

[1] $$\begin{array}{c} Y_{i}=\alpha +\beta_{1}\;\text{Treatment}\;{\text{group}}_{i}+\beta_{2}\;{\text{URG}}_{i}+{\gamma_{1}}^{\prime }X_{i}+{\gamma_{2}}^{\prime }Z_{i}+\epsilon_{i}, \end{array}$$

where $Y_{i}$ is an outcome of interest for individual *i*, Treatment group_*i*_ is an indicator for being assigned to the treatment group, and URG_*i*_ is an indicator for underrepresented groups, which we include to account for the different treatment assignment probabilities between URG ($\frac{2}{3}$) and non-URG ($\frac{1}{3}$) participants. We also control for personal and academic background characteristics, $X_{i}$ and $Z_{i}$, respectively. (For the selection of control variables, we use the double lasso regression method (37). This method identifies variables that predict either the outcome or treatment variable, then takes the union of these two sets as controls. For consistency, we use the same set of control variables for all outcomes, using the variables selected for the number of interviews, which yields the largest set of controls. However, when analyzing intermediate outcomes (Twitter metrics), we use the premarket sample and therefore control only for academic background variables that were collected in the premarket survey.) The parameter $\beta_{1}$ measures the ITT effect. Given the high compliance of our influencers (88%), the local average treatment effects (LATE) are similar to the ITT effects. We therefore focus on the ITT effects in the main text and report the LATE results in *SI Appendix*, section 3.

Our preanalysis plan (*SI Appendix*, section 1) specifies our outcomes as intermediate (Twitter metrics), primary (interviews, flyouts, and job offers), and secondary (satisfaction with job placement). To correct for multiple hypothesis testing, we report the false discovery rate adjusted *q*-values in square brackets (38, 39) for our preregistered intermediate and primary outcomes in Table 1. We follow the convention of using a 5% (10%) cutoff for *P*-values (*q*-values) to claim statistical significance (40).

**Table 1.** Intent-to-treat effects of social media promotion on Twitter visibility, engagement, and job market outcomes

	Views	Likes	Interviews	Flyouts	Job offers
Dependent variable:	(1)	(2)	(3)	(4)	(5)
Treatment group	3,969.650***	11.782***	1.222	1.052**	0.408*
	(524.201)	(1.723)	(1.132)	(0.502)	(0.234)
	[0.001]	[0.001]	[0.115]	[0.039]	[0.066]
URG	463.512	1.255	0.776	0.355	0.067
	(519.259)	(1.844)	(1.138)	(0.536)	(0.235)
Age	−110.429**	−0.470**	−0.268**	−0.125**	−0.100***
	(53.845)	(0.203)	(0.130)	(0.059)	(0.028)
Parental income class	245.115	−0.186	−0.529	0.479	0.128
	(248.966)	(0.923)	(0.699)	(0.324)	(0.148)
US citizen	−788.243*	−2.760*	1.544	0.425	−0.444*
	(429.099)	(1.458)	(1.308)	(0.622)	(0.257)
Twitter account	852.422**	2.463	2.863**	0.621	0.241
	(433.291)	(1.526)	(1.117)	(0.540)	(0.261)
Top 30 PhD institution	130.520	1.933	5.819***	2.239***	0.812***
	(502.443)	(1.698)	(1.133)	(0.498)	(0.231)
First-time JMC	−1,233.218	−2.197	2.512**	1.853***	0.766***
	(870.146)	(1.846)	(1.168)	(0.497)	(0.277)
Predoc	−742.502	−0.928	0.429	0.579	0.253
	(506.940)	(1.797)	(1.318)	(0.622)	(0.341)
Postdoc	−459.400	−2.397	−4.238***	−0.955**	−0.492*
	(617.476)	(1.641)	(1.043)	(0.471)	(0.268)
Publication or R&R (1+)			3.420***	0.973**	0.604**
			(1.123)	(0.490)	(0.246)
# applications (51 to 100)			5.898***	2.139***	1.158***
			(1.484)	(0.648)	(0.317)
# applications (101 to 150)			7.319***	3.223***	1.233***
			(1.416)	(0.682)	(0.337)
# applications (151 to 200)			9.921***	3.015***	1.187***
			(1.788)	(0.738)	(0.395)
# applications (200+)			13.863***	2.815***	1.184***
			(1.685)	(0.663)	(0.326)
AEA signals (2)			2.728**	0.519	0.473*
			(1.148)	(0.511)	(0.253)
Constant	4,460.413**	19.099***	6.969	1.466	2.943**
	(1,914.762)	(6.987)	(5.328)	(2.429)	(1.175)
Observations	500	500	394	394	394
Sample	Premarket	Premarket	Postmarket	Postmarket	Postmarket

Intent-to-treat (ITT) effects of being quote-tweeted by established economists on Twitter. Column 1 shows the effect on JMP tweet views (including views of quote-tweets), and column 2 shows likes received by both JMP tweets and influencer quote-tweets. These Twitter metrics analyses use the premarket sample. Columns 3 to 5 show the effects on interviews, flyouts, and job offers for all job types. All specifications include a dummy for underrepresented group (URG) status to account for the varying treatment probabilities and control for personal characteristics (age, parental income class on a 0 to 4 scale from “In poverty” to “High income/wealthy,” US citizenship, Twitter account) and basic academic background (top 30 PhD institution in/outside the United States, first-time on the job market, pre/postdoctoral experience). Columns 3 to 5 additionally control for whether candidates have at least one publication (or a paper in the revise-and-resubmit stage), application counts, and AEA signals, as these variables may influence job market outcomes but were collected after the Twitter metrics. OLS estimates with robust SEs in parentheses. False discovery rate adjusted q-values are in square brackets to correct for multiple hypothesis testing. Significance levels: *P<0.10, **P<0.05, and ***P<0.01.

## Results

**Fig. 2.**
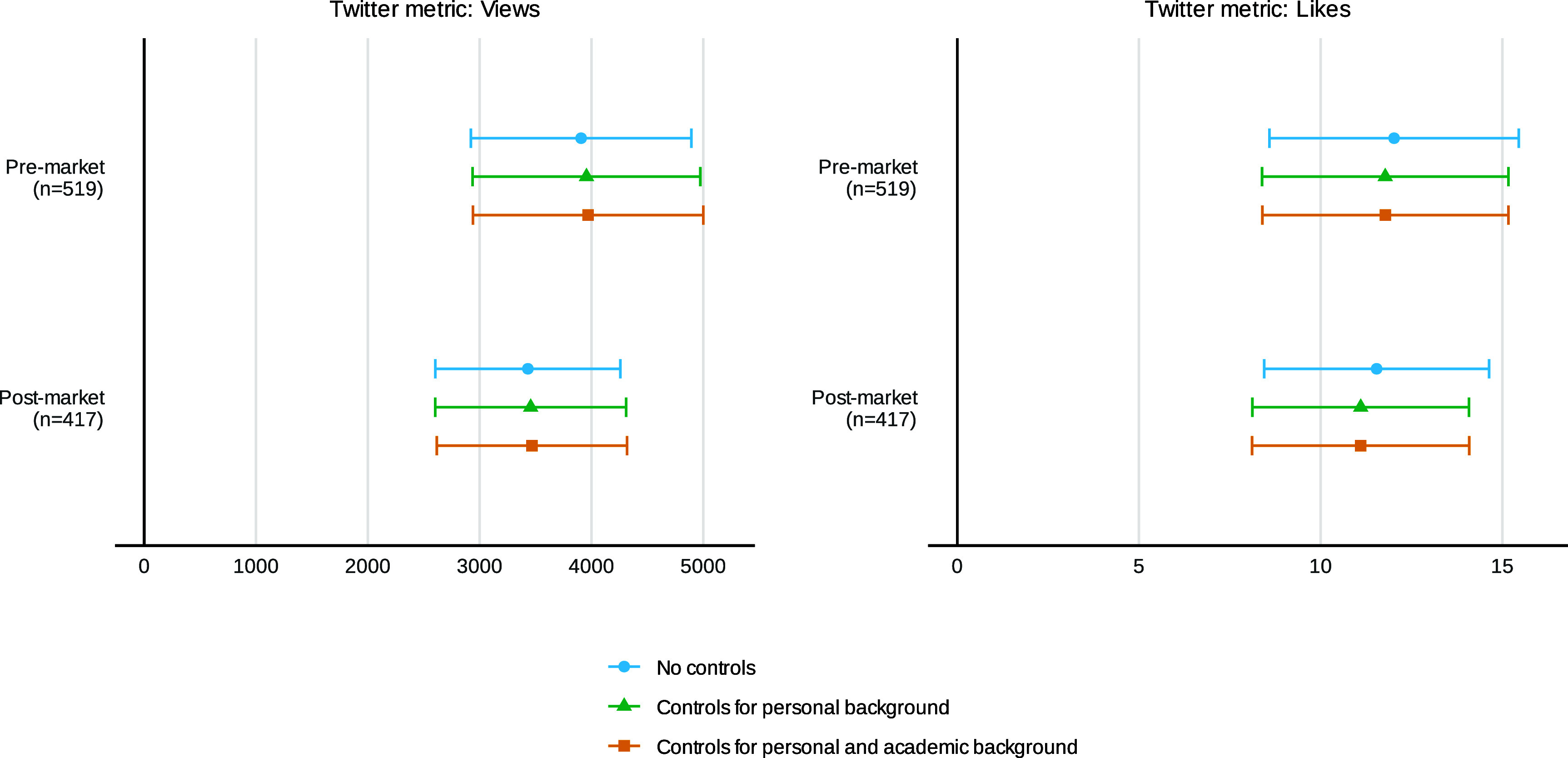
Intent-to-treat effects of social media promotion on Twitter visibility and engagement. OLS estimates with 95% CIs. “Views” represents the number of views received by JMP tweets (including views of quote-tweets), and “Likes” represents the sum of likes received by the JMP tweets and the influencer quote-tweets. All specifications include a dummy variable for underrepresented group (URG) status to account for the varying probability of treatment assignment for URG and non-URG participants. “Personal background” controls for participants’ personal background characteristics such as age, parental income class, US citizenship status, and Twitter account ownership. “Academic background” controls for graduating from a top 30 PhD institution (in or outside the United States), first-time job market presence, and predoctoral and postdoctoral experience. The premarket sample comprises 519 participants, while the postmarket sample includes 417 participants. See *SI Appendix*, Tables S6 and S7 for detailed regression results.

We first examine the impact of our intervention on visibility and engagement on Twitter. The number of views serves as a proxy for the visibility of the JMP tweets, while the number of likes serves as a measure of engagement, interpreted as a signal of endorsement or perceived quality of the paper (or candidate). Fig. 2 summarizes the ITT estimates for the Twitter metrics, with and without controls. To facilitate comparisons between Twitter metrics and subsequent job market outcomes, we present the results for both the premarket sample (all participants) and the postmarket sample (those who reported job market outcomes).

Our results show that JMP tweets from the control group garner 899 views on average. In contrast, tweets from the treatment group receive an additional 3,970 views compared to the control group (P<0.001, q=0.001, 95% CI [2939.7, 4999.6], column 1 in Table 1), representing a 441% increase. Restricting the sample to postmarket survey participants, we find that the treatment group receives 3,468 more views than the control group (P<0.001, 95% CI [2616.7, 4318.6], column 6 in *SI Appendix*, Table S6).

The results for likes follow a similar pattern as those for views. Tweets from the control group receive an average of 3.9 likes. In comparison, tweets from the treatment group receive 11.8 more likes than the control group (P<0.001, q=0.001, 95% CI [8.4, 15.2], column 2 in Table 1), corresponding to a 303% increase. The results are again similar for the postmarket sample (column 6 in *SI Appendix*, Table S6). Tweets by URG participants receive slightly more views and likes than those from non-URG participants, though the differences are not statistically significant (P>0.372, columns 1 and 2 in Table 1). Combined with prior research showing that URG economists have fewer Twitter followers (22) and that women self-promote less on social media (7), this finding suggests that disparities in social media presence may stem from differences in network size and self-promotion behavior rather than from differential engagement with content. Overall, our intervention increases the visibility of and engagement with JMP tweets.

Result 1 (Visibility and Engagement). *JMP tweets of the treatment group received 3,970 more views (+441%) and 11.8 more likes (+303%) on Twitter than those of the control group.*

**Fig. 3.**
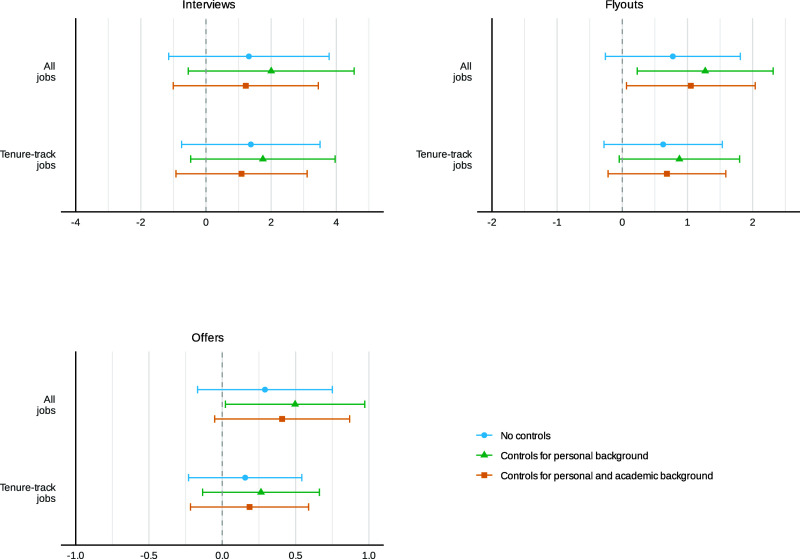
Intent-to-treat effects of social media promotion on job market outcomes. OLS estimates with 95% CIs. “Interviews,” “Flyouts,” and “Offers” represent the additional number of each job market outcome received by the treatment group. All specifications include a dummy variable for underrepresented group (URG) status to account for the varying probability of treatment assignment for URG and non-URG participants. Here, “academic background” controls also include whether participants have at least one publication or paper under revision, the number of applications submitted (in ranges), and whether two AEA signals are sent, in addition to the controls used for the Twitter metrics analysis. “All jobs” refer to all job types, while “Tenure-track jobs” comprise only tenure-track positions. See *SI Appendix*, Tables S10–S12 for detailed regression results.

We next explore whether the increased visibility and engagement on Twitter translates into better job market outcomes. Fig. 3 summarizes the results for all job types and separately for tenure-track positions.

In the field of economics, the job search season begins with securing interviews at the Allied Social Science Associations (ASSA) or the European Economic Association (EEA) meetings held in January and December, respectively. Due to the COVID-19 pandemic, both the AEA and EEA recommended that interviews be conducted online via Zoom for the 2022–2023 job market season. JMCs in the control group secure an average of 16.6 interviews, of which 10.8 are for tenure-track positions. Those in the treatment group secure an additional 1.2 interviews across all job types and 1.1 interviews for tenure-track positions, but the effects are not statistically significant (P=0.281, q=0.115, 95% CI [−1.00, 3.45], column 3 in Table 1; and P=0.287, 95% CI [−0.92, 3.10], column 6 in *SI Appendix*, Table S10). (For clarity, we present the Twitter visibility, engagement, and all job outcome variables with full controls in a single table, Table 1, in the main text. For detailed regression results across different specifications, see *SI Appendix*, Tables S6, S7, and S10–S12.)

Result 2 (Interviews). *The number of interviews received by the participants in the treatment group does not differ significantly from that in the control group.*

The lack of a statistically significant treatment effect at the interview stage could be due to the timing of our intervention. Based on the interview data reported on econjobmarket.org, 199 (2,931) cumulative interview invitations were issued by November 28 (December 8), the start (end) date of our intervention. We also note that interview invitations for the 2022–2023 job market in particular were issued earlier than those for the previous two cycles. (See https://econjobmarket.org/marketState/interviews for the cumulative number of interview invitations issued on EconJobMarket between 2018 and 2025.) Thus, our intervention period, which was based on historical invitation cycles, might have occurred too late in the job process to have an effect on interview invitation decisions.

We next examine the impact of our intervention on flyout invitations. Following the interview phase, the next step in the job market process is to receive invitations to visit university campuses and give job talks, also known as flyouts. In the economics job market, flyouts typically take place between January and March. On average, the control group receives 5.4 flyouts, of which 3.3 are for tenure-track positions. The treatment group receives 1.1 additional flyouts for all job types and 0.7 additional flyouts for tenure-track positions, a 20% increase in both cases (P=0.037, q=0.039, 95% CI [0.06, 2.04], column 4 in Table 1; and P=0.137, 95% CI [−0.22, 1.59], column 6 in *SI Appendix*, Table S12, respectively).

Result 3 (Flyouts). *Participants in the treatment group receive one more flyout than those in the control group, who receive an average of 5.4 flyouts.*

We next examine whether our intervention affects the number of job offers. The control group receives an average of 3 offers, of which 1.5 are for tenure-track positions. The treatment group receives 0.4 more offers for all job types, representing a 14% marginally significant increase (P=0.082, q=0.066, 95% CI [−0.05, 0.87], column 5 in Table 1), and 0.2 more offers for tenure-track positions, which is not statistically significant (P=0.364, 95% CI [−0.22, 0.59] column 6 in *SI Appendix*, Table S12). As noted above, the treatment group is 4 percentage points more likely to complete the postmarket survey (P=0.257, *SI Appendix*, Table S1). To address the possibility that attrition is endogenous to treatment, we estimate Lee bounds for the treatment effects in *SI Appendix*, Table S13. The bounds include zero for interviews and flyouts, consistent with our relatively modest treatment effect estimates in the main analysis.)

Result 4 (Job Offers). *Participants in the treatment group receive 0.4 marginally more job offers than those in the control group, who receive an average of 3 offers.*

In addition to examining the effect of our intervention on job market outcomes, we explore heterogeneous treatment effects with respect to URG by estimating models that include an interaction term between URG and the treatment group indicator. In the control group, we find that URG participants receive slightly more interviews and flyouts but slightly fewer job offers compared to non-URG participants. However, these differences are not statistically significant (P>0.414; columns 3 in *SI Appendix*, Tables S14–S16). We further find that URG participants tend to benefit less from the treatment in terms of flyouts, but more in terms of interviews and job offers. However, these differences are again not statistically significant (P>0.243, columns 3 in *SI Appendix*, Tables S14–S16).

In addition to examining the effects for underrepresented groups, we explore differences in our treatment effects by gender, as women account for 71% of the URG participants in our sample. While we do not find heterogeneous treatment effects for the URG group as a whole, we do find that women in the treatment group receive 0.9 more job offers than women in the control group (P=0.024, column 3 of *SI Appendix*, Table S19), who receive an average of 3 job offers. In contrast, nonfemale participants in the treatment group do not receive significantly more offers than their counterparts in the control group (P=0.615, column 3). (The nonfemale group mainly consists of male participants, but also includes some participants who did not disclose their gender, or participants who are nonbinary.) Overall, the positive ITT effect for women on job offers suggests that our intervention has the potential to help improve women’s job prospects in the economics job market.

Result 5 (Gender). *Women in the treatment group receive 0.9 more job offers compared to women in the control group, who receive an average of 3 offers.*

While the ITT estimates represent a conservative measure of the treatment effects by considering all participants regardless of whether their JMP tweet has been quote-tweeted, we next examine the results using our LATE analysis. To derive our LATE estimates, we employ two-stage least-squares (2SLS) regressions, using the treatment assignment as an instrument for the actual treatment received.

Our LATE results suggest that, overall, treated JMCs experience improved job market outcomes, although not all improvements are statistically significant. Specifically, treated JMCs receive 1.4 more interviews, a nonsignificant 8% increase compared to the control group (P=0.270, column 3 in *SI Appendix*, Table S20). However, we also find that they secure 1.2 more flyouts, a significant 22% increase (P=0.033, column 3 in *SI Appendix*, Table S21). Treated JMCs further receive 0.5 more job offers, a 15% increase, although this effect is only marginally significant (P=0.075, column 3 in *SI Appendix*, Table S22).

We are interested in how the actual results compare to the predictions made by our influencers and SSPP experts, who were asked to guess the treatment effects (LATE) as percentage increases relative to the control group.

**Fig. 4.**
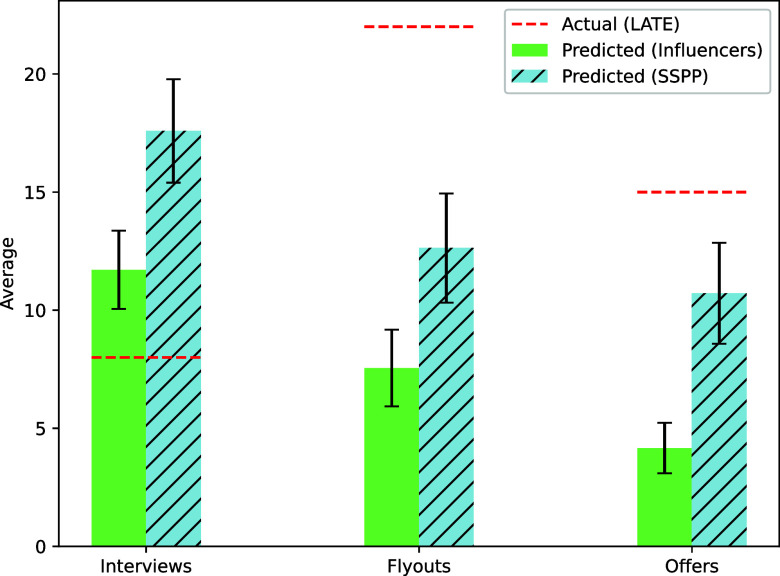
Predictions by economist influencers and SSPP experts regarding the effects of social media promotion on the percentage increase in interviews, flyouts, and job offers. Bars show each group’s mean forecast, with error bars representing the s.e.m. Dashed lines show the LATE estimates from models with personal and academic background controls (columns 3 of *SI Appendix*, Tables S20–S22), expressed as percent changes by dividing the estimated treatment effects by the raw control group means from the same sample.

Fig. 4 presents the predictions of each group compared to the actual results (red dotted line). While both influencers and SSPP experts predict a monotonic decline in effect size from interviews to job offers, our influencers consistently provide more conservative estimates compared to SSPP experts. Interestingly, both groups overestimate the positive impact of quote-tweeting on interviews and underestimate the positive impact on flyouts and job offers. As discussed above, our smaller observed effect on interviews could be due to the timing of our intervention.

We next examine the treatment effects on salaries for accepted jobs (This outcome was not preregistered.) and job placement satisfaction, but fail to find significant treatment effects in either outcomes (see *SI Appendix*, Table S23 for salaries and *SI Appendix*, Fig. S4 for job placement satisfaction).

Turning to job preferences and aggregate outcomes, our premarket survey indicates that 81% of our JMCs strongly prefer tenure-track jobs (rating of 7 on a 1 to 7 Likert scale). Among those who complete the postmarket survey, 92% in both the treatment and control groups have secured a job at the time of the postmarket survey.

To better understand what drives the effects on interviews, flyouts, and job offers, we examine three potential mechanisms: increased visibility, perceived endorsement, and boosted candidate confidence. Our analysis suggests that academic reputation serves as the primary mechanism rather than simple attention or explicit endorsement (see *SI Appendix*, section 6 for more details).

While our intervention substantially increases tweet visibility, candidates assigned to low-reach influencers (below-median followers) achieve better outcomes, securing 3.6 more interviews (P=0.012) and 1.4 more job market outcomes flyouts for tenure-track jobs (P=0.024) than those assigned to high-reach influencers. Since low-reach influencers tweet less frequently (2.3 vs. 6.4 tweets per day, P=0.002), their more selective posting may attract focused attention from relevant academic audiences.

Endorsement effects operate primarily through academic reputation rather than enthusiastic language. Candidates assigned to highly cited scholars (above-median Google Scholar citations) receive 2.1 more flyouts (P=0.002) than those assigned to less-cited influencers. Similarly, candidates from top 30 institutions receive 4.3 more interviews (P=0.023) and 2.1 more flyouts (P=0.004) from the intervention. Yet tweet language, as evaluated by human coders and large language models, shows limited effects—most quote-tweets are relatively neutral in tone. These patterns suggest that for academic social media, who shares the work matters more than how enthusiastically they share it.

We find limited evidence that increased confidence drives our results. Since applications were submitted before our intervention, confidence can only affect interview and job talk performance, not initial screening. While we observe relatively larger effects on later-stage outcomes, this pattern may reflect the timing of our intervention. Many employers had already finalized interview invitations when the intervention began. To more directly test for confidence effects, we examine whether treated candidates increased their tweeting activity after the intervention. Our difference-in-differences analysis reveals no significant increase in tweet volume by treated candidates, suggesting confidence is unlikely to explain our findings.

## Discussion

Our study examines whether the promotion of job market papers by established economists on Twitter increases the visibility of these papers and improves candidates’ job market outcomes. The results show that social media promotion significantly increases the visibility and engagement of these papers, leading to 441% more views and 303% more likes than papers in the control condition.

Regarding job market outcomes, we find that participants in the treatment group receive one additional flyout compared to those in the control group, who receive an average of 5.4 flyouts. Moreover, women in the treatment group obtain 0.9 more job offers than their counterparts in the control group, who average 3 job offers. These results indicate that social media promotion can be an effective and scalable tool for improving job market outcomes, especially for women who have historically been underrepresented in economics.

One potential way to scale up our results is for an institution with convening power, such as the American Economic Association, to set up a job market paper X or Bluesky account, similar to the Econ Job Market Helper account in our experiment. Each year, job market candidates could be invited to submit a tweet about their job market paper to this account. The AEA could match each job market paper with an economist influencer and encourage the influencers to quote-tweet JMPs in their field. If the official account became widely followed, it could help level the social media playing field, much as the AEA signaling mechanism helped level the playing field for students who lack well-connected advisors (29). Our results suggest that such promotion could particularly benefit women, who face greater barriers to visibility.

Finally, our conclusions should be considered in light of certain limitations. First, our sample size was limited by the number of candidates willing to participate in our study during an intense period of job search, limiting statistical power. Second, since our data were collected within one year of the 2022–2023 job market, we cannot assess the long-term career impacts of our intervention. Other studies suggest that social media promotion can have extended effects. For example, a recent Twitter experiment in biological sciences finds that research papers promoted by science communicators receive 12% more citations over three years, but sample size constraints similarly led to statistically insignificant results (41). (A related observational study on Twitter finds that articles endorsed by influencers in the field of AI and machine learning have median citation counts 2 to 3 times higher than those of the control group over a five-year period (11).) In economics, an observational study finds that papers with VoxEU columns receive 16 to 25% more citations over nine years when their columns receive at least one tweet (10). (VoxEU is an online platform featuring economic papers and research.) While these studies suggest that there are sustained benefits from online visibility, future research should examine the long-term effects of social media promotion on academic careers, especially for scholars from underrepresented groups.

## Supplementary Material

Appendix 01 (PDF)

## Data Availability

Replication materials have been deposited in openICPSR (42). The public package contains the main analysis dataset with synthetic Twitter engagement variables and original job market outcome variables, the prediction study dataset, codebooks, and code. It allows replication of Table 1 columns 3-5, Fig. 3, and Fig. 4. *Supplementary appendix* analyses that require additional identifying variables are not included in the deposit to protect participant privacy and reduce re-identification risk. Dissemination of the deposited files has been delayed until April 9, 2035.

## Footnotes

^*^Twitter was acquired by Elon Musk on October 27, 2022, and was rebranded X in July 2023.

^†^Our preregistration at the AEA RCT Registry is accessible at https://doi.org/10.1257/rct.10341-1.0, and included in *SI Appendix*, shortened as *SI Appendix*, section 1.

^‡^Note that our initial preregistration specified influencers with more than 5,000 followers (*SI Appendix*, section 1). In practice we lowered this threshold to 4,000 to ensure sufficient coverage across multiple fields, since some fields had relatively few influencers with more than 5,000 followers.

^§^We note that both the influencers in our study as well as economists who were invited to participate in the study may also be on hiring committees or have students entering the job market (see *SI Appendix*, section 2 for the email invitation template). Since these individuals were apprised of our experimental design, we note that their subsequent hiring-related decisions may or may not have been informed by this knowledge.

^¶^We thank Frank Schilbach for suggesting this idea. We also thank Ellen Muir, Kathleen Ngangoue, and Frank Schilbach for generously taking the time to serve as panelists at the virtual workshop, which took place on November 19, 2022.

^#^We exclude three participants who completed the premarket survey twice using different email addresses and ended up being assigned to both conditions. Note that our final sample size falls short of our preregistered target of 800 participants (400 per condition) needed to detect a minimum effect size of 0.2 SDs (δ=0.2σ) with 80% power and a significance level of 5%.

^‖^Our definition of racial and ethnic minorities is consistent with the AEA Committee on the Status of Minority Groups in the Economics Profession (see https://www.aeaweb.org/about-aea/committees/csmgep/market). Non-URGs in our sample include Asian and white men who identify as heterosexual.

^**^We rely on the 2022 ranking of Economics Departments from *US News and World Report* for the United States and the 2022 RePEc Top 100 Economics Department ranking for institutions outside the United States. See *SI Appendix*, section 4 for details.

^††^We used abstracts to reduce computation time, possibly resulting in a noisy measure of the true match quality.

^‡‡^Note that Twitter Analytics is only accessible by the account owner, whereas Twitter API can be used to access other Twitter accounts.

^§§^This includes five participants who emailed us their job market outcomes instead of completing the postmarket survey.

^¶¶^We coded parental income class as follows: 4 for “High income/wealthy,” 3 for “Upper middle class,” 2 for “Middle class,” 1 for “Low income,” and 0 for “In poverty.”
