# Supplementary material for: Social media promotion improves job market outcomes
Source: Proc Natl Acad Sci U S A. 2026 May 4;123(19):e2528289123. doi: 10.1073/pnas.2528289123 (PMC13167757; doi:10.1073/pnas.2528289123)
Supplement: Supplementary file 1 — Appendix 01 (PDF) [file pnas.2528289123.sapp.pdf]

Supporting Information for

Social Media Promotion Improves Job Market Outcomes

Jingyi Qiu, Yan Chen, Alain Cohn, and Alvin E. Roth

Alvin E. Roth and Jingyi Qiu.
E-mail: alroth@stanford.edu, jaqiu@umich.edu

This PDF file includes:

Supporting text
Figs. S1 to S4
Tables S1 to S42
SI References

Supporting Information Text

1. Pre-analysis Plan

Our pre-registration went through four iterations at the AEA RCT Registry. We consider Version 1.0, published on November 4, 2022, before our intervention started, as the original one.

- **Version 1.0** (November 4, 2022): Specified target recruitment of 400 participants each for treatment and control groups. Despite our best efforts, we did not meet these target numbers. Accessible at https://doi.org/10.1257/rct.10341-1.0

- **Version 1.1** (May 8, 2023): Made several adjustments including: adjusted planned sample size from 800 to 525 due to recruitment constraints and refined power calculations (Minimum Detectable Effect 0.25 instead of 0.2); specified primary outcomes more precisely as the number of interviews, flyouts, and job offers, with secondary outcomes clarified as satisfaction with job placement and Twitter metrics; added specific post-market survey questions to collect information on job applications, signals, scramble participation, early offers, and self-promotion on Twitter; introduced a prediction study with influencers; expanded JMC recruitment beyond AEA JOE to include EJME and departmental listings; implemented a matched-triplet randomization design; adjusted treatment assignment probabilities from 80% to two-thirds for URG candidates; and disaggregated outcomes by job type. Accessible at https://doi.org/10.1257/rct.10341-1.1

- **Version 1.2** (May 11, 2023): Modified empirical strategy by removing matched-triplet dummy controls. Accessible at https://doi.org/10.1257/rct.10341-1.2

- **Version 1.3** (May 21, 2025): Updated trial status to "completed" and marked the analysis plan as public. Accessible at https://www.socialscienceregistry.org/trials/10341

Below we reproduce the original pre-analysis plan as pre-registered.

1.1. Introduction. We are planning to conduct a field experiment to investigate the effects of social media exposure on job market outcomes. Although our sample will include any kinds of job market candidates, our focus is on individuals from under-represented groups (i.e., women, URM and LGBTQ+). For our study, we will recruit job market candidates who register on the AEA's job openings for economists (JOE) during the 2022-2023 economics job market season (due to sample size requirements, we will likely run the experiment also in the subsequent year(s)). We will first send out a pre-market survey to collect candidates' demographics, job market paper summary, and the use of Twitter. Then we will tweet the job market papers of all survey respondents on our Twitter account "Econ Job Market Helper" (@*econ_jmp*). After stratified random assignment, we will match job market candidates in the treatment group with academic economists with 5,000 or more Twitter followers ("influencers") by fields. Influencers will retweet job market papers from the treatment group using templates provided by us and then we will measure whether the retweets improve the job market outcomes of treated participants (i.e., more job interviews, flyouts, job offers etc.).

We are focusing on job market candidates from underrepresented groups (URG) because they typically do not have access to the same high-quality mentoring resources and professional networks as other candidates (1, 2). Amplifying the visibility of URG job market candidates may provide a low-cost alternative to help young economists from underrepresented groups succeed in the job market.

1.2. Research Strategy. Our experiment consists of three stages: (i) a pre-market survey, (ii) an intervention period, and (iii) a post-market survey.

Pre-market survey (mid-late October, 2022): We will compile a list of job market candidates who register on the AEA JOE during the 2022-2023 economics job market. Then, we will invite them to participate in the pre-market survey. The survey will ask job market candidates questions about demographics, job market paper, and use of Twitter. We will also ask participants to summarize their job market paper as a tweet.

Intervention period (late October to mid November, 2022): We will use our Twitter account "Econ Job Market Helper" (@*econ_jmp*) and post the job market paper tweets of all job market candidates who fill out the pre-market survey. After stratifying by URG/non-URG as well as department ranking, we will randomly assign participants to the treatment or control group. Since we are mainly interested in the outcomes of URG job market candidates, they will have a higher probability (80%) to be assigned to the treatment group. Those assigned to the treatment group will be matched with an influencer in their respective field. The influencers will then retweet the original tweet according to a predefined schedule. The texts in the retweets will be randomly drawn from a list of templates. For example: "Retweeting a JMP from @*econ_jmp* to help publicize job market candidates in my field." Influencers can edit the sentence if they want to.

Post-market survey (April-June, 2023): The survey will collect job market outcomes, such as the number of interviews, fly-outs and offers received from PhD/non-PhD-granting departments, as well as salary and additional compensation/perks. In addition, between November 2022 and June 2023, we will collect the number of followers of job market candidates via Twitter API, so that we can measure the number of new followers added during the job market season. We will also collect other intermediate outcomes generated by job market paper tweets via Twitter API and Twitter Analytics, including the number of url clicks of candidates' personal websites, the number of replies, likes, retweets, impressions, engagements, detail expands, user profile clicks, new follows etc.

1.3. Outcomes and Experimental Design. Primary outcome measures

Source: post-market survey

- Number of ASSA job interviews (by PhD/non-PhD-granting departments)
- Number of fly-outs (by PhD/non-PhD-granting departments)
- Number of job offers (by PhD/non-PhD-granting departments)

Secondary/Intermediate outcome measures

- Salary + additional compensation/perks (by academia/public sectors/industry) Source: post-market survey
- Twitter influence index (composite measure, such as number of new Twitter followers)

Long-term outcomes (not included in this paper)

- Number of citations of the job market paper in 2 years (longer-term effects)
- Number of co-authors in 2 years (longer-term effects)
- Publications (how many, journal ranks) in 2 years (longer-term effects)

Control and auxiliary variables

Data sources are marked after "←"

- Appears on the board Minority Job Market Candidates 2022-23 (binary) ← scrape this data
- # Google scholar citations of reference letter writers ← by parsing job market candidates' CV
- # job applications (by types, if post-market survey is able to ask applications by types) ← post-market survey
- Publication - how many, journal ranking (based on RePEc ranking) ← by parsing job market candidates' CV
- Research grants - how many grants and the amount of money if applicable ← by parsing job market candidates' CV
- Number of conference/seminar presentations ← by parsing job market candidates' CV Referee reports - how many, journal ranking (based on RePEc ranking) ← by parsing job market candidates' CV
- Department ranking ← by parsing job market candidates' CV
- Field of specialty ← pre-market survey
- Types of jobs JMC is looking for ← pre-market survey
- Countries/Regions of jobs JMC is looking for ← pre-market survey
- Job Market Signaling (dummy variable, send one signal/two signals/not send signal) ← post-market survey
- Whether JMC participates in Job Market Scramble (binary variable) ← post-market survey
- Impact by COVID (seven relevant questions, five-degree likert scale from strongly agree to strongly disagree) ← post market survey
- Spouse/partner: whether have a spouse/partner, whether in academia, highest level of education, on the market at the same year, whether spouse's preference affects what year entering the market, # applications, # interviews choosing to accept, # fly-outs choosing to accept, which job to accept ← post-market survey
- Whether attended the navigating JM interview workshop ← screenshot of zoom participants
- Mentoring programs (multiple mentoring programs, dummy variables) ← pre-market survey
- Number of followers influencers have ← Twitter public API
- Whether the influencer tweets their students (dummy variable, 1 if a JMC is tweeted by an influencer who tweets their students, 0 otherwise) ← Twitter public API
- Whether the JMC is tweeted by their own advisor(s) ← Twitter public API
- Number of tweets influencers tweet/retweet during the intervention month ← Twitter public API
- Quality of JMC tweet ← recruit ante-post raters

1.4. Pre-market Survey.

1.4.1. Academic background.

1. Are you on the 2022-2023 job market?
   - Yes
   - No

   →If "No" is selected, skip to the end of the survey
2. Is this the first time you are on the Economics Job Market?
   - Yes
   - No
3. If no: Which year did you go on the Economics Job Market the first time?
4. What is the name of the institution at which you (are expected to) finish your PhD?
5. Have you ever been a full-time research assistant (also known as "pre-doc") before entering your PhD program?
   - Yes
   - No
6. If yes: what is/are the name(s) of your pre-doc advisor(s)?
   - Advisor 1's name:
   - Advisor 2's name:
   - Advisor 3's name:
   - Advisor 4's name:
7. Have you done (are you doing) a postdoc?
   - Yes
   - No

1.4.2. Field of specialty.

1. Please indicate your research fields (You can identify at most three research fields).
   - Behavioral economics
   - Development economics
   - Econometrics
   - Economic history
   - Economics of education
   - Environmental/Agricultural economics
   - Financial economics
   - Health economics
   - Industrial organization
   - Information economics
   - International economics
   - Labor economics
   - Law and economics
   - Macroeconomics
   - Microeconomics
   - Political economy
   - Public economics
   - Urban economics
   - Other, please specify
2. What are the names of your primary advisors?
   - Advisor 1's name:
   - Advisor 2's name:
   - Advisor 3's name:
   - Advisor 4's name:
   - Advisor 5's name:
3. Please upload the latest version of your CV:
4. Do you have a personal webpage?
   - Yes
   - No
5. If yes, what is the URL of your personal webpage?

1.4.3. Support from advisors.

1. How satisfied are you with the support you received from your dissertation advisor(s) during your PhD? (1-7)
   Rating: Not at all satisfied (1) - Very satisfied (7)

1.4.4. The types & locations of jobs you are looking for.

1. Please describe what types of jobs you are interested in / applying to:
   Rating: Not at all interested (1) - Very interested (7)
   - Assistant professor (tenure-track) at a college or university
   - Lecturer (non-tenure track) at college or university
   - Postdoctoral fellowship at college or university
   - Government, including central banks
   - Non-profit or quasi-governmental organizations (IMF, World Bank)
   - Contract research organizations / think tanks (e.g., RAND, Mathematica, Research Triangle Institute)
   - Private sector (e.g., banking, finance, tech, consulting, or other business/ industry)
2. In which geographic regions are you applying for jobs? You can select multiple choices.
   - U.S.
   - Other North America
   - Africa
   - Asia
   - Oceania/Australia/New Zealand
   - South America
   - Europe
   - Other, please specify

1.4.5. Twitter account.

1. Do you have a Twitter account?
   - Yes
   - No
2. If yes: What is your Twitter handle?
   Your Twitter handle (e.g., @abc):

1.4.6. Demographics.

1. What is your gender?
   - Woman
   - Man
   - Non-Binary
   - Prefer not to say
2. Do you consider yourself to be transgender?
   - Yes, I am transgender
   - No, I am cisgender
   - I do not consider myself either transgender or cisgender
   - Don't know / Not sure
   - Prefer not to say
3. Do you consider yourself to be...
   - Straight
   - Gay
   - Lesbian
   - Bisexual
   - Pansexual
   - Queer
   - Asexual
   - Prefer not to say
4. In what year were you born?

- I was born in... YYYY
- Prefer not to say

5. What is your country of citizenship? (If you have dual citizenship then please indicate your primary citizenship.)
   - - Select Country - -
6. Are you of Hispanic, Latino, or Spanish origin?
   - Yes
   - No
   - Prefer not to say
7. What is the primary ethnicity or race you identify with?
   - White
   - Black or African American
   - American Indian or Alaska Native
   - Asian
   - Native Hawaiian or Other Pacific Islander
   - Other, please specify
   - Prefer not to say
8. What was the highest level of education achieved by your mother?
   - Less than high school
   - High school diploma or equivalent
   - Associate's degree
   - Bachelor's degree
   - Master's degree
   - Professional degree (e.g. MBA, J.D., M.D.)
   - Doctoral degree (e.g., Ph.D., Ed.D.)
   - Don't know
   - Prefer not to say
9. How would you describe the financial situation of your family when you grew up?
   - High income / wealthy
   - Upper middle class
   - Middle class
   - Low income
   - In poverty
   - Prefer not to say

1.4.7. Instruction to write a tweet. As a thank you for participation, we will post a tweet about your job market paper (JMP) on our Twitter account "Econ Job Market Helper" (@*econ_jmp*). This account is already followed by some influential economists.

Because you know your JMP best, we are asking you to prepare the text that we will use verbatim for the tweet. It should describe the main idea and/or result of your job market paper. Note, the text limit is 280 characters. You can also use emojis (each emoji counts as two characters). Below are some guiding questions:

- What is the research question? Try to frame it in a broader context so that the tweet will create more interest.
- What data/method/modeling approach do you use and what is your main finding? Try to state your data/method/modeling approach and finding(s) in a precise way.
- Add a link to your personal website and, if you have a Twitter account, your Twitter handle (e.g., @abc).

Here are some additional tips to improve the quality of your tweet:
Do's:

1. Use numbers if applicable
2. Use open ended questions

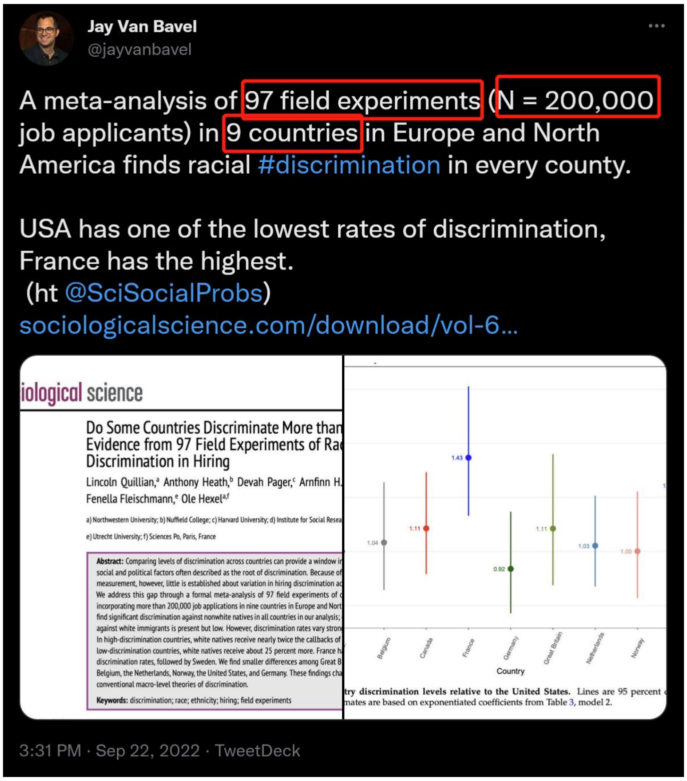
Jay Van Bavel
@jayvanbavel
A meta-analysis of 97 field experiments (N = 200,000 job applicants) in 9 countries in Europe and North America finds racial #discrimination in every county.
USA has one of the lowest rates of discrimination, France has the highest.
(ht @SciSocialProbs)
sociologicalscience.com/download/vol-6...
iological science
Do Some Countries Discriminate More than
Evidence from 97 Field Experiments of Ra
Discrimination in Hiring
Country
3:31 PM · Sep 22, 2022 · TweetDeck

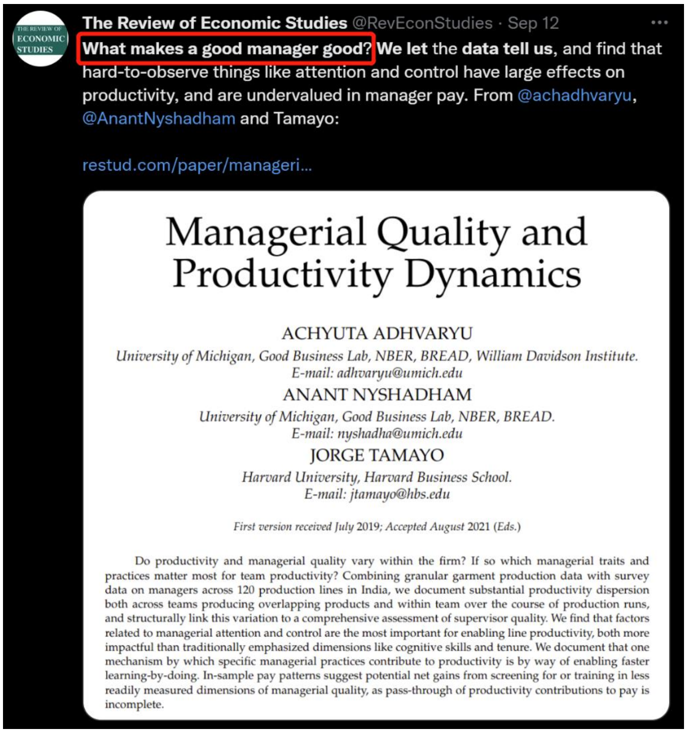

The Review of Economic Studies @RevEconStudies · Sep 12

What makes a good manager good? **We let** the **data tell us**, and find that hard-to-observe things like attention and control have large effects on productivity, and are undervalued in manager pay. From @achadhvaryu, @AnantNyshadham and Tamayo:

restud.com/paper/manageri...

Managerial Quality and Productivity Dynamics

ACHYUTA ADHVARYU

University of Michigan, Good Business Lab, NBER, BREAD, William Davidson Institute.
E-mail: adhvaryu@umich.edu

ANANT NYSHADHAM

University of Michigan, Good Business Lab, NBER, BREAD.
E-mail: nyshadha@umich.edu

JORGE TAMAYO

Harvard University, Harvard Business School.
E-mail: jtamayo@hbs.edu

First version received July 2019; Accepted August 2021 (Eds.)

Do productivity and managerial quality vary within the firm? If so which managerial traits and practices matter most for team productivity? Combining granular garment production data with survey data on managers across 120 production lines in India, we document substantial productivity dispersion both across teams producing overlapping products and within team over the course of production runs, and structurally link this variation to a comprehensive assessment of supervisor quality. We find that factors related to managerial attention and control are the most important for enabling line productivity, both more impactful than traditionally emphasized dimensions like cognitive skills and tenure. We document that one mechanism by which specific managerial practices contribute to productivity is by way of enabling faster learning-by-doing. In-sample pay patterns suggest potential net gains from screening for or training in less readily measured dimensions of managerial quality, as pass-through of productivity contributions to pay is incomplete.

3. Use emojis

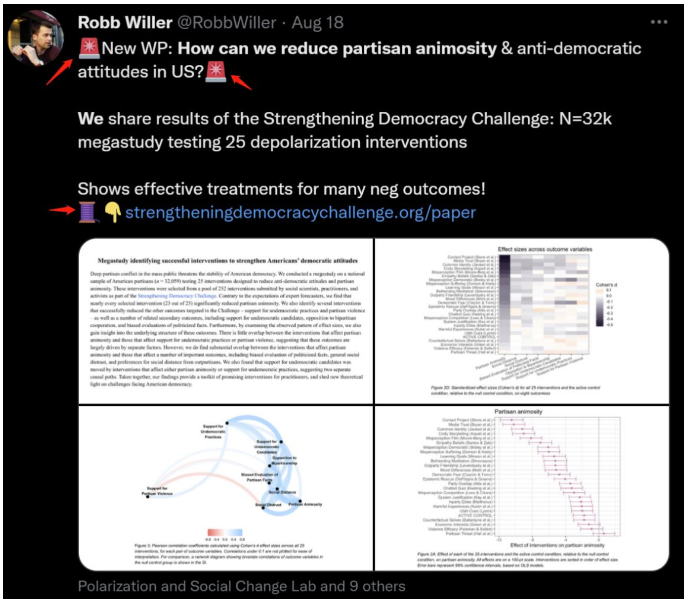

4. Focus on first and last words

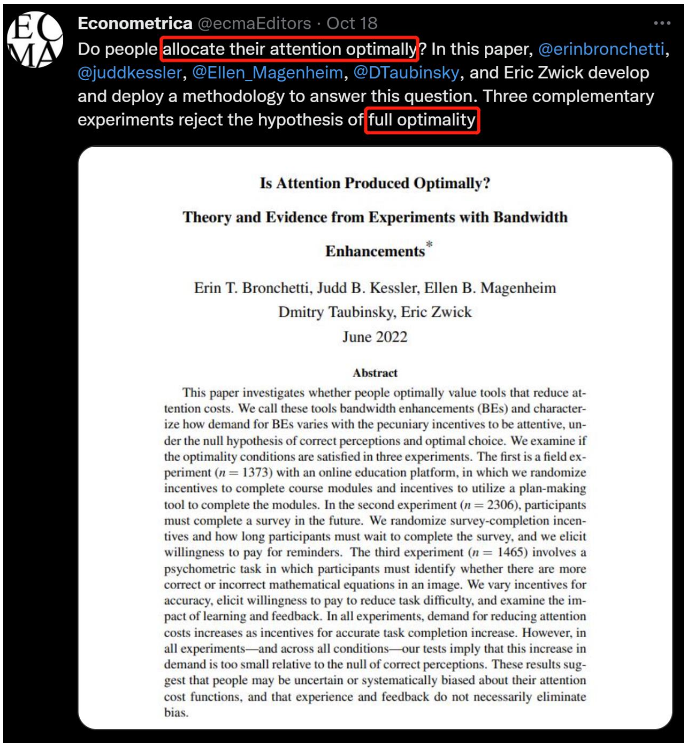

5. Your tweet:
6. Please provide one keyword about your job market paper
7. Please upload a picture of the title page of your job market paper (this will be included in the tweet).
8. Please upload a picture of the main graph/table/theorem of your job market paper (this will be included in the tweet)

1.4.8. Participation in mentoring workshops & open-ended questions.

1. What mentoring workshops, summer programs or job market panels have you attended?
   - AEA Summer and Scholarship Program
   - AEA Mentoring Program (CSMGEP)

- Association for Mentoring & Inclusion in Economics (AMIE) Mentoring Program
- CSQIEP LGBTQ+ Mentorship Program
- *#econ_prosem*
- Job Market Workshops by Economists for Ukraine
- Panel on the Job Market by Committee on the Status of LGBTQ+ Individuals in the Economics Profession (CSQIEP)
- Royal Economic Society (RES) Mentoring Programme
- Russell Sage Foundation Summer Institutes
- Southern Economic Association Mentoring Workshop
- Other, please specify
- I did not attend any workshops / summer program / job market panels

2. What questions do you want to ask the panelists regarding how to prepare for academic job interviews?
3. Do you have any comments or suggestions that you would like to share with the researchers who designed this survey? Is there anything you found unclear or confusing? Are there questions you wished we had asked? Please let us know what you think.

1.5. Empirical Strategy. We will estimate regression models of the following form using OLS:

$lm(DV \sim treat + X)$, where X is a matrix of pre-treatment covariates $lm(DV \sim treat + URG + treat * URG + X)$, where URG is a dummy variable for JMC from under-represented groups

Depending on the distribution of the outcome variables, we might perform robustness checks (e.g., negative binomial models for count data, log transformation for long-tail distributions etc.).

Anticipating one-sided non-compliance, we will estimate the intent-to-treat effect, and use our randomization as an instrument to estimate the local average treatment effects.

2. Email Invitation, Consent Form, Pre-market Survey

This appendix includes (1) the email invitation template sent to the influencers; (2) the consent form sent to job market candidates; and (3) the questions and instructions for the pre-market survey. 849 JMCs completed the pre-market survey. Among them, 590 submitted a tweet of their JMP. Among those who submitted a tweet, 519 participants posted their JMP online prior to the intervention, and were included in the intervention.[*] *We therefore report the summary statistics of these 519 participants, as they were later randomized into the treatment and control conditions.*

2.0.0.1. (1) Email Invitation Template:. *Below is the template for the email invitation to the influencers.*

Dear [FirstName],

Hope all is well. Al, Alain, Jingyi and I are working on a field experiment to evaluate the extent to which social media might be able to help job market candidates from under-represented groups (women, URM, LGBTQ+). We are going to recruit students on the econ job market this year, asking them to fill out a baseline survey and submit a summary of their job market paper in 280 characters. We will then tweet every submission from our Twitter account, Econ Job Market Helper.

Afterwards, we will randomize a subset of these JMPs to be retweeted by economist influencers in their field, such as yourself. Would you be willing to help us retweet (quote tweet) a number of these JMPs from your field? We will provide you with templates for the quote tweet. For example: "Retweeting a JMP from @econ_jmp to help publicize job market candidates in my field." The templates should make it clear that it is about exposure rather than endorsement. You can always edit the sentence if you like. Here is an example of a quote tweet.

We are not sure how many JMPs we will be sending your way at this point, but our best guess is somewhere between 10-20. We will get the list ready so that you just need to click on a link to quote tweet a JMP in your field. While we hope that you will retweet every JMP we send to you, you should feel free to choose which one(s) to retweet.

We are mindful that you are extremely busy, and would completely understand if you can't help out. Either way, please let me know. Thanks so much for considering our request!

Best,

Yan

On behalf of Alain Cohn, Jingyi Qiu and Al Roth

(2) Consent to Participate in Survey Research

Principal Investigators: Yan Chen (University of Michigan), Alain Cohn (University of Michigan), Jingyi Qiu (University of Michigan), Alvin Roth (Stanford University)

Online Consent

2.0.0.2. Description. This study is designed to understand social media exposure on job market outcomes. We therefore invite everyone who participates in the 2022-2023 economics job market to participate in our study. In this survey, you will be asked questions about your demographics, job market paper and the use of social media. Your responses will help us better understand the impact of social media on the job market outcomes.

2.0.0.3. Duration. The survey should take 10-15 minutes. Your participation is voluntary, and you may exit the survey at any time.

2.0.0.4. What will happen if you take part in this study. If you agree to participate, your responses might be linked to American Economic Association institutional records to draw information on job market outcomes. Once these data have been extracted, any information that can directly identify you will be removed from the data file and stored separately as part of the project.

2.0.0.5. What will be done with the information collected from the survey. The data are being collected by the research team (Yan Chen, Alain Cohn, Jingyi Qiu and Alvin Roth). Your responses will be held confidential. This means that only the research team will be able to access any identifying information about respondents. Additionally, your data will be anonymized and results will only be analyzed and reported at the aggregate level. Data may be stored in a repository for future research and you may be asked to participate in future surveys.

2.0.0.6. Benefits and risks of participation. By participating in this study, you will be invited to a virtual workshop on how to prepare for academic job interviews. We will also promote your job market paper on Twitter. Moreover, the results of this study has the potential to help future cohorts of job market candidates. This study involves no more than minimal risk. You may discontinue participation at any time during the research activity.

2.0.0.7. Contact information. If you have questions about this study, you may contact Jingyi Qiu (jaqiu@umich.edu).

The University of Michigan Institutional Review Board Health Sciences and Behavioral Sciences has determined that this study is no more than minimal risk and exempt from on-going IRB oversight (IRB Study Number: HUM00221663).

Please indicate below that you are at least 18 years old, have read and understand this consent form, and you agree to participate in this research study.

- No
- Yes

(3) Pre-Job-Market Survey and Summary Response Statistics

[*] We require JMPs be posted online for inclusion into our experiment so that influencers can read them before quote-tweeting.

2.1. Academic background.

1. Are you on the 2022-2023 job market?
   - Yes (100%)
   - No (0%)

   →If "No" is selected, skip to the end of the survey
2. Is this the first time you are on the Economics Job Market?
   - Yes (86.3%)
   - No (13.7%)
3. If no: Which year did you go on the Economics Job Market the first time?
4. What is the name of the institution at which you (are expected to) finish your PhD?
5. Have you ever been a full-time research assistant (also known as "pre-doc") before entering your PhD program?
   - Yes (17.7%)
   - No (82.3%)
6. If yes: what is/are the name(s) of your pre-doc advisor(s)?
   - Advisor 1's name:
   - Advisor 2's name:
   - Advisor 3's name:
   - Advisor 4's name:
7. Have you done (are you doing) a postdoc?
   - Yes (14.8%)
   - No (85.2%)

2.2. Field of specialty.

1. Please indicate your research fields (You can identify at most three research fields).
   - Behavioral economics (5.83%)
   - Development economics (7.41%)
   - Econometrics (4.16%)
   - Economic history (0.92%)
   - Economics of education (3.96%)
   - Environmental/Agricultural economics (4.66%)
   - Financial economics (5.00%)
   - Health economics (4.00%)
   - Industrial organization (5.41%)
   - Information economics (2.75%)
   - International economics (3.66%)
   - Labor economics (11.91%)
   - Law and economics (1.00%)
   - Macroeconomics (10.16%)
   - Microeconomics (8.66%)
   - Political economy (4.08%)
   - Public economics (7.08%)
   - Urban economics (3.91%)
   - Other, please specify (5.41%)
2. What are the names of your primary advisors?

- Advisor 1's name:
- Advisor 2's name:
- Advisor 3's name:
- Advisor 4's name:
- Advisor 5's name:

3. Please upload the latest version of your CV:
4. Do you have a personal webpage?
   - Yes (99.2%)
   - No (0.8%)
5. If yes, what is the URL of your personal webpage?

2.3. Support from advisors.

1. How satisfied are you with the support you received from your dissertation advisor(s) during your PhD? (1-7)
   Rating: Not at all satisfied (1) - Very satisfied (7)

   (Count 518, Mean 6.07, Std Dev 1.17, Median 6, Min 1, Max 7)

2.4. The types & locations of jobs you are looking for.

1. Please describe what types of jobs you are interested in / applying to:
   Rating: Not at all interested (1) - Very interested (7)
   - Assistant professor (tenure-track) at a college or university
     (Mean 6.63, Std Dev 0.96, Median 7, Min 1, Max 7)
   - Lecturer (non-tenure track) at college or university
     (Mean 3.24, Std Dev 1.92, Median 3, Min 1, Max 7)
   - Postdoctoral fellowship at college or university
     (Mean 4.67, Std Dev 1.88, Median 5, Min 1, Max 7)
   - Government, including central banks
     (Mean 4.76, Std Dev 1.92, Median 5, Min 1, Max 7)
   - Non-profit or quasi-governmental organizations (IMF, World Bank)
     (Mean 4.93, Std Dev 1.90, Median 5, Min 1, Max 7)
   - Contract research organizations / think tanks (e.g., RAND, Mathematica, Research Triangle Institute)
     (Mean 4.58, Std Dev 1.72, Median 5, Min 1, Max 7)
   - Private sector (e.g., banking, finance, tech, consulting, or other business/ industry)
     (Mean 3.99, Std Dev 1.85, Median 4, Min 1, Max 7)
2. In which geographic regions are you applying for jobs? You can select multiple choices.
   - U.S. (29.87%)
   - Other North America (20.10%)
   - Africa (0.32%)
   - Asia (9.70%)
   - Oceania/Australia/New Zealand (11.55%)
   - South America (4.08%)
   - Europe (23.99%)
   - Other, please specify (0.38%)

2.5. Twitter account.

1. Do you have a Twitter account?
   - Yes (69.7%)
   - No (30.3%)
2. If yes: What is your Twitter handle?
   Your Twitter handle (e.g., @abc):

2.6. Demographics.

1. What is your gender?
    - Woman (29.67%)
    - Man (67.82%)
    - Non-Binary (0.39%)
    - Prefer not to say (2.12%)
2. Do you consider yourself to be transgender?
    - Yes, I am transgender (0.39%)
    - No, I am cisgender (72.64%)
    - I do not consider myself either transgender or cisgender (19.65%)
    - Don't know / Not sure (1.54%)
    - Prefer not to say (5.78%)
3. What is your sexual orientation?
    - Straight (84.59%)
    - Gay (2.50%)
    - Lesbian (0.39%)
    - Bisexual (1.35%)
    - Pansexual (0.19%)
    - Queer (0.58%)
    - Asexual (0.19%)
    - Prefer not to say (10.21%)
4. In what year were you born?
    - I was born in... YYYY (98.84%)
    - Prefer not to say (1.16%)
5. What is your country of citizenship? (If you have dual citizenship then please indicate your primary citizenship.)
   - - Select Country - -
6. Are you of Hispanic, Latino, or Spanish origin?
    - Yes (13.10%)
    - No (84.01%)
    - Prefer not to say (2.89%)
7. What is the primary ethnicity or race you identify with?
    - White (49.71%)
    - Black or African American (2.31%)
    - American Indian or Alaska Native (0.38%)
    - Asian (36.42%)
    - Native Hawaiian or Other Pacific Islander (0%)
    - Other, please specify (5.20%)
    - Prefer not to say (5.97%)
8. What was the highest level of education achieved by your mother?
    - Less than high school (10.02%)
    - High school diploma or equivalent (17.73%)
    - Associate's degree (6.55%)
    - Bachelor's degree (28.71%)
    - Master's degree (20.42%)

- Professional degree (e.g. MBA, J.D., M.D.) (5.01%)
- Doctoral degree (e.g., Ph.D., Ed.D.) (9.25%)
- Don't know (0.19%)
- Prefer not to say (2.12%)

9. How would you describe the financial situation of your family when you grew up?

- High income / wealthy (3.28%)
- Upper middle class (30.25%)
- Middle class (46.63%)
- Low income (14.07%)
- In poverty (2.70%)
- Prefer not to say (3.08%)

2.7. Instructions for writing a job market paper tweet. As a thank you for participation, we will post a tweet about your job market paper (JMP) on our Twitter account "Econ Job Market Helper" (@*econ_jmp*). This account is already followed by some influential economists.

Because you know your JMP best, we are asking you to prepare the text that we will use verbatim for the tweet. It should describe the main idea and/or result of your job market paper. Note, the text limit is 280 characters. You can also use emojis (each emoji counts as two characters). Below are some guiding questions:

- What is the research question? Try to frame it in a broader context so that the tweet will create more interest.
- What data/method/modeling approach do you use and what is your main finding? Try to state your data/method/modeling approach and finding(s) in a precise way.
- Add a link to your personal website and, if you have a Twitter account, your Twitter handle (e.g., @abc).

Here are some additional tips to improve the quality of your tweet:
Do's:

1. Use numbers if applicable

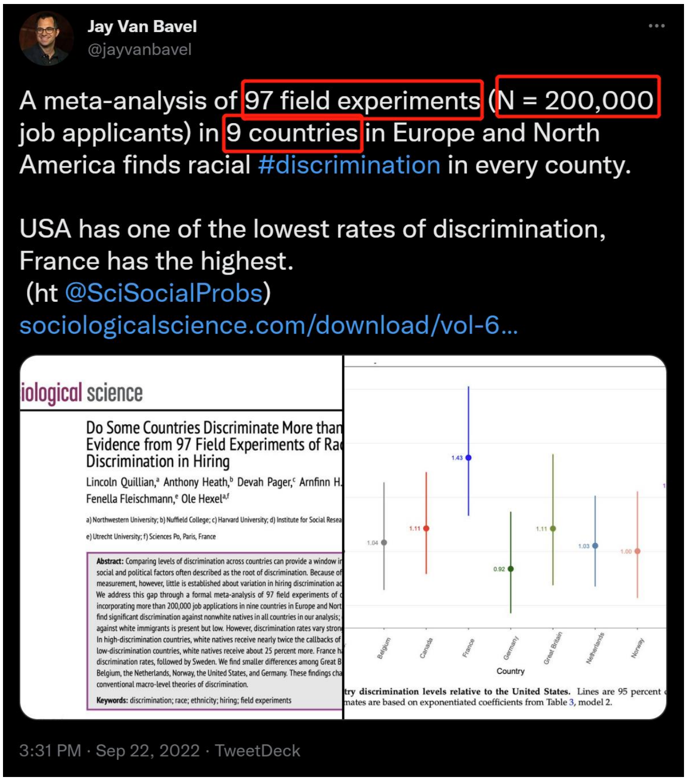

2. Use open ended questions

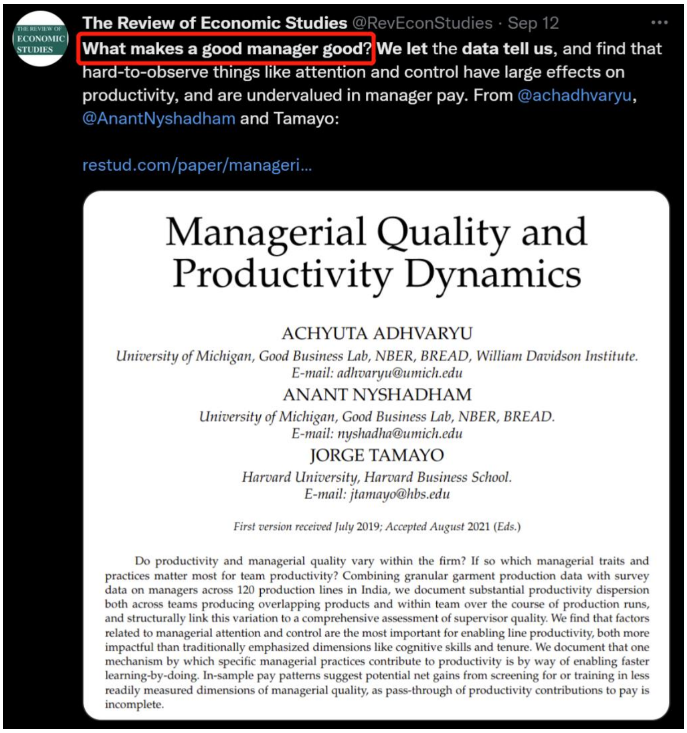

The Review of Economic Studies @RevEconStudies · Sep 12

What makes a good manager good? We let the **data tell us**, and find that hard-to-observe things like attention and control have large effects on productivity, and are undervalued in manager pay. From @achadhvaryu, @AnantNyshadham and Tamayo:

restud.com/paper/manageri...

Managerial Quality and Productivity Dynamics

ACHYUTA ADHVARYU

University of Michigan, Good Business Lab, NBER, BREAD, William Davidson Institute.
E-mail: adhvaryu@umich.edu

ANANT NYSHADHAM

University of Michigan, Good Business Lab, NBER, BREAD.
E-mail: nyshadha@umich.edu

JORGE TAMAYO

Harvard University, Harvard Business School.
E-mail: jtamayo@hbs.edu

First version received July 2019; Accepted August 2021 (Eds.)

Do productivity and managerial quality vary within the firm? If so which managerial traits and practices matter most for team productivity? Combining granular garment production data with survey data on managers across 120 production lines in India, we document substantial productivity dispersion both across teams producing overlapping products and within team over the course of production runs, and structurally link this variation to a comprehensive assessment of supervisor quality. We find that factors related to managerial attention and control are the most important for enabling line productivity, both more impactful than traditionally emphasized dimensions like cognitive skills and tenure. We document that one mechanism by which specific managerial practices contribute to productivity is by way of enabling faster learning-by-doing. In-sample pay patterns suggest potential net gains from screening for or training in less readily measured dimensions of managerial quality, as pass-through of productivity contributions to pay is incomplete.

3. Use emojis

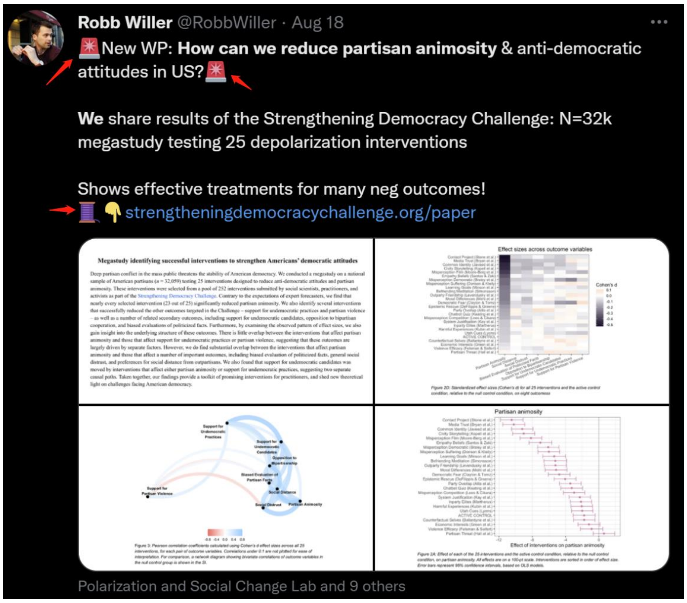

4. Focus on first and last words

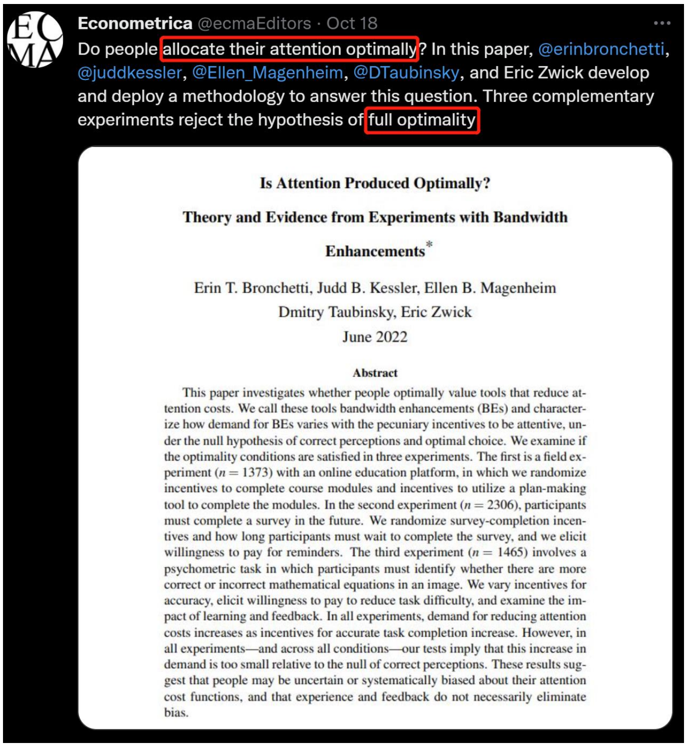

5. Your tweet:

6. Please provide one keyword about your job market paper

7. Please upload a picture of the title page of your job market paper (this will be included in the tweet).

8. Please upload a picture of the main graph/table/theorem of your job market paper (this will be included in the tweet)

2.8. Participation in mentoring workshops & open-ended questions.

1. What mentoring workshops, summer programs or job market panels have you attended?

   - AEA Summer and Scholarship Program (1.66%)

- AEA Mentoring Program (CSMGEP) (1.11%)
- Association for Mentoring & Inclusion in Economics (AMIE) Mentoring Program (2.59%)
- CSQIEP LGBTQ+ Mentorship Program (1.29%)
- *#econ_prosem* (3.33%)
- Job Market Workshops by Economists for Ukraine (6.28%)
- Panel on the Job Market by Committee on the Status of LGBTQ+ Individuals in the Economics Profession (CSQIEP) (0.55%)
- Royal Economic Society (RES) Mentoring Programme (3.14%)
- Russell Sage Foundation Summer Institutes (1.29%)
- Southern Economic Association Mentoring Workshop (0.55%)
- Other, please specify (16.82%)
- I did not attend any workshops / summer program / job market panels (61.37%)

2. What questions do you want to ask the panelists regarding how to prepare for academic job interviews?

3. Do you have any comments or suggestions that you would like to share with the researchers who designed this survey? Is there anything you found unclear or confusing? Are there questions you wished we had asked? Please let us know what you think.

3. Figures and Tables

Fig. S1. Influencers' Twitter Follower Counts vs. Citation Counts

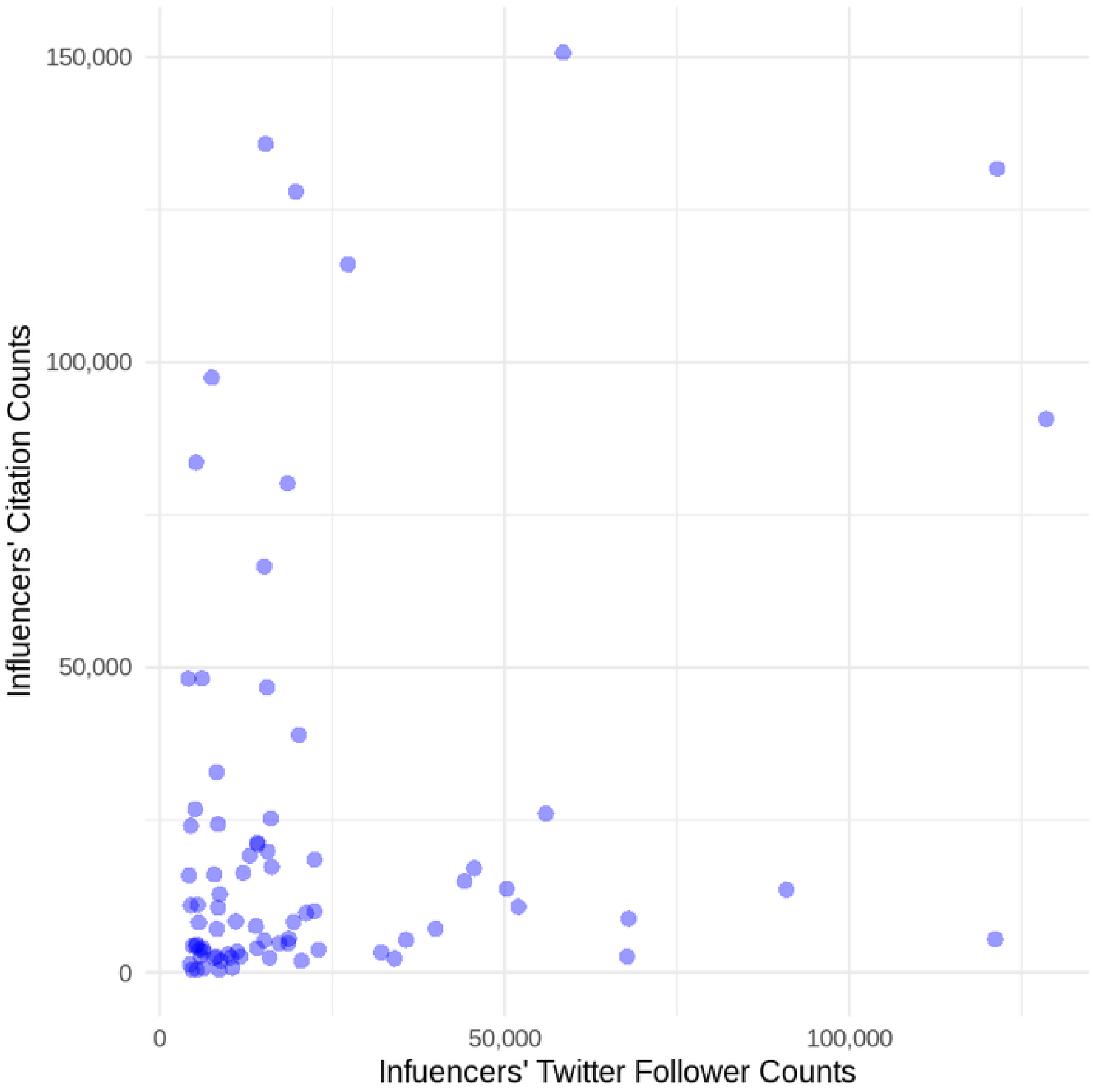

Notes: The Pearson correlation between influencers' Twitter follower counts and citation counts is 0.286.

Fig. S2. A Made-up Quote-tweet Example

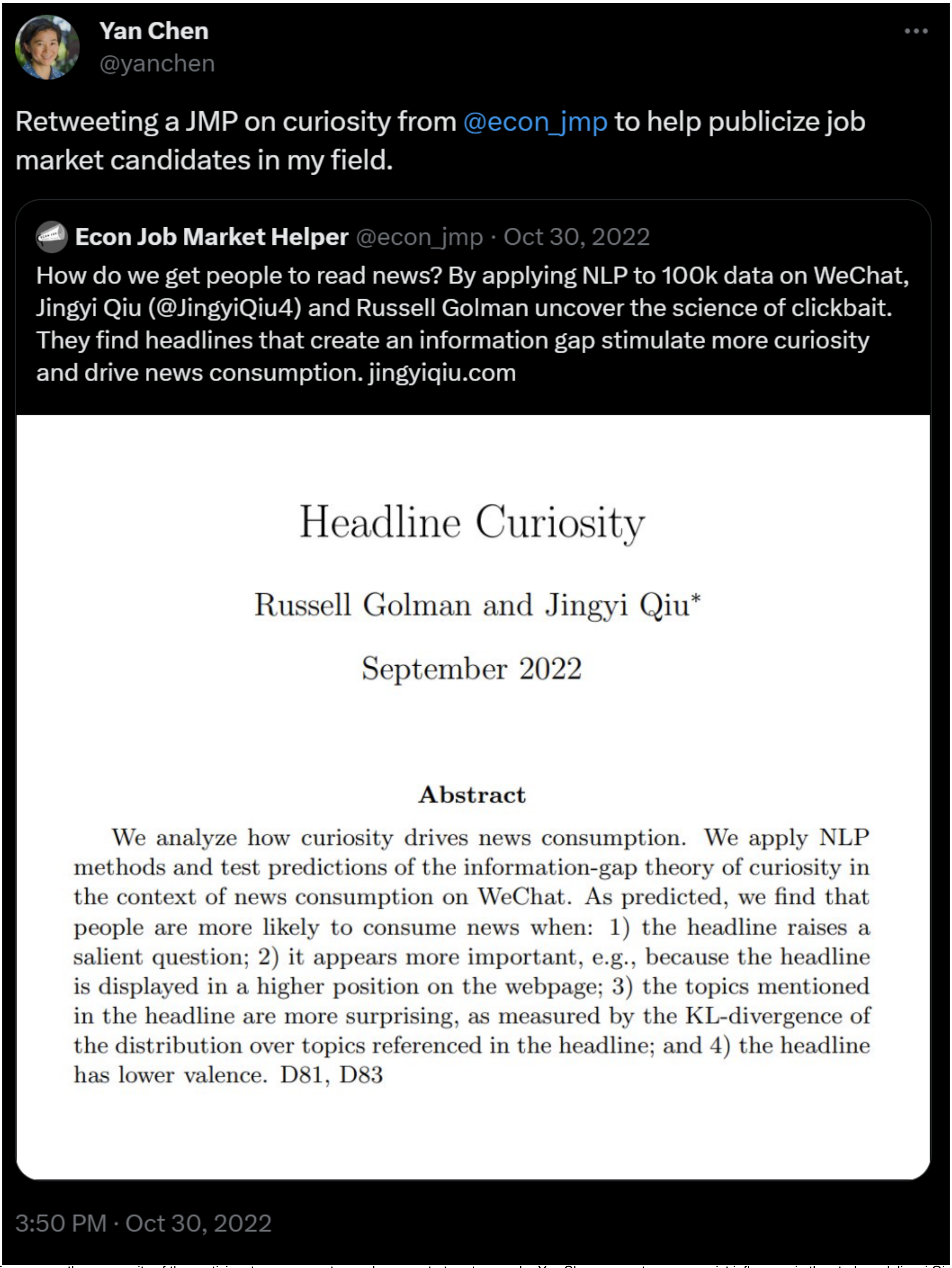

Notes: To preserve the anonymity of the participants, we present a made-up quote-tweet example. Yan Chen was not an economist influencer in the study and Jingyi Qiu was not a job market candidate in 2022-23.

Fig. S3. Distribution of Self-reported Pre-tax Annual Salary (in USD) (n = 281)

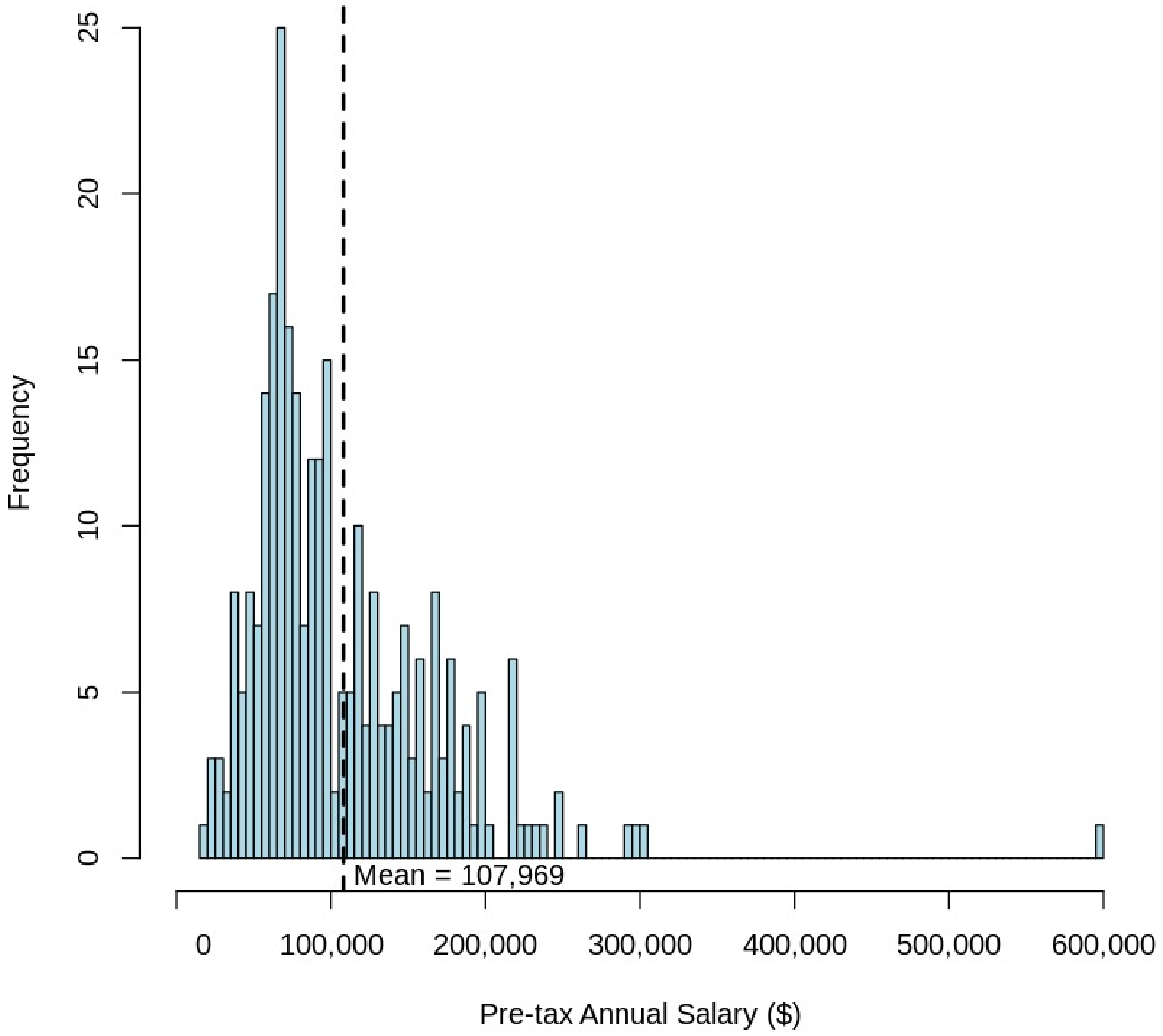

Notes: We asked participants to report their pre-tax annual salaries in thousands of US dollars, but 148 participants potentially reported in dollars rather than thousands. We divided salaries above 9,900 by 1,000 to reconcile the inconsistency.

Fig. S4. Placement Satisfaction (ITT)

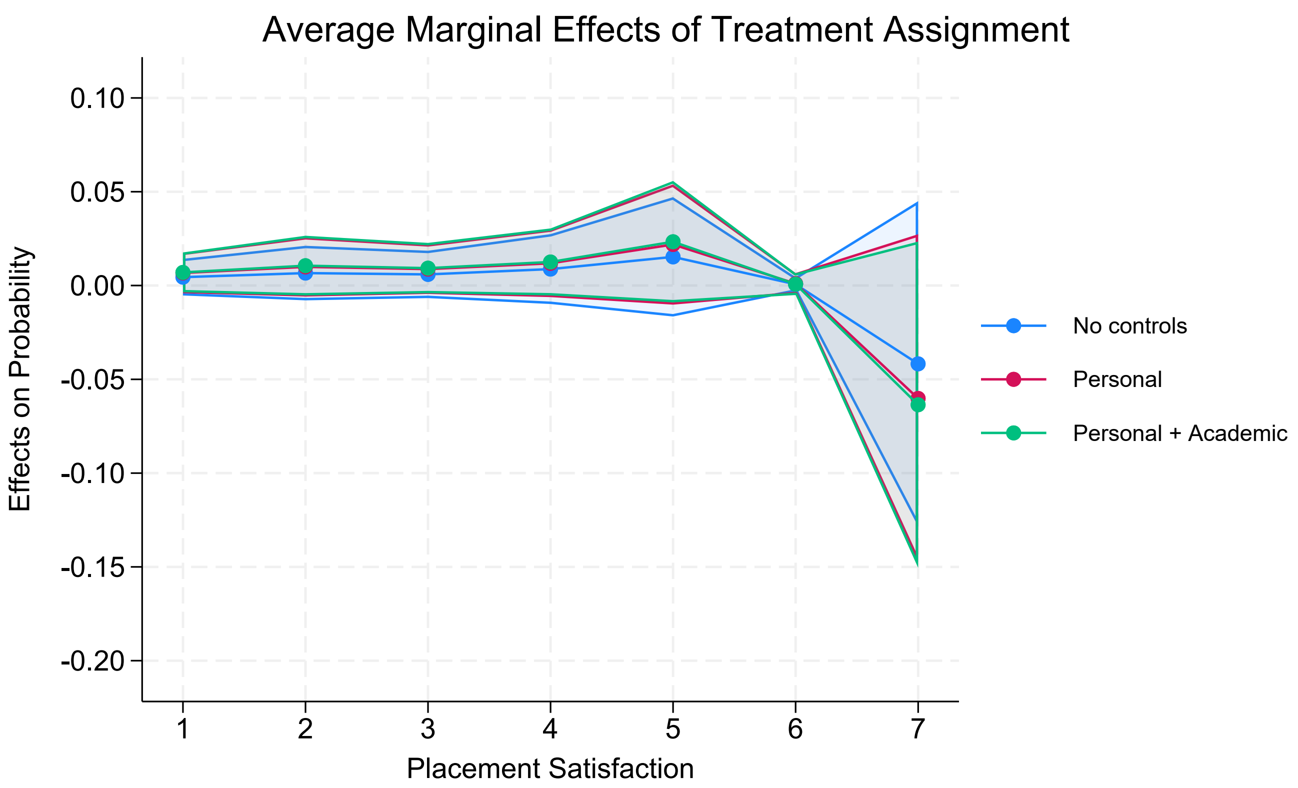

Notes: Ordered Probit estimates of intent-to-treat (ITT) effects on placement satisfaction. Placement satisfaction is measured on a 7-point Likert scale from '1' (Not satisfied at all) to '7' (Very satisfied). Sample size: n=378.

Table S1. Post-Market Survey Completion

Dependent variable:	Completed Post-Market Survey (=1)	
	(1)	(2)
Treatment group	0.043	0.042
	(0.038)	(0.038)
URG	0.058	0.065*
	(0.038)	(0.039)
Age		-0.008
		(0.005)
Parental income class		-0.010
		(0.022)
US citizen		0.113***
		(0.040)
Twitter account		0.083**
		(0.042)
Top 30 PhD institution		0.011
		(0.037)
First-time JMC		0.009
		(0.068)
Predoc		0.052
		(0.043)
Postdoc		0.004
		(0.060)
Constant	0.758***	0.923***
	(0.028)	(0.200)
Observations	519	500
Sample	Pre-market	Pre-market

Notes: This table presents the post-market survey completion rates across the treatment and control groups. Both specifications include a dummy variable indicating whether an individual is classified as belonging to underrepresented groups (URG) to account for the varying probability of treatment assignment. Column 2 controls for participants' personal and academic background characteristics. Personal background characteristics include age, parental income class (on a 0-4 scale, coded as follows: 4 for "High income / wealthy", 3 for "Upper middle class", 2 for "Middle class", 1 for "Low income", and 0 for "In poverty"), US citizenship status, and Twitter account ownership, while academic background characteristics include graduating from a top 30 PhD institution (in or outside the US), being on the job market for the first time, as well as predoctoral and postdoctoral experience. OLS estimates with robust standard errors in parentheses. Significance levels: * $p < 0.10$, ** $p < 0.05$, *** $p < 0.01$.

Table S2. Descriptive Statistics for URG

Variable	Control	Treatment	Total	p-value
**Pre-market Sample**				
US citizen	9 (11.5%)	19 (12.9%)	28 (12.4%)	0.761
Twitter account	59 (75.6%)	111 (75.5%)	170 (75.6%)	0.983
Top 30 PhD institution	32 (41%)	63 (42.9%)	95 (42.2%)	0.791
First-time JMC	69 (88.5%)	130 (88.4%)	199 (88.4%)	0.995
Predoc	18 (23.1%)	28 (19%)	46 (20.4%)	0.485
Postdoc	7 (9%)	21 (14.3%)	28 (12.4%)	0.221
Age	31.69 (4.22)	31.96 (4.40)	31.87 (4.33)	0.654
Parental income class	2.20 (0.76)	2.09 (0.84)	2.13 (0.81)	0.318
**Post-market Sample**				
US citizen	8 (13.1%)	18 (14%)	26 (13.7%)	0.874
Twitter account	48 (78.7%)	100 (77.5%)	148 (77.9%)	0.855
Top 30 PhD institution	25 (41%)	54 (41.9%)	79 (41.6%)	0.909
First-time JMC	57 (93.4%)	114 (88.4%)	171 (90%)	0.232
Predoc	17 (27.9%)	26 (20.2%)	43 (22.6%)	0.252
Postdoc	5 (8.2%)	19 (14.7%)	24 (12.6%)	0.164
Age	31.15 (3.53)	32.01 (4.54)	31.73 (4.25)	0.157
Parental income class	2.22 (0.74)	2.08 (0.84)	2.12 (0.81)	0.253
# applications (1-50)	8 (13.1%)	16 (12.5%)	24 (12.7%)	0.906
# applications (51-100)	11 (18%)	20 (15.6%)	31 (16.4%)	0.682
# applications (101-150)	13 (21.3%)	28 (21.9%)	41 (21.7%)	0.930
# applications (151-200)	18 (29.5%)	27 (21.1%)	45 (23.8%)	0.220
# applications (200+)	11 (18%)	37 (28.9%)	48 (25.4%)	0.087
AEA signal sent: 2	48 (78.7%)	99 (77.3%)	147 (77.8%)	0.834
Publication or R&R (1+)	34 (56.7%)	76 (59.8%)	110 (58.8%)	0.681

Note: The table shows descriptive statistics for each control variable by underrepresented group (URG) in the pre-market and post-market samples. For continuous variables (Age, Parental income class), means and standard deviations are reported in the format "Mean (SD)" and p-values are from two-sample t-tests. For all other binary variables, counts and percentages are reported in the format "Count (Proportion%)" and p-values are from two-sample z-tests for the difference in proportions. In the pre-market sample (including both URG and non-URG), there are 6 missing observations for "Age" and 16 for "Parental Income Status". The post-market sample has the following missing observations: 4 for "Age", 11 for "Parental income class", 9 for "Publication or R&R (1+)", 4 for "# applications", and 4 for "AEA signals (2)".

Table S3. Descriptive Statistics for non-URG

Variable	Control	Treatment	Total	p-value
**Pre-market Sample**				
US citizen	49 (24.9%)	17 (17.5%)	66 (22.4%)	0.137
Twitter account	129 (65.5%)	63 (64.9%)	192 (65.3%)	0.928
Top 30 PhD institution	95 (48.2%)	47 (48.5%)	142 (48.3%)	0.970
First-time JMC	167 (84.8%)	83 (85.6%)	250 (85%)	0.856
Predoc	29 (14.7%)	17 (17.5%)	46 (15.6%)	0.543
Postdoc	32 (16.2%)	17 (17.5%)	49 (16.7%)	0.784
Age	31.20 (3.19)	31.87 (3.77)	31.42 (3.40)	0.136
Parental income class	2.26 (0.80)	2.14 (0.89)	2.22 (0.83)	0.276
**Post-market Sample**				
US citizen	42 (27.6%)	16 (21.3%)	58 (25.6%)	0.291
Twitter account	103 (67.8%)	51 (68%)	154 (67.8%)	0.971
Top 30 PhD institution	76 (50%)	38 (50.7%)	114 (50.2%)	0.925
First-time JMC	128 (84.2%)	65 (86.7%)	193 (85%)	0.617
Predoc	23 (15.1%)	13 (17.3%)	36 (15.9%)	0.675
Postdoc	24 (15.8%)	12 (16%)	36 (15.9%)	0.967
Age	31.00 (3.15)	31.77 (3.54)	31.25 (3.29)	0.115
Parental income class	2.31 (0.80)	2.07 (0.89)	2.23 (0.83)	0.051
# applications (1-50)	21 (14%)	6 (8.1%)	27 (12.1%)	0.166
# applications (51-100)	27 (18%)	13 (17.6%)	40 (17.9%)	0.936
# applications (101-150)	43 (28.7%)	27 (36.5%)	70 (31.2%)	0.243
# applications (151-200)	25 (16.7%)	11 (14.9%)	36 (16.1%)	0.726
# applications (200+)	34 (22.7%)	17 (23%)	51 (22.8%)	0.959
AEA signal sent: 2	111 (74%)	57 (77%)	168 (75%)	0.617
Publication or R&R (1+)	90 (60.8%)	42 (57.5%)	132 (59.7%)	0.642

Note: The table shows descriptive statistics for each control variable by non-underrepresented group (non-URG) in the pre-market and post-market samples. For continuous variables (Age, Parental income class), means and standard deviations are reported in the format "Mean (SD)" and p-values are from two-sample t-tests. For all other binary variables, counts and percentages are reported in the format "Count (Proportion%)" and p-values are from two-sample z-tests for the difference in proportions. In the pre-market sample (including both URG and non-URG), there are 6 missing observations for "Age" and 16 for "Parental Income Status". The post-market sample has the following missing observations: 4 for "Age", 11 for "Parental income class", 9 for "Publication or R&R (1+)", 4 for "# applications", and 4 for "AEA signals (2)".

Table S4. Descriptive Statistics for Pre- and Post-market Survey Samples

Variable	Pre-market (n=519)	Post-market (n=417)
URG	225 (43%)	190 (46%)
Age	31.6 (3.8)	31.5 (3.8)
Parental income class	2.2 (0.8)	2.2 (0.8)
US citizen	94 (18%)	84 (20%)
Twitter account	362 (70%)	302 (72%)
Top 30 PhD institution	237 (46%)	193 (46%)
First-time JMC	449 (87%)	364 (87%)
Predoc	92 (18%)	79 (19%)
Postdoc	77 (15%)	60 (14%)
Publication or R&R (1+)	—	242 (59%)
# applications (1–50)	—	51 (12%)
# applications (51–100)	—	71 (17%)
# applications (101–150)	—	111 (27%)
# applications (151–200)	—	81 (20%)
# applications (201+)	—	99 (24%)
AEA signals (2)	—	315 (76%)

Notes: The table presents descriptive statistics for control variables in the pre-market and post-market samples. For continuous variables, means and standard deviations are reported; for binary variables, counts and percentages are provided. In the pre-market sample, there are 6 missing observations for "Age" and 16 for "Parental Income Status". The post-market sample has the following missing observations: 4 for "Age", 11 for "Parental income class", 9 for "Publication or R&R (1+)", 4 for "# applications", and 4 for "AEA signals (2)".

Table S5. Comparison Between Our Sample and AEA Sample

Characteristics	Our Sample (in %) n = 519	AEA Sample (in %) n = 623	p-values
Gender Identity - Female	29.7	28.1	0.557
Gender Identity - Male	67.8	59.9	0.005
Gender Identity - Non-Binary / Agender / Something else	0.4	0.6	0.561
Gender Identity - Prefer not to say	2.1	2.3	0.881
Gender Identity - Missing data	0	9.2	<0.001
Sexual Orientation - Heterosexual / Straight	84.6	75.4	<0.001
Sexual Orientation - Bisexual	1.4	4.8	0.001
Sexual Orientation - Gay / Lesbian	2.9	4.0	0.305
Sexual Orientation - Something else / Unsure	1.0	1.0	1.0
Sexual Orientation - Prefer not to say	10.2	5.6	0.004
Sexual Orientation - Missing data	0	9.2	<0.001
Transgender - No	92.3	87.5	0.008
Transgender - Don't know / Not sure	1.5	0.6	0.137
Transgender - Yes, Transgender, gender nonconforming	0.4	0.2	0.451
Transgender - Prefer not to say	5.8	2.6	0.006
Transgender - Missing data	0	9.2	<0.001
Hispanic - Yes	13.1	13.6	0.790
Hispanic - No	84.0	75.0	<0.001
Hispanic - Prefer not to say	2.9	2.6	0.740
Hispanic - Missing data	0	8.8	<0.001
US Citizenship - Yes	18.1	33.6	<0.001
US Citizenship - No	81.9	58.3	<0.001
US Citizenship - Missing data	0	8.2	<0.001
Race - White	49.7	51.0	0.654
Race - Asian	36.4	27.0	0.001
Race - Black or African American	2.3	2.7	0.653
Race - Other	5.6	2.6	0.009
Race - Mixed race		2.6	
Race - Prefer not to say	6.0	5.3	0.624
Race - Missing data	0	8.8	<0.001
Age - Mean (Std Dev)	31.6 (3.8)	33.1 (5.1)	
Age - Missing data (in %)	1.2	20.4	

Notes: This table compares the percentages of participants with different demographic characteristics between the AEA sample and our sample. The p-values are calculated using z-tests for the difference in proportions. our sample has fewer US citizens (18% vs. 34%, $p < 0.001$), likely due to our drawing of participants from both the AEA JOE and the European Job Market. In addition, the AEA survey sample has some missing data related to underrepresented group categories and US citizenship, which may also explain some differences between our descriptive statistics and the AEA survey. In our sample, the sum exceeding 100% in the "Sexual Orientation" category is attributed to the rounding of decimal figures to the nearest tenth. Specifically, "Sexual Orientation - Bisexual" was reported as 1.35% but rounded to 1.4%, and "Sexual Orientation - Something else / Unsure" was reported as 0.96% but rounded to 1.0%. When these rounded figures are added together with the other categories, the total exceeds 100% by a slight margin. In our sample, 19.65% of participants chose the option "I do not consider myself either transgender or cisgender" in response to the question about their transgender identity, likely due to a lack of understanding of the terminology. Thus, we merged this category with "No, I am cisgender" for analysis purposes. Notably, the AEA survey does not include the option "I do not consider myself either transgender or cisgender." For the question about race, since the AEA survey does not provide the option "American Indian or Alaska Native," we combined responses from this category with those from the "Other" category in this table.

Table S6. Number of Views (ITT)

**Dependent variable:**	**Number of Views**					
	(1)	(2)	(3)	(4)	(5)	(6)
Treatment group	3907.186***	3955.413***	3969.650***	3431.105***	3456.410***	3467.637***
	(501.988)	(518.344)	(524.201)	(421.077)	(434.121)	(432.829)
URG	492.052	390.809	463.512	511.997	410.163	512.531
	(513.045)	(512.387)	(519.259)	(448.593)	(429.776)	(440.544)
Age		-85.556*	-110.429**		-1.477	-40.850
		(50.110)	(53.845)		(52.635)	(57.038)
Parental income class		219.921	245.115		224.662	235.522
		(239.199)	(248.966)		(241.974)	(252.678)
US citizen		-725.687*	-788.243*		-396.101	-529.516
		(431.458)	(429.099)		(410.886)	(415.916)
Twitter account		773.924*	852.422**		772.451**	924.491**
		(429.406)	(433.291)		(373.152)	(394.673)
Top 30 PhD institution			130.520			302.909
			(502.443)			(480.727)
First-time JMC			-1233.218			-1912.072*
			(870.146)			(1105.566)
Predoc			-742.502			-191.900
			(506.940)			(536.762)
Postdoc			-459.400			-735.878
			(617.476)			(745.602)
Constant	734.320***	2602.795	4460.413**	756.921***	-117.956	2631.277
	(192.211)	(1713.432)	(1914.762)	(202.949)	(1937.674)	(2033.670)
Observations	519	500	500	417	403	403
Sample	Pre-market	Pre-market	Pre-market	Post-market	Post-market	Post-market

Notes: This table presents the intent-to-treat (ITT) effect of being quote-tweeted by established economists on Twitter on the number of views received by job market paper tweets (which naturally include views of quote-tweets). Columns 1-3 present the results for the pre-market sample, which comprises 519 participants who completed our pre-market survey. Columns 4-6 present the results for the post-market sample, which includes 417 participants who reported their job market outcomes. All specifications include a dummy variable indicating whether an individual is classified as belonging to underrepresented groups (URG) to account for the varying probability of treatment assignment. Columns 2 and 5 control for participants' personal background characteristics, including age, parental income class (on a 0-4 scale, coded as follows: 4 for "High income / wealthy", 3 for "Upper middle class", 2 for "Middle class", 1 for "Low income", and 0 for "In poverty"), US citizenship status, and Twitter account ownership. Columns 3 and 6 additionally control for participants' academic background, including graduating from a top 30 PhD institution (in or outside the US), being on the job market for the first time, as well as predoctoral and postdoctoral experience. OLS estimates with robust standard errors in parentheses. Significance levels: * $p < 0.10$, ** $p < 0.05$, *** $p < 0.01$.

Table S7. Number of Likes (ITT)

Dependent variable:	Number of Likes					
	(1)	(2)	(3)	(4)	(5)	(6)
Treatment group	12.021***	11.777***	11.782***	11.540***	11.103***	11.101***
	(1.746)	(1.725)	(1.723)	(1.574)	(1.516)	(1.520)
URG	1.274	1.160	1.255	0.191	0.043	0.076
	(1.776)	(1.788)	(1.844)	(1.580)	(1.594)	(1.646)
Age		-0.466**	-0.470**		-0.168	-0.175
		(0.203)	(0.203)		(0.177)	(0.165)
Parental income class		-0.016	-0.186		-0.302	-0.526
		(0.889)	(0.923)		(0.982)	(0.997)
US citizen		-2.590*	-2.760*		-1.871	-2.077
		(1.471)	(1.458)		(1.420)	(1.393)
Twitter account		2.173	2.463		1.831	2.182
		(1.513)	(1.526)		(1.505)	(1.501)
Top 30 PhD institution			1.933			1.633
			(1.698)			(1.566)
First-time JMC			-2.197			-2.304
			(1.846)			(2.285)
Predoc			-0.928			1.029
			(1.797)			(1.887)
Postdoc			-2.397			-2.536
			(1.641)			(1.947)
Constant	3.388***	17.290**	19.099***	3.795***	9.022	10.930*
	(0.759)	(6.728)	(6.987)	(0.850)	(6.656)	(6.285)
Observations	519	500	500	417	403	403
Sample	Pre-market	Pre-market	Pre-market	Post-market	Post-market	Post-market

Notes: This table presents the intent-to-treat (ITT) effect of being quote-tweeted by established economists on Twitter on the sum of likes received by the job market paper tweets and the influencer quote-tweets. For a description of the variables, see the table notes in Appendix Table S6. OLS estimates with robust standard errors in parentheses. Significance levels: * $p < 0.10$, ** $p < 0.05$, *** $p < 0.01$.

Table S8. Number of Views (LATE)

**Dependent variable:**	**Number of Views**					
	(1)	(2)	(3)	(4)	(5)	(6)
Treatment	4413.850***	4455.248***	4475.910***	3858.257***	3868.918***	3885.121***
	(557.561)	(572.556)	(577.769)	(459.890)	(471.130)	(467.649)
URG	605.038	542.157	595.322	589.348	528.609	610.101
	(503.354)	(498.572)	(504.803)	(438.432)	(417.512)	(428.118)
Age		-84.734*	-108.369**		-2.673	-40.550
		(49.398)	(53.357)		(52.231)	(56.679)
Parental income class		220.585	237.950		212.500	214.277
		(228.331)	(236.585)		(231.433)	(240.174)
US citizen		-722.525*	-783.374*		-370.700	-498.805
		(422.838)	(419.052)		(399.046)	(401.442)
Twitter account		632.238	702.840*		653.474*	798.136**
		(413.040)	(414.268)		(352.257)	(372.440)
Top 30 PhD institution			61.266			260.602
			(490.978)			(464.334)
First-time JMC			-1199.114			-1827.406*
			(846.822)			(1077.143)
Predoc			-579.908			-34.599
			(477.031)			(504.338)
Postdoc			-545.118			-728.134
			(611.704)			(730.106)
Constant	702.273***	2630.258	4463.132**	734.769***	-8.308	2640.532
	(190.896)	(1678.280)	(1899.506)	(201.503)	(1913.501)	(2017.632)
First stage instrument:						
Treatment assignment	0.885	0.888	0.887	0.885	0.888	0.893
Standard error	0.021	0.021	0.021	0.021	0.021	0.023
F-statistics	1789.836	1826.429	1789.442	1505.779	1584.249	1544.164
Observations	519	500	500	417	403	403
Sample	Pre-market	Pre-market	Pre-market	Post-market	Post-market	Post-market

Notes: This table presents the local average treatment effect (LATE) of being quote-tweeted by established economists on Twitter on the number of views received by job market paper tweets (which naturally include views of quote-tweets). For a description of the variables, see the table notes in Appendix Table S6. 2SLS estimates with Robust standard errors in parentheses. Significance levels: * $p < 0.10$, ** $p < 0.05$, *** $p < 0.01$.

Table S9. Number of Likes (LATE)

**Dependent variable:**	**Number of Likes**					
	(1)	(2)	(3)	(4)	(5)	(6)
Treatment	13.429***	13.112***	13.135***	12.771***	12.219***	12.226***
	(1.940)	(1.906)	(1.899)	(1.742)	(1.671)	(1.664)
URG	1.943	1.868	1.898	0.870	0.786	0.784
	(1.716)	(1.710)	(1.760)	(1.541)	(1.545)	(1.594)
Age		-0.507**	-0.504**		-0.236	-0.233
		(0.198)	(0.197)		(0.176)	(0.163)
Parental income class		-0.247	-0.441		-0.663	-0.916
		(0.867)	(0.895)		(0.962)	(0.971)
US citizen		-2.627*	-2.799**		-1.868	-2.074
		(1.427)	(1.406)		(1.375)	(1.337)
Twitter account		1.882	2.164		1.631	1.998
		(1.463)	(1.463)		(1.446)	(1.434)
Top 30 PhD institution			2.005			1.933
			(1.632)			(1.522)
First-time JMC			-2.229			-2.210
			(1.806)			(2.234)
Predoc			-0.569			1.297
			(1.695)			(1.791)
Postdoc			-2.729*			-2.642
			(1.635)			(1.900)
Constant	3.334***	19.226***	20.851***	3.782***	12.040*	13.448**
	(0.758)	(6.600)	(6.817)	(0.849)	(6.693)	(6.265)
First stage instrument:						
Treatment assignment	0.883	0.885	0.885	0.883	0.885	0.893
Standard error	0.021	0.021	0.021	0.021	0.021	0.023
F-statistics	1774.466	1806.236	1774.707	1522.059	1596.866	1561.949
Observations	523	504	504	420	406	406
Sample	Pre-market	Pre-market	Pre-market	Post-market	Post-market	Post-market

Notes: This table presents the local average treatment effect (LATE) of being quote-tweeted by established economists on Twitter on the sum of likes received by the job market paper tweets and the influencer quote-tweets. For a description of the variables, see the table notes in Appendix Table S6. 2SLS estimates with robust standard errors in parentheses. Significance levels: * $p < 0.10$, ** $p < 0.05$, *** $p < 0.01$.

Table S10. Number of Interviews (ITT)

**Dependent variable:**	**Number of Interviews**					
	All Jobs			Tenure-Track Jobs		
	(1)	(2)	(3)	(4)	(5)	(6)
Treatment group	1.317	2.002	1.222	1.377	1.747	1.091
	(1.253)	(1.295)	(1.132)	(1.081)	(1.128)	(1.024)
URG	1.101	1.104	0.776	0.315	0.150	0.129
	(1.271)	(1.285)	(1.138)	(1.084)	(1.118)	(1.038)
Age		-0.669***	-0.268**		-0.428***	-0.168
		(0.139)	(0.130)		(0.128)	(0.123)
Parental income class		0.201	-0.529		-0.379	-0.838
		(0.754)	(0.699)		(0.637)	(0.598)
US citizen		2.069	1.544		-0.535	-0.983
		(1.479)	(1.308)		(1.208)	(1.136)
Twitter account		2.934**	2.863**		1.731	1.889*
		(1.241)	(1.117)		(1.068)	(0.974)
Top 30 PhD institution			5.819***			6.022***
			(1.133)			(1.025)
First-time JMC			2.512**			2.039*
			(1.168)			(1.165)
Predoc			0.429			-0.153
			(1.318)			(1.147)
Postdoc			-4.238***			-2.207**
			(1.043)			(1.024)
Publication or R&R (1+)			3.420***			3.852***
			(1.123)			(1.001)
# applications (51-100)			5.898***			4.473***
			(1.484)			(1.416)
# applications (101-150)			7.319***			6.040***
			(1.416)			(1.331)
# applications (151-200)			9.921***			7.044***
			(1.788)			(1.615)
# applications (201+)			13.863***			10.300***
			(1.685)			(1.443)
AEA signals (2)			2.728**			1.293
			(1.148)			(1.061)
Constant	16.384***	33.952***	6.969	10.924***	23.749***	3.205
	(0.802)	(4.957)	(5.328)	(0.732)	(4.530)	(4.968)
Observations	417	403	394	417	403	394
Sample	Post-market	Post-market	Post-market	Post-market	Post-market	Post-market

Notes: This table presents the intent-to-treat (ITT) effect of being quote-tweeted by established economists on Twitter on the number of interviews received by job market candidates. Columns 1-3 show the effect for all jobs, while columns 4-6 focus on tenure-track positions. All specifications include a dummy variable indicating whether an individual is classified as belonging to underrepresented groups (URG) to account for the varying probability of treatment assignment. Columns 2 and 5 control for participants' personal background characteristics, including age, parental income class (on a 0-4 scale, coded as follows: 4 for "High income / wealthy", 3 for "Upper middle class", 2 for "Middle class", 1 for "Low income", and 0 for "In poverty"), US citizenship status, and Twitter account ownership. Columns 3 and 6 additionally control for participants' academic background, including graduating from a top 30 PhD institution (in or outside the US), being on the job market for the first time, predoctoral and postdoctoral experience, whether participants have at least one publication or paper under revision, the number of applications submitted (in ranges), and whether two AEA signals were sent. The post-market sample includes 417 participants who reported their job market outcomes. OLS estimates with robust standard errors in parentheses. Significance levels: * $p < 0.10$, ** $p < 0.05$, *** $p < 0.01$.

Table S11. Number of Flyouts (ITT)

**Dependent variable:**	**Number of Flyouts**					
	All Jobs			Tenure-Track Jobs		
	(1)	(2)	(3)	(4)	(5)	(6)
Treatment group	0.775	1.272**	1.052**	0.627	0.876*	0.684
	(0.527)	(0.531)	(0.502)	(0.462)	(0.470)	(0.460)
URG	0.440	0.363	0.355	0.369	0.173	0.267
	(0.533)	(0.543)	(0.536)	(0.462)	(0.469)	(0.475)
Age		-0.264***	-0.125**		-0.169***	-0.095*
		(0.058)	(0.059)		(0.051)	(0.051)
Parental income class		0.726**	0.479		0.176	0.059
		(0.315)	(0.324)		(0.265)	(0.273)
US citizen		0.535	0.425		-0.881*	-1.054**
		(0.647)	(0.622)		(0.453)	(0.454)
Twitter account		0.596	0.621		0.437	0.554
		(0.561)	(0.540)		(0.439)	(0.428)
Top 30 PhD institution			2.239***			2.124***
			(0.498)			(0.429)
First-time JMC			1.853***			1.034**
			(0.497)			(0.451)
Predoc			0.579			0.029
			(0.622)			(0.492)
Postdoc			-0.955**			-0.290
			(0.471)			(0.429)
Publication or R&R (1+)			0.973**			1.098***
			(0.490)			(0.414)
# applications (51-100)			2.139***			1.557***
			(0.648)			(0.600)
# applications (101-150)			3.223***			2.330***
			(0.682)			(0.625)
# applications (151-200)			3.015***			1.921***
			(0.738)			(0.652)
# applications (201+)			2.815***			1.570***
			(0.663)			(0.544)
AEA signals (2)			0.519			0.470
			(0.511)			(0.459)
Constant	5.334***	11.262***	1.466	3.322***	8.024***	1.475
	(0.330)	(2.137)	(2.429)	(0.275)	(1.865)	(2.026)
Observations	417	403	394	417	403	394
Sample	Post-market	Post-market	Post-market	Post-market	Post-market	Post-market

Notes: This table presents the intent-to-treat (ITT) effect of being quote-tweeted by established economists on Twitter on the number of flyouts received by job market candidates. For a description of the variables, see the table notes in Appendix Table S10. OLS estimates with robust standard errors in parentheses. Significance levels: * $p < 0.10$, ** $p < 0.05$, *** $p < 0.01$.

Table S12. Number of Offers (ITT)

**Dependent variable:**	Number of Offers					
	All Jobs			Tenure-Track Jobs		
	(1)	(2)	(3)	(4)	(5)	(6)
Treatment group	0.291<br>(0.234)	0.497**<br>(0.242)	0.408*<br>(0.234)	0.156<br>(0.196)	0.264<br>(0.203)	0.186<br>(0.205)
URG	0.162<br>(0.236)	0.121<br>(0.241)	0.067<br>(0.235)	0.299<br>(0.200)	0.216<br>(0.203)	0.245<br>(0.201)
Age		-0.156***<br>(0.025)	-0.100***<br>(0.028)		-0.088***<br>(0.022)	-0.066***<br>(0.022)
Parental income class		0.209<br>(0.159)	0.128<br>(0.148)		-0.072<br>(0.147)	-0.094<br>(0.137)
US citizen		-0.329<br>(0.261)	-0.444*<br>(0.257)		-0.746***<br>(0.188)	-0.866***<br>(0.194)
Twitter account		0.209<br>(0.263)	0.241<br>(0.261)		-0.031<br>(0.218)	0.029<br>(0.225)
Top 30 PhD institution			0.812***<br>(0.231)			0.811***<br>(0.206)
First-time JMC			0.766***<br>(0.277)			0.258<br>(0.247)
Predoc			0.253<br>(0.341)			-0.118<br>(0.254)
Postdoc			-0.492*<br>(0.268)			-0.117<br>(0.237)
Publication or R&R (1+)			0.604**<br>(0.246)			0.617***<br>(0.214)
# applications (51-100)			1.158***<br>(0.317)			0.694**<br>(0.293)
# applications (101-150)			1.233***<br>(0.337)			0.813***<br>(0.307)
# applications (151-200)			1.187***<br>(0.395)			0.678*<br>(0.355)
# applications (201+)			1.184***<br>(0.326)			0.632**<br>(0.284)
AEA signals (2)			0.473*<br>(0.253)			0.353<br>(0.224)
Constant	2.930***<br>(0.175)	7.215***<br>(1.012)	2.943**<br>(1.175)	1.468***<br>(0.142)	4.540***<br>(0.905)	2.104**<br>(0.962)
Observations	417	403	394	417	403	394
Sample	Post-market	Post-market	Post-market	Post-market	Post-market	Post-market

Notes: This table presents the intent-to-treat (ITT) effect of being quote-tweeted by established economists on Twitter on the number of offers received by job market candidates. For a description of the variables, see the table notes in Appendix Table S10. OLS estimates with robust standard errors in parentheses. Significance levels: * $p < 0.10$, ** $p < 0.05$, *** $p < 0.01$.

Table S13. Lee Bounds on Treatment Effects for Interviews, Flyouts, and Job Offers (ITT)

Dependent variable:	Number of Interviews, Flyouts, Job Offers					
	Interviews		Flyouts		Job Offers	
	Upper Bound	Lower Bound	Upper Bound	Lower Bound	Upper Bound	Lower Bound
	(1)	(2)	(3)	(4)	(5)	(6)
Treatment group	1.657	-1.021	1.289**	-0.061	0.569**	0.030
	(1.155)	(0.951)	(0.514)	(0.394)	(0.239)	(0.209)
URG	0.964	0.517	0.220	0.477	-0.023	-0.125
	(1.169)	(0.977)	(0.553)	(0.418)	(0.237)	(0.219)
Age	-0.301*	-0.157	-0.150**	-0.091*	-0.107***	-0.076***
	(0.158)	(0.115)	(0.073)	(0.054)	(0.032)	(0.025)
Parental income class	-0.746	-1.125**	0.401	0.170	0.090	0.110
	(0.728)	(0.518)	(0.326)	(0.242)	(0.152)	(0.117)
US citizen	1.560	1.239	0.309	0.352	-0.463*	-0.321
	(1.326)	(1.112)	(0.628)	(0.505)	(0.258)	(0.235)
Twitter account	2.452**	2.291**	0.486	0.775*	0.211	0.265
	(1.152)	(1.036)	(0.563)	(0.449)	(0.270)	(0.235)
Top 30 PhD institution	5.902***	5.461***	2.064***	2.292***	0.705***	0.726***
	(1.144)	(0.987)	(0.499)	(0.425)	(0.234)	(0.208)
First-time JMC	2.502**	2.696**	2.093***	1.785***	0.727**	0.806***
	(1.221)	(1.108)	(0.519)	(0.463)	(0.289)	(0.271)
Predoc	0.104	-0.203	0.599	0.570	0.263	0.016
	(1.332)	(1.114)	(0.636)	(0.544)	(0.349)	(0.293)
Postdoc	-4.727***	-3.803***	-0.758	-0.745*	-0.499*	-0.310
	(1.106)	(1.007)	(0.475)	(0.447)	(0.281)	(0.256)
Publication or R&R (1+)	3.530***	3.717***	1.024**	1.270***	0.668***	0.353
	(1.134)	(0.947)	(0.497)	(0.410)	(0.246)	(0.218)
# applications (51-100)	5.038***	6.497***	2.038***	2.259***	0.997***	1.172***
	(1.500)	(1.394)	(0.650)	(0.591)	(0.322)	(0.307)
# applications (101-150)	6.465***	8.029***	3.075***	3.183***	1.103***	1.158***
	(1.467)	(1.339)	(0.698)	(0.625)	(0.349)	(0.320)
# applications (151-200)	9.407***	9.807***	2.887***	2.782***	1.102***	0.915***
	(1.777)	(1.643)	(0.739)	(0.632)	(0.398)	(0.336)
# applications (201+)	12.893***	11.427***	2.684***	2.406***	1.042***	1.037***
	(1.708)	(1.468)	(0.683)	(0.557)	(0.341)	(0.301)
AEA signals (2)	2.891**	2.257**	0.734	0.294	0.463*	0.320
	(1.204)	(1.086)	(0.542)	(0.459)	(0.262)	(0.241)
Constant	9.322	5.976	2.375	1.120	3.467***	2.632**
	(5.962)	(4.691)	(2.779)	(2.217)	(1.284)	(1.042)
Observations	380	380	380	380	380	380

OLS estimates with robust standard errors in parentheses. Significance levels: * $p < 0.10$, ** $p < 0.05$, *** $p < 0.01$.

Table S14. Number of Interviews (ITT) — Heterogeneous Effects by URG

**Dependent variable:**	**Number of Interviews**					
	All Jobs			Tenure-Track Jobs		
	(1)	(2)	(3)	(4)	(5)	(6)
Treatment group	1.325	1.896	1.175	1.087	1.263	0.761
	(1.664)	(1.719)	(1.515)	(1.524)	(1.578)	(1.439)
URG	1.110	0.982	0.720	-0.020	-0.406	-0.255
	(1.672)	(1.690)	(1.584)	(1.403)	(1.443)	(1.408)
Treatment group × URG	-0.018	0.232	0.104	0.642	1.060	0.720
	(2.530)	(2.502)	(2.231)	(2.154)	(2.167)	(1.972)
Age		-0.669***	-0.268**		-0.429***	-0.167
		(0.139)	(0.130)		(0.128)	(0.124)
Parental income class		0.199	-0.530		-0.387	-0.844
		(0.756)	(0.702)		(0.638)	(0.600)
US citizen		2.063	1.541		-0.561	-1.003
		(1.481)	(1.310)		(1.213)	(1.138)
Twitter account		2.936**	2.864**		1.741	1.899*
		(1.241)	(1.117)		(1.068)	(0.972)
Top 30 PhD institution			5.817***			6.014***
			(1.132)			(1.025)
First-time JMC			2.514**			2.052*
			(1.172)			(1.167)
Predoc			0.432			-0.129
			(1.327)			(1.156)
Postdoc			-4.240***			-2.223**
			(1.046)			(1.022)
Publication or R&R (1+)			3.419***			3.845***
			(1.125)			(1.000)
# applications (51-100)			5.902***			4.496***
			(1.495)			(1.420)
# applications (101-150)			7.321***			6.054***
			(1.418)			(1.332)
# applications (151-200)			9.926***			7.077***
			(1.802)			(1.622)
# applications (201+)			13.862***			10.291***
			(1.690)			(1.441)
AEA signals (2)			2.729**			1.302
			(1.149)			(1.062)
Constant	16.382***	33.996***	6.979	11.020***	23.951***	3.277
	(0.847)	(4.977)	(5.331)	(0.787)	(4.563)	(4.962)
Observations	417	403	394	417	403	394
Sample	Post-market	Post-market	Post-market	Post-market	Post-market	Post-market

Notes: This table presents the heterogeneous intent-to-treat (ITT) effect, by the URG group, of being quote-tweeted by established economists on Twitter on the number of interviews received by job market candidates. For a description of the variables, see the table notes in Appendix Table S10. OLS estimates with robust standard errors in parentheses. Significance levels: * $p < 0.10$, ** $p < 0.05$, *** $p < 0.01$.

Table S15. Number of Flyouts (ITT) — Heterogeneous Effects by URG

**Dependent variable:**	**Number of Flyouts**					
	All Jobs			Tenure-Track Jobs		
	(1)	(2)	(3)	(4)	(5)	(6)
Treatment group	1.130	1.521**	1.213*	0.923	1.047	0.815
	(0.713)	(0.741)	(0.720)	(0.649)	(0.676)	(0.671)
URG	0.848	0.649	0.542	0.711	0.370	0.419
	(0.697)	(0.668)	(0.663)	(0.612)	(0.594)	(0.607)
Treatment group × URG	-0.784	-0.544	-0.352	-0.656	-0.375	-0.286
	(1.059)	(1.016)	(0.951)	(0.920)	(0.903)	(0.871)
Age		-0.264***	-0.125**		-0.169***	-0.095*
		(0.058)	(0.059)		(0.051)	(0.051)
Parental income class		0.730**	0.482		0.179	0.061
		(0.316)	(0.325)		(0.266)	(0.275)
US citizen		0.548	0.435		-0.872*	-1.046**
		(0.645)	(0.621)		(0.452)	(0.452)
Twitter account		0.591	0.616		0.434	0.550
		(0.562)	(0.540)		(0.440)	(0.428)
Top 30 PhD institution			2.242***			2.127***
			(0.498)			(0.429)
First-time JMC			1.847***			1.029**
			(0.503)			(0.456)
Predoc			0.568			0.020
			(0.625)			(0.494)
Postdoc			-0.947**			-0.284
			(0.474)			(0.432)
Publication or R&R (1+)			0.976**			1.100***
			(0.489)			(0.412)
# applications (51-100)			2.128***			1.548**
			(0.656)			(0.607)
# applications (101-150)			3.216***			2.324***
			(0.682)			(0.624)
# applications (151-200)			2.999***			1.908***
			(0.737)			(0.656)
# applications (201+)			2.819***			1.574***
			(0.665)			(0.547)
AEA signals (2)			0.515			0.466
			(0.511)			(0.457)
Constant	5.217***	11.158***	1.431	3.224***	7.953***	1.446
	(0.346)	(2.133)	(2.427)	(0.284)	(1.862)	(2.022)
Observations	417	403	394	417	403	394
Sample	Post-market	Post-market	Post-market	Post-market	Post-market	Post-market

Notes: This table presents the heterogeneous intent-to-treat (ITT) effect, by the URG group, of being quote-tweeted by established economists on Twitter on the number of flyouts received by job market candidates. For a description of the variables, see the table notes in Appendix Table S10. OLS estimates with robust standard errors in parentheses. Significance levels: * $p < 0.10$, ** $p < 0.05$, *** $p < 0.01$.

Table S16. Number of Offers (ITT) — Heterogeneous Effects by URG

**Dependent variable:**	Number of Offers					
	All Jobs			Tenure-Track Jobs		
	(1)	(2)	(3)	(4)	(5)	(6)
Treatment group	0.180<br>(0.312)	0.307<br>(0.325)	0.161<br>(0.316)	0.020<br>(0.257)	0.065<br>(0.263)	-0.035<br>(0.265)
URG	0.033<br>(0.326)	-0.098<br>(0.332)	-0.220<br>(0.335)	0.143<br>(0.268)	-0.014<br>(0.272)	-0.012<br>(0.283)
Treatment group × URG	0.247<br>(0.472)	0.416<br>(0.471)	0.540<br>(0.462)	0.301<br>(0.397)	0.437<br>(0.398)	0.482<br>(0.403)
Age		-0.156***<br>(0.026)	-0.099***<br>(0.027)		-0.088***<br>(0.022)	-0.065***<br>(0.022)
Parental income class		0.206<br>(0.159)	0.124<br>(0.148)		-0.075<br>(0.147)	-0.097<br>(0.137)
US citizen		-0.339<br>(0.261)	-0.459*<br>(0.257)		-0.757***<br>(0.188)	-0.880***<br>(0.194)
Twitter account		0.213<br>(0.264)	0.248<br>(0.261)		-0.027<br>(0.219)	0.035<br>(0.226)
Top 30 PhD institution			0.806***<br>(0.231)			0.805***<br>(0.206)
First-time JMC			0.776***<br>(0.277)			0.266<br>(0.248)
Predoc			0.271<br>(0.345)			-0.103<br>(0.256)
Postdoc			-0.504*<br>(0.269)			-0.127<br>(0.238)
Publication or R&R (1+)			0.599**<br>(0.246)			0.613***<br>(0.214)
# applications (51-100)			1.175***<br>(0.314)			0.709**<br>(0.290)
# applications (101-150)			1.243***<br>(0.337)			0.823***<br>(0.308)
# applications (151-200)			1.212***<br>(0.396)			0.700*<br>(0.357)
# applications (201+)			1.178***<br>(0.325)			0.626**<br>(0.283)
AEA signals (2)			0.480*<br>(0.254)			0.360<br>(0.227)
Constant	2.967***<br>(0.191)	7.294***<br>(1.021)	2.997**<br>(1.170)	1.513***<br>(0.154)	4.623***<br>(0.921)	2.153**<br>(0.959)
Observations	417	403	394	417	403	394
Sample	Post-market	Post-market	Post-market	Post-market	Post-market	Post-market

Notes: This table presents the heterogeneous intent-to-treat (ITT) effect, by the URG group, of being quote-tweeted by established economists on Twitter on the number of offers received by job market candidates. For a description of the variables, see the table notes in Appendix Table S10. OLS estimates with robust standard errors in parentheses. Significance levels: * $p < 0.10$, ** $p < 0.05$, *** $p < 0.01$.

Table S17. Number of Interviews (ITT) — Heterogeneous Effects by Female

**Dependent variable:**	**Number of Interviews**					
	All Jobs			Tenure-Track Jobs		
	(1)	(2)	(3)	(4)	(5)	(6)
Treatment group	0.480<br>(1.515)	0.979<br>(1.570)	1.262<br>(1.378)	0.696<br>(1.315)	0.833<br>(1.371)	1.082<br>(1.233)
URG	-1.129<br>(1.901)	-0.188<br>(1.855)	-1.731<br>(1.610)	-1.461<br>(1.457)	-1.064<br>(1.468)	-2.040<br>(1.271)
Female	1.794<br>(2.530)	0.205<br>(2.467)	3.866*<br>(2.102)	1.405<br>(2.055)	0.274<br>(2.034)	3.274*<br>(1.837)
Treatment group × female	2.623<br>(2.702)	3.092<br>(2.657)	-0.363<br>(2.233)	2.134<br>(2.313)	2.757<br>(2.323)	-0.182<br>(2.044)
Age		-0.666***<br>(0.140)	-0.248*<br>(0.131)		-0.425***<br>(0.129)	-0.150<br>(0.124)
Parental income class		0.081<br>(0.742)	-0.652<br>(0.693)		-0.489<br>(0.630)	-0.947<br>(0.595)
US citizen		1.989<br>(1.478)	1.523<br>(1.282)		-0.607<br>(1.202)	-1.003<br>(1.112)
Twitter account		2.874**<br>(1.241)	2.636**<br>(1.113)		1.672<br>(1.059)	1.695*<br>(0.968)
Top 30 PhD institution			5.913***<br>(1.136)			6.101***<br>(1.026)
First-time JMC			2.527**<br>(1.191)			2.054*<br>(1.176)
Predoc			0.202<br>(1.326)			-0.349<br>(1.151)
Postdoc			-4.378***<br>(1.096)			-2.331**<br>(1.062)
Publication or R&R (1+)			3.433***<br>(1.112)			3.858***<br>(0.986)
# applications (51-100)			5.940***<br>(1.494)			4.507***<br>(1.424)
# applications (101-150)			7.703***<br>(1.419)			6.375***<br>(1.332)
# applications (151-200)			10.265***<br>(1.789)			7.340***<br>(1.610)
# applications (201+)			14.183***<br>(1.679)			10.569***<br>(1.456)
AEA signals (2)			2.572**<br>(1.131)			1.155<br>(1.054)
Constant	16.661***<br>(0.832)	34.522***<br>(4.971)	6.615<br>(5.338)	11.149***<br>(0.758)	24.261***<br>(4.560)	2.921<br>(4.975)
Treatment × female + Treatment = 0<br>(p-values from t-tests)	0.166	0.064	0.620	0.138	0.061	0.594
Observations	417	403	394	417	403	394
Sample	Post-market	Post-market	Post-market	Post-market	Post-market	Post-market

Notes: This table presents the heterogeneous intent-to-treat (ITT) effect, by gender, of being quote-tweeted by established economists on Twitter on the number of interviews received by job market candidates. For a description of the variables, see the table notes in Appendix Table S10. OLS estimates with robust standard errors in parentheses. Significance levels: * $p < 0.10$, ** $p < 0.05$, *** $p < 0.01$.

Table S18. Number of Flyouts (ITT) — Heterogeneous Effects by Female

**Dependent variable:**	**Number of Flyouts**					
	All Jobs			Tenure-Track Jobs		
	(1)	(2)	(3)	(4)	(5)	(6)
Treatment group	0.891	1.275**	1.231**	0.773	0.903	0.861
	(0.609)	(0.634)	(0.599)	(0.552)	(0.578)	(0.563)
URG	-1.294*	-0.864	-1.279**	-0.691	-0.580	-0.757
	(0.671)	(0.669)	(0.587)	(0.583)	(0.584)	(0.549)
Female	2.649**	1.801*	2.776***	1.743*	1.147	1.847**
	(1.026)	(0.959)	(0.845)	(0.910)	(0.863)	(0.801)
Treatment group × female	-0.367	-0.087	-0.719	-0.462	-0.132	-0.652
	(1.172)	(1.099)	(0.991)	(1.001)	(0.972)	(0.899)
Age		-0.259***	-0.112*		-0.166***	-0.087*
		(0.058)	(0.059)		(0.051)	(0.051)
Parental income class		0.669**	0.405		0.143	0.015
		(0.310)	(0.317)		(0.266)	(0.274)
US citizen		0.513	0.419		-0.893**	-1.055**
		(0.634)	(0.602)		(0.446)	(0.444)
Twitter account		0.503	0.467		0.379	0.454
		(0.564)	(0.538)		(0.443)	(0.430)
Top 30 PhD institution			2.311***			2.174***
			(0.496)			(0.429)
First-time JMC			1.855***			1.032**
			(0.507)			(0.456)
Predoc			0.430			-0.065
			(0.625)			(0.491)
Postdoc			-1.033**			-0.335
			(0.482)			(0.437)
Publication or R&R (1+)			0.997**			1.120***
			(0.480)			(0.406)
# applications (51-100)			2.174***			1.582***
			(0.652)			(0.606)
# applications (101-150)			3.469***			2.482***
			(0.678)			(0.622)
# applications (151-200)			3.243***			2.065***
			(0.744)			(0.657)
# applications (201+)			3.051***			1.730***
			(0.667)			(0.557)
AEA signals (2)			0.426			0.415
			(0.498)			(0.453)
Constant	5.296***	11.310***	1.154	3.273***	8.041***	1.245
	(0.333)	(2.124)	(2.429)	(0.275)	(1.864)	(2.018)
Treatment × female + Treatment = 0 (p-values from t-tests)	0.601	0.197	0.531	0.709	0.330	0.774
Observations	417	403	394	417	403	394
Sample	Post-market	Post-market	Post-market	Post-market	Post-market	Post-market

Notes: This table presents the heterogeneous intent-to-treat (ITT) effect, by gender, of being quote-tweeted by established economists on Twitter on the number of flyouts received by job market candidates. For a description of the variables, see the table notes in Appendix Table S10. OLS estimates with robust standard errors in parentheses. Significance levels: * $p < 0.10$, ** $p < 0.05$, *** $p < 0.01$.

Table S19. Number of Offers (ITT) — Heterogeneous Effects by Female

**Dependent variable:**	**Number of Offers**					
	All Jobs			Tenure-Track Jobs		
	(1)	(2)	(3)	(4)	(5)	(6)
Treatment group	0.033 (0.279)	0.156 (0.287)	0.138 (0.273)	-0.024 (0.219)	0.013 (0.225)	-0.003 (0.224)
URG	-0.591* (0.335)	-0.487 (0.326)	-0.673** (0.304)	-0.262 (0.226)	-0.302 (0.229)	-0.362 (0.224)
Female	0.646 (0.464)	0.328 (0.464)	0.671 (0.434)	0.501 (0.365)	0.345 (0.364)	0.605* (0.356)
Treatment group × female	0.809 (0.506)	1.018** (0.504)	0.777 (0.477)	0.564 (0.452)	0.745 (0.454)	0.535 (0.447)
Age		-0.154*** (0.025)	-0.094*** (0.027)		-0.086*** (0.021)	-0.061*** (0.021)
Parental income class		0.161 (0.152)	0.079 (0.141)		-0.110 (0.144)	-0.132 (0.135)
US citizen		-0.359 (0.254)	-0.464* (0.248)		-0.769*** (0.184)	-0.881*** (0.190)
Twitter account		0.175 (0.264)	0.187 (0.259)		-0.061 (0.218)	-0.017 (0.223)
Top 30 PhD institution			0.820*** (0.230)			0.819*** (0.206)
First-time JMC			0.784*** (0.283)			0.271 (0.251)
Predoc			0.188 (0.346)			-0.172 (0.251)
Postdoc			-0.556** (0.273)			-0.166 (0.241)
Publication or R&R (1+)			0.577** (0.240)			0.598*** (0.209)
# applications (51-100)			1.156*** (0.314)			0.694** (0.291)
# applications (101-150)			1.356*** (0.335)			0.913*** (0.307)
# applications (151-200)			1.282*** (0.391)			0.757** (0.352)
# applications (201+)			1.227*** (0.329)			0.673** (0.289)
AEA signals (2)			0.411* (0.248)			0.304 (0.223)
Constant	3.016*** (0.184)	7.412*** (0.997)	2.988** (1.165)	1.528*** (0.146)	4.688*** (0.904)	2.124** (0.952)
Treatment × female + Treatment = 0 (p-values from t-tests)	0.047	0.006	0.024	0.172	0.058	0.179
Observations	417	403	394	417	403	394
Sample	Post-market	Post-market	Post-market	Post-market	Post-market	Post-market

Notes: This table presents the heterogeneous intent-to-treat (ITT) effect, by gender, of being quote-tweeted by established economists on Twitter on the number of offers received by job market candidates. For a description of the variables, see the table notes in Appendix Table S10. OLS estimates with robust standard errors in parentheses. Significance levels: * $p < 0.10$, ** $p < 0.05$, *** $p < 0.01$.

Table S20. Number of Interviews (LATE)

**Dependent variable:**	**Number of Interviews**					
	All Jobs			Tenure-Track Jobs		
	(1)	(2)	(3)	(4)	(5)	(6)
Treatment	1.481	2.241	1.376	1.548	1.955	1.228
	(1.403)	(1.437)	(1.248)	(1.210)	(1.252)	(1.128)
URG	1.130	1.172	0.812	0.346	0.210	0.161
	(1.258)	(1.267)	(1.106)	(1.071)	(1.099)	(1.006)
Age		-0.670***	-0.269**		-0.428***	-0.169
		(0.138)	(0.128)		(0.127)	(0.121)
Parental income class		0.194	-0.539		-0.385	-0.848
		(0.751)	(0.684)		(0.633)	(0.584)
US citizen		2.084	1.553		-0.522	-0.974
		(1.478)	(1.289)		(1.206)	(1.118)
Twitter account		2.865**	2.813**		1.671	1.845*
		(1.238)	(1.094)		(1.058)	(0.949)
Top 30 PhD institution			5.805***			6.010***
			(1.111)			(1.004)
First-time JMC			2.556**			2.078*
			(1.139)			(1.134)
Predoc			0.485			-0.103
			(1.292)			(1.126)
Postdoc			-4.246***			-2.214**
			(1.019)			(1.001)
Publication or R&R (1+)			3.433***			3.863***
			(1.097)			(0.977)
# applications (51-100)			5.798***			4.384***
			(1.474)			(1.406)
# applications (101-150)			7.228***			5.959***
			(1.408)			(1.318)
# applications (151-200)			9.796***			6.933***
			(1.760)			(1.588)
# applications (201+)			13.767***			10.215***
			(1.657)			(1.415)
AEA signals (2)			2.758**			1.319
			(1.126)			(1.041)
Constant	16.376***	34.015***	7.081	10.915***	23.805***	3.305
	(0.803)	(4.916)	(5.233)	(0.732)	(4.490)	(4.878)
First stage instrument:						
Treatment assignment	0.889	0.893	0.888	0.889	0.893	0.888
Standard error	0.023	0.022	0.024	0.023	0.022	0.024
F-statistics	1505.779	1584.249	1414.141	1505.779	1584.249	1414.141
Observations	417	403	394	417	403	394
Sample	Post-market	Post-market	Post-market	Post-market	Post-market	Post-market

Notes: This table presents the local average treatment effect (LATE) of being quote-tweeted by established economists on Twitter on the number of interviews received by job market candidates. For a description of the variables, see the table notes in Appendix Table S10. 2SLS estimates with robust standard errors in parentheses. Significance levels: * $p < 0.10$, ** $p < 0.05$, *** $p < 0.01$.

Table S21. Number of Flyouts (LATE)

**Dependent variable:**	**Number of Flyouts**					
	All Jobs			Tenure-Track Jobs		
	(1)	(2)	(3)	(4)	(5)	(6)
Treatment	0.872	1.424**	1.184**	0.705	0.981*	0.770
	(0.590)	(0.590)	(0.554)	(0.516)	(0.521)	(0.506)
URG	0.457	0.407	0.386	0.383	0.203	0.287
	(0.526)	(0.535)	(0.522)	(0.455)	(0.457)	(0.457)
Age		-0.264***	-0.126**		-0.170***	-0.096*
		(0.057)	(0.058)		(0.051)	(0.050)
Parental income class		0.722**	0.470		0.173	0.053
		(0.313)	(0.317)		(0.262)	(0.267)
US citizen		0.544	0.433		-0.875*	-1.049**
		(0.651)	(0.619)		(0.452)	(0.447)
Twitter account		0.552	0.578		0.407	0.526
		(0.565)	(0.535)		(0.436)	(0.418)
Top 30 PhD institution			2.226***			2.116***
			(0.488)			(0.420)
First-time JMC			1.890***			1.058**
			(0.483)			(0.438)
Predoc			0.628			0.061
			(0.611)			(0.482)
Postdoc			-0.962**			-0.295
			(0.459)			(0.417)
Publication or R&R (1+)			0.983**			1.105***
			(0.479)			(0.403)
# applications (51-100)			2.052***			1.501**
			(0.639)			(0.593)
# applications (101-150)			3.145***			2.279***
			(0.673)			(0.616)
# applications (151-200)			2.908***			1.851***
			(0.725)			(0.640)
# applications (201+)			2.732***			1.516***
			(0.652)			(0.534)
AEA signals (2)			0.545			0.486
			(0.503)			(0.451)
Constant	5.329***	11.302***	1.563	3.318***	8.052***	1.538
	(0.330)	(2.116)	(2.385)	(0.275)	(1.847)	(1.989)
First stage instrument:						
Treatment assignment	0.889	0.893	0.888	0.889	0.893	0.888
Standard error	0.023	0.022	0.024	0.023	0.022	0.024
F-statistics	1505.779	1584.249	1414.141	1505.779	1584.249	1414.141
Observations	417	403	394	417	403	394
Sample	Post-market	Post-market	Post-market	Post-market	Post-market	Post-market

Notes: This table presents the local average treatment effect (LATE) of being quote-tweeted by established economists on Twitter on the number of flyouts received by job market candidates. For a description of the variables, see the table notes in Appendix Table S10. 2SLS estimates with robust standard errors in parentheses. Significance levels: * $p < 0.10$, ** $p < 0.05$, *** $p < 0.01$.

Table S22. Number of Offers (LATE)

**Dependent variable:**	**Number of Offers**					
	All Jobs			Tenure-Track Jobs		
	(1)	(2)	(3)	(4)	(5)	(6)
Treatment	0.327	0.556**	0.460*	0.176	0.296	0.209
	(0.262)	(0.269)	(0.258)	(0.220)	(0.225)	(0.226)
URG	0.168	0.138	0.080	0.303	0.225	0.250
	(0.235)	(0.239)	(0.229)	(0.198)	(0.201)	(0.196)
Age		-0.156***	-0.100***		-0.088***	-0.066***
		(0.025)	(0.027)		(0.021)	(0.021)
Parental income class		0.207	0.124		-0.073	-0.095
		(0.158)	(0.145)		(0.146)	(0.134)
US citizen		-0.325	-0.441*		-0.744***	-0.865***
		(0.262)	(0.254)		(0.188)	(0.191)
Twitter account		0.192	0.225		-0.040	0.021
		(0.263)	(0.256)		(0.216)	(0.220)
Top 30 PhD institution			0.808***			0.808***
			(0.227)			(0.202)
First-time JMC			0.781***			0.265
			(0.269)			(0.240)
Predoc			0.272			-0.110
			(0.335)			(0.251)
Postdoc			-0.495*			-0.118
			(0.261)			(0.231)
Publication or R&R (1+)			0.608**			0.619***
			(0.240)			(0.209)
# applications (51-100)			1.124***			0.678**
			(0.311)			(0.287)
# applications (101-150)			1.202***			0.799***
			(0.333)			(0.301)
# applications (151-200)			1.146***			0.659*
			(0.386)			(0.344)
# applications (201+)			1.152***			0.617**
			(0.323)			(0.279)
AEA signals (2)			0.483*			0.358
			(0.248)			(0.219)
Constant	2.928***	7.231***	2.980***	1.467***	4.548***	2.121**
	(0.175)	(1.001)	(1.153)	(0.142)	(0.895)	(0.941)
First stage instrument:						
Treatment assignment	0.889	0.893	0.888	0.889	0.893	0.888
Standard error	0.023	0.022	0.024	0.023	0.022	0.024
F-statistics	1505.779	1584.249	1414.141	1505.779	1584.249	1414.141
Observations	417	403	394	417	403	394
Sample	Post-market	Post-market	Post-market	Post-market	Post-market	Post-market

Notes: This table presents the local average treatment effect (LATE) of being quote-tweeted by established economists on Twitter on the number of offers received by job market candidates. For a description of the variables, see the table notes in Appendix Table S10. 2SLS estimates with robust standard errors in parentheses. Significance levels: * $p < 0.10$, ** $p < 0.05$, *** $p < 0.01$.

Table S23. Annual Pre-tax Salary (ITT and LATE)

**Dependent variable:**	**Salary (in USD 1,000)**					
	ITT			LATE		
	(1)	(2)	(3)	(4)	(5)	(6)
Treatment				8.041	9.802	5.233
				(8.460)	(8.414)	(7.769)
Treatment group	7.282	8.872	4.680			
	(7.706)	(7.716)	(7.180)			
URG	-6.334	-2.596	-1.629	-6.065	-2.236	-1.463
	(7.873)	(7.755)	(8.182)	(7.754)	(7.575)	(7.845)
Age		-0.060	0.216		-0.038	0.220
		(1.077)	(0.957)		(1.056)	(0.925)
Parental income class		4.206	0.880		4.157	0.846
		(5.313)	(4.598)		(5.236)	(4.450)
US citizen		24.721***	19.825***		24.788***	19.881***
		(7.751)	(7.278)		(7.643)	(7.061)
Twitter account		-0.465	7.302		-0.744	7.165
		(7.941)	(8.399)		(7.822)	(8.109)
Top 30 PhD institution			47.925***			47.996***
			(9.250)			(8.961)
First-time JMC			-8.491			-8.658
			(10.662)			(10.333)
Predoc			4.439			4.391
			(8.563)			(8.277)
Postdoc			-21.968**			-22.095**
			(9.430)			(9.135)
Publication or R&R (1+)			10.511			10.523
			(8.005)			(7.744)
# applications (51-100)			36.941**			36.359**
			(15.846)			(15.331)
# applications (101-150)			16.047*			15.569*
			(9.203)			(9.055)
# applications (151-200)			20.840**			20.399**
			(10.371)			(10.080)
# applications (201+)			21.140**			20.747**
			(9.461)			(9.208)
AEA signals (2)			3.814			3.764
			(10.401)			(10.080)
Constant	107.320***	91.198**	45.769	107.248***	90.729**	46.320
	(4.767)	(39.918)	(40.193)	(4.774)	(39.258)	(39.068)
First stage instrument:						
Treatment				0.906	0.905	0.894
Standard error				0.026	0.025	0.028
F-statistics				1249.737	1266.604	1011.473
Observations	281	276	276	281	276	276
Sample	Post-market	Post-market	Post-market	Post-market	Post-market	Post-market

Notes: This table presents the intent-to-treat (ITT) effect and the local average treatment effect (LATE) of being quote-tweeted by established economists on Twitter on the pre-tax annual salary self-reported by job market candidates. For a description of the variables, see the table notes in Appendix Table S10. OLS and 2SLS estimates with robust standard errors in parentheses. Significance levels: * $p < 0.10$, ** $p < 0.05$, *** $p < 0.01$.

Table S24. Number of Views and Likes (ITT) — Median Split by Influencers' Twitter Followers

**Dependent variable:**	Number of Views and Likes					
	Views			Likes		
	(1)	(2)	(3)	(4)	(5)	(6)
Above-median follower influencer	4782.751***	4816.752***	4831.574***	13.125***	12.632***	12.692***
	(708.507)	(718.606)	(702.408)	(1.858)	(1.759)	(1.729)
Below-median follower influencer	1712.509***	1729.808***	1738.810***	9.525***	9.162***	9.084***
	(384.664)	(394.218)	(397.883)	(2.268)	(2.270)	(2.302)
URG	607.305	517.771	612.296	0.303	0.164	0.192
	(454.568)	(439.549)	(451.127)	(1.597)	(1.624)	(1.684)
Age		-11.800	-53.174		-0.180	-0.190
		(53.344)	(57.463)		(0.176)	(0.163)
Parental income class		202.281	202.631		-0.327	-0.565
		(235.869)	(241.836)		(0.981)	(0.990)
US citizen		-432.925	-571.222		-1.913	-2.125
		(409.602)	(410.666)		(1.419)	(1.387)
Twitter account		506.809	663.323*		1.533	1.877
		(334.391)	(356.453)		(1.505)	(1.502)
Top 30 PhD institution			364.825			1.705
			(469.524)			(1.576)
First-time JMC			-1901.851*			-2.292
			(1047.848)			(2.225)
Predoc			-9.562			1.242
			(511.718)			(1.863)
Postdoc			-642.025			-2.427
			(696.293)			(1.900)
Constant	729.626***	421.452	3171.883	3.763***	9.628	11.561*
	(204.303)	(1911.320)	(2016.190)	(0.853)	(6.594)	(6.143)
Above-median = Below-median (p-values from t-tests)	<0.001	<0.001	<0.001	0.172	0.184	0.166
Observations	417	403	403	417	403	403
Sample	Post-market	Post-market	Post-market	Post-market	Post-market	Post-market

Notes: This table presents the intent-to-treat (ITT) effect, by influencers' Twitter followers (median split), of being quote-tweeted by established economists on Twitter on the number of views and likes. 'Views' represents the number of views received by job market paper tweets (which naturally include views of quote-tweets), and 'Likes' is the sum of likes received by the job market paper tweets and the influencer quote-tweets. For a description of the variables, see the table notes in Appendix Table S6. OLS estimates with robust standard errors in parentheses. Significance levels: * $p < 0.10$, ** $p < 0.05$, *** $p < 0.01$.

Table S25. Number of Interviews (ITT) — Median Split by Influencers' Twitter Followers

**Dependent variable:**	**Number of Interviews**					
	All Jobs			Tenure-Track Jobs		
	(1)	(2)	(3)	(4)	(5)	(6)
Above-median follower influencer	-0.529	0.048	-0.277	-0.318	-0.063	-0.491
	(1.330)	(1.347)	(1.114)	(1.149)	(1.153)	(1.000)
Below-median follower influencer	3.663**	4.481**	3.090*	3.532**	4.043**	3.061**
	(1.848)	(1.870)	(1.658)	(1.543)	(1.611)	(1.464)
URG	0.970	0.949	0.669	0.195	0.007	0.017
	(1.266)	(1.272)	(1.136)	(1.080)	(1.110)	(1.037)
Age		-0.654***	-0.261**		-0.414***	-0.160
		(0.135)	(0.130)		(0.124)	(0.124)
Parental income class		0.233	-0.492		-0.349	-0.799
		(0.741)	(0.691)		(0.625)	(0.588)
US citizen		2.122	1.646		-0.486	-0.875
		(1.453)	(1.291)		(1.181)	(1.113)
Twitter account		3.316***	3.194***		2.085*	2.238**
		(1.272)	(1.144)		(1.091)	(0.989)
Top 30 PhD institution			5.790***			5.992***
			(1.128)			(1.017)
First-time JMC			2.476**			2.002*
			(1.210)			(1.196)
Predoc			0.266			-0.325
			(1.322)			(1.144)
Postdoc			-4.415***			-2.394**
			(1.092)			(1.062)
Publication or R&R (1+)			3.546***			3.984***
			(1.100)			(0.982)
# applications (51-100)			5.680***			4.243***
			(1.497)			(1.423)
# applications (101-150)			7.246***			5.964***
			(1.417)			(1.325)
# applications (151-200)			9.790***			6.906***
			(1.793)			(1.630)
# applications (201+)			13.768***			10.200***
			(1.681)			(1.441)
AEA signals (2)			2.366**			0.911
			(1.141)			(1.069)
Constant	16.422***	33.177***	6.836	10.958***	23.032***	3.064
	(0.802)	(4.842)	(5.316)	(0.733)	(4.415)	(4.945)
Above-median = Below-median (p-values from t-tests)	0.032	0.022	0.038	0.017	0.012	0.012
Observations	417	403	394	417	403	394
Sample	Post-market	Post-market	Post-market	Post-market	Post-market	Post-market

Notes: This table presents the intent-to-treat (ITT) effect, by influencers' Twitter followers (median split), of being quote-tweeted by established economists on Twitter on the number of interviews received by job market candidates. For a description of the variables, see the table notes in Appendix Table S10. OLS estimates with robust standard errors in parentheses. Significance levels: * $p < 0.10$, ** $p < 0.05$, *** $p < 0.01$.

Table S26. Number of Flyouts (ITT) — Median Split by Influencers' Twitter Followers

**Dependent variable:**	**Number of Flyouts**					
	All Jobs			Tenure-Track Jobs		
	(1)	(2)	(3)	(4)	(5)	(6)
Above-median follower influencer	0.137<br>(0.561)	0.619<br>(0.559)	0.552<br>(0.507)	-0.031<br>(0.471)	0.204<br>(0.474)	0.082<br>(0.449)
Below-median follower influencer	1.587**<br>(0.765)	2.102***<br>(0.758)	1.674**<br>(0.724)	1.462**<br>(0.655)	1.730***<br>(0.663)	1.434**<br>(0.642)
URG	0.395<br>(0.532)	0.311<br>(0.538)	0.319<br>(0.534)	0.322<br>(0.462)	0.120<br>(0.467)	0.224<br>(0.475)
Age		-0.259***<br>(0.058)	-0.122**<br>(0.060)		-0.164***<br>(0.051)	-0.092*<br>(0.051)
Parental income class		0.737**<br>(0.310)	0.492<br>(0.320)		0.187<br>(0.258)	0.074<br>(0.267)
US citizen		0.552<br>(0.642)	0.459<br>(0.620)		-0.863*<br>(0.449)	-1.013**<br>(0.448)
Twitter account		0.724<br>(0.563)	0.731<br>(0.541)		0.569<br>(0.445)	0.686<br>(0.433)
Top 30 PhD institution			2.229***<br>(0.495)			2.112***<br>(0.425)
First-time JMC			1.841***<br>(0.501)			1.020**<br>(0.452)
Predoc			0.525<br>(0.622)			-0.036<br>(0.491)
Postdoc			-1.014**<br>(0.484)			-0.362<br>(0.436)
Publication or R&R (1+)			1.014**<br>(0.483)			1.148***<br>(0.406)
# applications (51-100)			2.066***<br>(0.653)			1.469**<br>(0.601)
# applications (101-150)			3.199***<br>(0.686)			2.301***<br>(0.622)
# applications (151-200)			2.972***<br>(0.746)			1.868***<br>(0.656)
# applications (201+)			2.783***<br>(0.667)			1.532***<br>(0.544)
AEA signals (2)			0.399<br>(0.512)			0.324<br>(0.457)
Constant	5.347***<br>(0.330)	11.002***<br>(2.123)	1.422<br>(2.440)	3.335***<br>(0.275)	7.758***<br>(1.842)	1.422<br>(2.031)
Above median = Below median<br>(p-values from t-tests)	0.070	0.059	0.115	0.021	0.019	0.024
Observations	417	403	394	417	403	394
Sample	Post-market	Post-market	Post-market	Post-market	Post-market	Post-market

Notes: This table presents the intent-to-treat (ITT) effect, by influencers' Twitter followers (median split), of being quote-tweeted by established economists on Twitter on the number of flyouts received by job market candidates. For a description of the variables, see the table notes in Appendix Table S10. OLS estimates with robust standard errors in parentheses. Significance levels: * $p < 0.10$, ** $p < 0.05$, *** $p < 0.01$.

Table S27. Number of Offers (ITT) — Median Split by Influencers' Twitter Followers

**Dependent variable:**	**Number of Offers**					
	All Jobs			Tenure-Track Jobs		
	(1)	(2)	(3)	(4)	(5)	(6)
Above-median follower influencer	0.031<br>(0.262)	0.231<br>(0.268)	0.221<br>(0.254)	-0.126<br>(0.226)	-0.014<br>(0.229)	-0.065<br>(0.228)
Below-median follower influencer	0.622*<br>(0.329)	0.834**<br>(0.333)	0.642**<br>(0.323)	0.515*<br>(0.276)	0.617**<br>(0.285)	0.498*<br>(0.284)
URG	0.143<br>(0.235)	0.100<br>(0.239)	0.054<br>(0.235)	0.280<br>(0.197)	0.194<br>(0.201)	0.227<br>(0.200)
Age		-0.154***<br>(0.026)	-0.099***<br>(0.028)		-0.086***<br>(0.022)	-0.065***<br>(0.022)
Parental income class		0.214<br>(0.157)	0.132<br>(0.147)		-0.067<br>(0.145)	-0.087<br>(0.136)
US citizen		-0.322<br>(0.259)	-0.432*<br>(0.255)		-0.739***<br>(0.186)	-0.849***<br>(0.192)
Twitter account		0.261<br>(0.266)	0.283<br>(0.264)		0.023<br>(0.219)	0.084<br>(0.226)
Top 30 PhD institution			0.809***<br>(0.231)			0.806***<br>(0.205)
First-time JMC			0.762***<br>(0.283)			0.252<br>(0.252)
Predoc			0.233<br>(0.339)			-0.146<br>(0.254)
Postdoc			-0.514*<br>(0.276)			-0.146<br>(0.247)
Publication or R&R (1+)			0.620**<br>(0.247)			0.638***<br>(0.215)
# applications (51-100)			1.130***<br>(0.318)			0.657**<br>(0.295)
# applications (101-150)			1.224***<br>(0.337)			0.801***<br>(0.305)
# applications (151-200)			1.171***<br>(0.390)			0.657*<br>(0.350)
# applications (201+)			1.172***<br>(0.324)			0.616**<br>(0.281)
AEA signals (2)			0.428*<br>(0.256)			0.293<br>(0.225)
Constant	2.936***<br>(0.175)	7.110***<br>(1.018)	2.926**<br>(1.184)	1.474***<br>(0.141)	4.430***<br>(0.906)	2.082**<br>(0.972)
Above median = Below median<br>(p-values from t-tests)	0.097	0.089	0.208	0.038	0.046	0.065
Observations	417	403	394	417	403	394
Sample	Post-market	Post-market	Post-market	Post-market	Post-market	Post-market

Notes: This table presents the intent-to-treat (ITT) effect, by influencers' Twitter followers (median split), of being quote-tweeted by established economists on Twitter on the number of offers received by job market candidates. For a description of the variables, see the table notes in Appendix Table S10. OLS estimates with robust standard errors in parentheses. Significance levels: * $p < 0.10$, ** $p < 0.05$, *** $p < 0.01$.

Table S28. Number of Interviews (ITT) — Median Split by Quote-tweet Specificity

**Dependent variable:**	**Number of Interviews**					
	All Jobs			Tenure-Track Jobs		
	(1)	(2)	(3)	(4)	(5)	(6)
Tweet above median specificity	1.721	2.447	2.005	1.787	2.037	1.840
	(1.918)	(1.907)	(1.778)	(1.610)	(1.654)	(1.568)
Tweet below median specificity	1.066	1.602	0.528	1.411	1.706	0.720
	(1.448)	(1.466)	(1.215)	(1.270)	(1.303)	(1.110)
No tweet	1.427	2.758	2.509	0.011	1.071	0.825
	(3.359)	(3.468)	(2.637)	(2.309)	(2.401)	(2.005)
URG	1.082	1.065	0.716	0.336	0.169	0.120
	(1.271)	(1.286)	(1.137)	(1.091)	(1.125)	(1.043)
Age		-0.665***	-0.260**		-0.427***	-0.163
		(0.139)	(0.129)		(0.128)	(0.123)
Parental income class		0.230	-0.475		-0.371	-0.808
		(0.763)	(0.707)		(0.648)	(0.610)
US citizen		2.121	1.623		-0.507	-0.913
		(1.488)	(1.307)		(1.223)	(1.141)
Twitter account		2.930**	2.863**		1.696	1.839*
		(1.230)	(1.114)		(1.069)	(0.979)
Top 30 PhD institution			5.895***			6.066***
			(1.110)			(1.004)
First-time JMC			2.462**			2.045*
			(1.186)			(1.176)
Predoc			0.364			-0.145
			(1.323)			(1.155)
Postdoc			-4.283***			-2.250**
			(1.057)			(1.029)
Publication or R&R (1+)			3.409***			3.856***
			(1.121)			(1.000)
# applications (51-100)			5.974***			4.429***
			(1.503)			(1.418)
# applications (101-150)			7.295***			5.930***
			(1.440)			(1.336)
# applications (151-200)			9.924***			6.919***
			(1.820)			(1.653)
# applications (201+)			13.863***			10.203***
			(1.690)			(1.431)
AEA signals (2)			2.737**			1.330
			(1.156)			(1.066)
Constant	16.390***	33.762***	6.630	10.918***	23.706***	3.062
	(0.804)	(4.989)	(5.329)	(0.735)	(4.550)	(4.959)
Above-median = Below-median (p-values from t-tests)	0.754	0.678	0.406	0.830	0.851	0.471
Observations	417	403	394	417	403	394
Sample	Post-market	Post-market	Post-market	Post-market	Post-market	Post-market

OLS estimates with robust standard errors in parentheses. Significance levels: * $p < 0.10$, ** $p < 0.05$, *** $p < 0.01$.

Table S29. Number of Flyouts (ITT) — Median Split by Quote-tweet Specificity

**Dependent variable:**	**Number of Flyouts**					
	All Jobs			Tenure-Track Jobs		
	(1)	(2)	(3)	(4)	(5)	(6)
Tweet above median specificity	1.312	1.895**	1.898**	1.196	1.389*	1.427*
	(0.854)	(0.827)	(0.809)	(0.766)	(0.763)	(0.767)
Tweet below median specificity	0.445	0.844	0.486	0.499	0.727	0.399
	(0.570)	(0.576)	(0.528)	(0.490)	(0.497)	(0.464)
No tweet	0.907	1.635	1.501	-0.377	0.095	0.001
	(1.438)	(1.396)	(1.226)	(0.759)	(0.759)	(0.682)
URG	0.415	0.335	0.320	0.374	0.190	0.272
	(0.531)	(0.536)	(0.529)	(0.465)	(0.469)	(0.473)
Age		-0.259***	-0.118**		-0.167***	-0.092*
		(0.058)	(0.059)		(0.051)	(0.050)
Parental income class		0.760**	0.524		0.194	0.083
		(0.321)	(0.328)		(0.269)	(0.276)
US citizen		0.603	0.506		-0.831*	-0.987**
		(0.642)	(0.613)		(0.449)	(0.449)
Twitter account		0.571	0.589		0.387	0.489
		(0.554)	(0.535)		(0.443)	(0.433)
Top 30 PhD institution			2.303***			2.159***
			(0.488)			(0.421)
First-time JMC			1.832***			1.055**
			(0.497)			(0.451)
Predoc			0.553			0.057
			(0.621)			(0.497)
Postdoc			-1.003**			-0.333
			(0.471)			(0.430)
Publication or R&R (1+)			0.970**			1.106***
			(0.486)			(0.411)
# applications (51-100)			2.147***			1.480**
			(0.645)			(0.593)
# applications (101-150)			3.143***			2.196***
			(0.680)			(0.619)
# applications (151-200)			2.938***			1.761***
			(0.770)			(0.671)
# applications (201+)			2.753***			1.446***
			(0.668)			(0.542)
AEA signals (2)			0.547			0.515
			(0.515)			(0.463)
Constant	5.341***	11.044***	1.214	3.320***	7.920***	1.384
	(0.330)	(2.152)	(2.428)	(0.275)	(1.855)	(2.008)
Above-median = Below-median (p-values from t-tests)	0.326	0.223	0.084	0.363	0.383	0.161
Observations	417	403	394	417	403	394
Sample	Post-market	Post-market	Post-market	Post-market	Post-market	Post-market

OLS estimates with robust standard errors in parentheses. Significance levels: * $p < 0.10$, ** $p < 0.05$, *** $p < 0.01$.

Table S30. Number of Offers (ITT) — Median Split by Quote-tweet Specificity

**Dependent variable:**	**Number of Offers**					
	All Jobs			Tenure-Track Jobs		
	(1)	(2)	(3)	(4)	(5)	(6)
Tweet above median specificity	0.369<br>(0.342)	0.505<br>(0.331)	0.517<br>(0.337)	0.305<br>(0.316)	0.307<br>(0.305)	0.332<br>(0.318)
Tweet below median specificity	0.222<br>(0.276)	0.466<br>(0.285)	0.316<br>(0.271)	0.121<br>(0.234)	0.280<br>(0.245)	0.136<br>(0.241)
No tweet	0.416<br>(0.580)	0.633<br>(0.581)	0.568<br>(0.526)	-0.097<br>(0.356)	0.048<br>(0.373)	0.017<br>(0.357)
URG	0.155<br>(0.238)	0.116<br>(0.243)	0.060<br>(0.235)	0.301<br>(0.202)	0.223<br>(0.206)	0.247<br>(0.203)
Age		-0.156***<br>(0.025)	-0.099***<br>(0.028)		-0.088***<br>(0.021)	-0.065***<br>(0.022)
Parental income class		0.211<br>(0.157)	0.135<br>(0.146)		-0.072<br>(0.144)	-0.089<br>(0.134)
US citizen		-0.327<br>(0.264)	-0.433*<br>(0.258)		-0.743***<br>(0.192)	-0.853***<br>(0.197)
Twitter account		0.212<br>(0.264)	0.241<br>(0.263)		-0.039<br>(0.220)	0.015<br>(0.228)
Top 30 PhD institution			0.823***<br>(0.230)			0.817***<br>(0.203)
First-time JMC			0.760***<br>(0.278)			0.263<br>(0.247)
Predoc			0.245<br>(0.343)			-0.111<br>(0.260)
Postdoc			-0.498*<br>(0.268)			-0.125<br>(0.237)
Publication or R&R (1+)			0.603**<br>(0.246)			0.619***<br>(0.215)
# applications (51-100)			1.167***<br>(0.316)			0.676**<br>(0.290)
# applications (101-150)			1.228***<br>(0.337)			0.785**<br>(0.310)
# applications (151-200)			1.186***<br>(0.397)			0.644*<br>(0.356)
# applications (201+)			1.183***<br>(0.322)			0.605**<br>(0.282)
AEA signals (2)			0.475*<br>(0.254)			0.363<br>(0.226)
Constant	2.932***<br>(0.175)	7.203***<br>(1.007)	2.898**<br>(1.170)	1.468***<br>(0.142)	4.541***<br>(0.889)	2.091**<br>(0.950)
Above-median = Below-median<br>(p-values from t-tests)	0.699	0.914	0.578	0.602	0.937	0.570
Observations	417	403	394	417	403	394
Sample	Post-market	Post-market	Post-market	Post-market	Post-market	Post-market

OLS estimates with robust standard errors in parentheses. Significance levels: * $p < 0.10$, ** $p < 0.05$, *** $p < 0.01$.

Table S31. Number of Interviews (ITT) — Median Split by Quote-tweet Endorsement

**Dependent variable:**	**Number of Interviews**					
	All Jobs			Tenure-Track Jobs		
	(1)	(2)	(3)	(4)	(5)	(6)
Tweet above median endorsement	0.898	1.498	1.230	1.306	1.640	1.335
	(1.577)	(1.583)	(1.522)	(1.382)	(1.415)	(1.385)
Tweet below median endorsement	1.697	2.337	0.901	1.781	2.020	0.923
	(1.660)	(1.676)	(1.351)	(1.421)	(1.464)	(1.203)
No tweet	1.423	2.754	2.493	0.008	1.069	0.811
	(3.359)	(3.471)	(2.638)	(2.309)	(2.401)	(2.004)
URG	1.092	1.065	0.739	0.342	0.168	0.138
	(1.269)	(1.284)	(1.134)	(1.082)	(1.118)	(1.038)
Age		-0.672***	-0.265**		-0.429***	-0.166
		(0.141)	(0.130)		(0.129)	(0.124)
Parental income class		0.197	-0.517		-0.385	-0.839
		(0.755)	(0.699)		(0.639)	(0.599)
US citizen		2.042	1.541		-0.540	-0.973
		(1.486)	(1.304)		(1.219)	(1.140)
Twitter account		2.994**	2.902***		1.723	1.862*
		(1.234)	(1.110)		(1.074)	(0.979)
Top 30 PhD institution			5.835***			6.021***
			(1.134)			(1.025)
First-time JMC			2.460**			2.040*
			(1.179)			(1.170)
Predoc			0.358			-0.155
			(1.330)			(1.163)
Postdoc			-4.249***			-2.234**
			(1.056)			(1.029)
Publication or R&R (1+)			3.423***			3.874***
			(1.111)			(0.991)
# applications (51-100)			5.994***			4.440***
			(1.510)			(1.423)
# applications (101-150)			7.378***			5.975***
			(1.453)			(1.346)
# applications (151-200)			10.043***			7.004***
			(1.815)			(1.635)
# applications (201+)			13.980***			10.300***
			(1.705)			(1.441)
AEA signals (2)			2.698**			1.301
			(1.154)			(1.066)
Constant	16.387***	34.023***	6.828	10.916***	23.814***	3.200
	(0.804)	(5.006)	(5.336)	(0.734)	(4.566)	(4.974)
Above-median = Below-median (p-values from t-tests)	0.684	0.663	0.845	0.777	0.822	0.783
Observations	417	403	394	417	403	394
Sample	Post-market	Post-market	Post-market	Post-market	Post-market	Post-market

OLS estimates with robust standard errors in parentheses. Significance levels: * $p < 0.10$, ** $p < 0.05$, *** $p < 0.01$.

Table S32. Number of Flyouts (ITT) — Median Split by Quote-tweet Endorsement

**Dependent variable:**	**Number of Flyouts**					
	All Jobs			Tenure-Track Jobs		
	(1)	(2)	(3)	(4)	(5)	(6)
Tweet above median endorsement	1.077	1.531**	1.264*	0.944	1.155*	0.888
	(0.710)	(0.691)	(0.687)	(0.641)	(0.640)	(0.657)
Tweet below median endorsement	0.453	0.923	0.740	0.567	0.782	0.659
	(0.632)	(0.639)	(0.583)	(0.538)	(0.546)	(0.502)
No tweet	0.895	1.616	1.485	-0.385	0.083	-0.010
	(1.438)	(1.398)	(1.229)	(0.758)	(0.760)	(0.684)
URG	0.442	0.361	0.344	0.395	0.206	0.288
	(0.530)	(0.537)	(0.532)	(0.460)	(0.467)	(0.473)
Age		-0.262***	-0.122**		-0.168***	-0.095*
		(0.058)	(0.060)		(0.051)	(0.051)
Parental income class		0.732**	0.485		0.176	0.054
		(0.317)	(0.324)		(0.266)	(0.274)
US citizen		0.548	0.432		-0.866*	-1.044**
		(0.641)	(0.617)		(0.454)	(0.456)
Twitter account		0.583	0.619		0.395	0.516
		(0.554)	(0.533)		(0.443)	(0.432)
Top 30 PhD institution			2.246***			2.117***
			(0.498)			(0.429)
First-time JMC			1.826***			1.054**
			(0.500)			(0.451)
Predoc			0.540			0.052
			(0.621)			(0.497)
Postdoc			-0.983**			-0.309
			(0.475)			(0.431)
Publication or R&R (1+)			0.993**			1.116***
			(0.489)			(0.410)
# applications (51-100)			2.161***			1.494**
			(0.655)			(0.599)
# applications (101-150)			3.199***			2.254***
			(0.690)			(0.630)
# applications (151-200)			3.045***			1.843***
			(0.760)			(0.659)
# applications (201+)			2.876***			1.527***
			(0.686)			(0.547)
AEA signals (2)			0.511			0.487
			(0.515)			(0.462)
Constant	5.334***	11.182***	1.388	3.314***	8.007***	1.522
	(0.330)	(2.153)	(2.446)	(0.275)	(1.863)	(2.029)
Above-median = Below-median (p-values from t-tests)	0.435	0.438	0.485	0.584	0.584	0.729
Observations	417	403	394	417	403	394
Sample	Post-market	Post-market	Post-market	Post-market	Post-market	Post-market

OLS estimates with robust standard errors in parentheses. Significance levels: * $p < 0.10$, ** $p < 0.05$, *** $p < 0.01$.

Table S33. Number of Offers (ITT) — Median Split by Quote-tweet Endorsement

**Dependent variable:**	**Number of Offers**					
	All Jobs			Tenure-Track Jobs		
	(1)	(2)	(3)	(4)	(5)	(6)
Tweet above median endorsement	0.403	0.537*	0.464	0.300	0.353	0.273
	(0.304)	(0.298)	(0.301)	(0.282)	(0.271)	(0.285)
Tweet below median endorsement	0.153	0.423	0.315	0.080	0.226	0.144
	(0.299)	(0.312)	(0.298)	(0.248)	(0.269)	(0.268)
No tweet	0.414	0.632	0.565	-0.100	0.047	0.014
	(0.579)	(0.581)	(0.526)	(0.355)	(0.372)	(0.357)
URG	0.161	0.118	0.063	0.307	0.225	0.250
	(0.237)	(0.242)	(0.235)	(0.201)	(0.205)	(0.202)
Age		-0.155***	-0.099***		-0.088***	-0.066***
		(0.026)	(0.028)		(0.022)	(0.022)
Parental income class		0.211	0.130		-0.071	-0.094
		(0.158)	(0.147)		(0.147)	(0.137)
US citizen		-0.327	-0.443*		-0.741***	-0.862***
		(0.261)	(0.256)		(0.190)	(0.196)
Twitter account		0.209	0.242		-0.044	0.017
		(0.265)	(0.264)		(0.221)	(0.229)
Top 30 PhD institution			0.815***			0.809***
			(0.232)			(0.206)
First-time JMC			0.758***			0.261
			(0.278)			(0.247)
Predoc			0.240			-0.115
			(0.345)			(0.263)
Postdoc			-0.500*			-0.126
			(0.270)			(0.239)
Publication or R&R (1+)			0.610**			0.625***
			(0.244)			(0.211)
# applications (51-100)			1.167***			0.676**
			(0.318)			(0.292)
# applications (101-150)			1.228***			0.787**
			(0.341)			(0.315)
# applications (151-200)			1.199***			0.657*
			(0.396)			(0.353)
# applications (201+)			1.204***			0.625**
			(0.325)			(0.283)
AEA signals (2)			0.470*			0.358
			(0.254)			(0.226)
Constant	2.930***	7.198***	2.917**	1.466***	4.532***	2.111**
	(0.175)	(1.009)	(1.172)	(0.142)	(0.894)	(0.957)
Above-median = Below-median (p-values from t-tests)	0.494	0.751	0.679	0.510	0.706	0.709
Observations	417	403	394	417	403	394
Sample	Post-market	Post-market	Post-market	Post-market	Post-market	Post-market

OLS estimates with robust standard errors in parentheses. Significance levels: * $p < 0.10$, ** $p < 0.05$, *** $p < 0.01$.

Table S34. Number of Interviews (ITT) — Median Split by Influencers' Google Scholar Citations

**Dependent variable:**	**Number of Interviews**					
	All Jobs			Tenure-Track Jobs		
	(1)	(2)	(3)	(4)	(5)	(6)
Above-median citation influencer	1.429<br>(1.673)	2.694<br>(1.731)	2.831**<br>(1.423)	1.690<br>(1.426)	2.337<br>(1.492)	2.410*<br>(1.299)
Below-median citation influencer	1.226<br>(1.484)	1.465<br>(1.474)	-0.117<br>(1.284)	1.126<br>(1.259)	1.289<br>(1.275)	-0.007<br>(1.137)
URG	1.106<br>(1.268)	1.143<br>(1.277)	0.886<br>(1.116)	0.329<br>(1.080)	0.184<br>(1.110)	0.219<br>(1.020)
Age		-0.674***<br>(0.141)	-0.275**<br>(0.129)		-0.432***<br>(0.129)	-0.173<br>(0.123)
Parental income class		0.252<br>(0.758)	-0.416<br>(0.697)		-0.335<br>(0.641)	-0.746<br>(0.602)
US citizen		2.097<br>(1.480)	1.603<br>(1.313)		-0.511<br>(1.211)	-0.934<br>(1.149)
Twitter account		2.932**<br>(1.240)	2.828**<br>(1.116)		1.729<br>(1.068)	1.861*<br>(0.976)
Top 30 PhD institution			5.915***<br>(1.119)			6.101***<br>(1.016)
First-time JMC			2.704**<br>(1.143)			2.197*<br>(1.147)
Predoc			0.580<br>(1.322)			-0.029<br>(1.147)
Postdoc			-4.019***<br>(0.999)			-2.028**<br>(0.989)
Publication or R&R (1+)			3.352***<br>(1.125)			3.796***<br>(1.004)
# applications (51-100)			6.156***<br>(1.503)			4.685***<br>(1.434)
# applications (101-150)			7.377***<br>(1.435)			6.088***<br>(1.343)
# applications (151-200)			9.885***<br>(1.808)			7.015***<br>(1.639)
# applications (201+)			14.049***<br>(1.710)			10.452***<br>(1.467)
AEA signals (2)			2.942**<br>(1.156)			1.468<br>(1.070)
Constant	16.383***<br>(0.803)	33.972***<br>(4.977)	6.423<br>(5.319)	10.920***<br>(0.733)	23.767***<br>(4.546)	2.757<br>(4.938)
Above-median = Below-median<br>(p-values from t-tests)	0.915	0.511	0.051	0.722	0.509	0.070
Observations	417	403	394	417	403	394
Sample	Post-market	Post-market	Post-market	Post-market	Post-market	Post-market

Notes: This table presents the intent-to-treat (ITT) effect, by influencers' Google Scholar citations (median split), of being quote-tweeted by established economists on Twitter on the number of interviews received by job market candidates. For a description of the variables, see the table notes in Appendix Table S10. OLS estimates with robust standard errors in parentheses. Significance levels: * $p < 0.10$, ** $p < 0.05$, *** $p < 0.01$.

Table S35. Number of Flyouts (ITT) — Median Split by Influencers' Google Scholar Citations

**Dependent variable:**	**Number of Flyouts**					
	All Jobs			Tenure-Track Jobs		
	(1)	(2)	(3)	(4)	(5)	(6)
Above-median citation influencer	1.324*	2.140***	2.184***	1.277*	1.683**	1.698***
	(0.734)	(0.742)	(0.665)	(0.670)	(0.684)	(0.650)
Below-median citation influencer	0.335	0.600	0.109	0.105	0.251	-0.159
	(0.577)	(0.559)	(0.526)	(0.451)	(0.449)	(0.429)
URG	0.464	0.413	0.432	0.398	0.219	0.336
	(0.524)	(0.529)	(0.514)	(0.454)	(0.456)	(0.456)
Age		-0.270***	-0.130**		-0.175***	-0.100**
		(0.058)	(0.059)		(0.052)	(0.051)
Parental income class		0.791**	0.559*		0.236	0.130
		(0.315)	(0.321)		(0.265)	(0.272)
US citizen		0.570	0.467		-0.849*	-1.016**
		(0.653)	(0.629)		(0.458)	(0.463)
Twitter account		0.593	0.596		0.434	0.532
		(0.557)	(0.533)		(0.436)	(0.423)
Top 30 PhD institution			2.306***			2.185***
			(0.493)			(0.423)
First-time JMC			1.988***			1.155**
			(0.499)			(0.460)
Predoc			0.686			0.124
			(0.620)			(0.487)
Postdoc			-0.801*			-0.153
			(0.463)			(0.420)
Publication or R&R (1+)			0.925*			1.055**
			(0.486)			(0.412)
# applications (51-100)			2.320***			1.719***
			(0.662)			(0.613)
# applications (101-150)			3.264***			2.367***
			(0.689)			(0.634)
# applications (151-200)			2.990***			1.898***
			(0.745)			(0.671)
# applications (201+)			2.945***			1.687***
			(0.671)			(0.563)
AEA signals (2)			0.670			0.604
			(0.508)			(0.461)
Constant	5.327***	11.287***	1.082	3.313***	8.048***	1.131
	(0.329)	(2.132)	(2.420)	(0.274)	(1.861)	(2.004)
Above-median = Below-median (p-values from t-tests)	0.208	0.043	0.002	0.073	0.028	0.002
Observations	417	403	394	417	403	394
Sample	Post-market	Post-market	Post-market	Post-market	Post-market	Post-market

Notes: This table presents the intent-to-treat (ITT) effect, by influencers' Google Scholar citations (median split), of being quote-tweeted by established economists on Twitter on the number of flyouts received by job market candidates. For a description of the variables, see the table notes in Appendix Table S10. OLS estimates with robust standard errors in parentheses. Significance levels: * $p < 0.10$, ** $p < 0.05$, *** $p < 0.01$.

Table S36. Number of Offers (ITT) — Median Split by Influencers' Google Scholar Citations

**Dependent variable:**	**Number of Offers**					
	All Jobs			Tenure-Track Jobs		
	(1)	(2)	(3)	(4)	(5)	(6)
Above-median citation influencer	0.286<br>(0.304)	0.571*<br>(0.307)	0.595**<br>(0.279)	0.347<br>(0.273)	0.459*<br>(0.276)	0.460*<br>(0.268)
Below-median citation influencer	0.295<br>(0.280)	0.439<br>(0.284)	0.253<br>(0.285)	0.003<br>(0.227)	0.113<br>(0.233)	-0.043<br>(0.239)
URG	0.161<br>(0.236)	0.125<br>(0.241)	0.080<br>(0.234)	0.308<br>(0.198)	0.227<br>(0.202)	0.264<br>(0.198)
Age		-0.156***<br>(0.026)	-0.101***<br>(0.027)		-0.089***<br>(0.022)	-0.067***<br>(0.021)
Parental income class		0.215<br>(0.158)	0.141<br>(0.147)		-0.057<br>(0.147)	-0.074<br>(0.137)
US citizen		-0.326<br>(0.262)	-0.437*<br>(0.259)		-0.738***<br>(0.190)	-0.856***<br>(0.198)
Twitter account		0.208<br>(0.264)	0.237<br>(0.261)		-0.032<br>(0.218)	0.023<br>(0.226)
Top 30 PhD institution			0.823***<br>(0.231)			0.827***<br>(0.205)
First-time JMC			0.789***<br>(0.278)			0.291<br>(0.248)
Predoc			0.271<br>(0.340)			-0.093<br>(0.254)
Postdoc			-0.467*<br>(0.267)			-0.079<br>(0.232)
Publication or R&R (1+)			0.596**<br>(0.246)			0.605***<br>(0.215)
# applications (51-100)			1.188***<br>(0.322)			0.738**<br>(0.299)
# applications (101-150)			1.239***<br>(0.341)			0.823***<br>(0.310)
# applications (151-200)			1.183***<br>(0.399)			0.672*<br>(0.362)
# applications (201+)			1.206***<br>(0.331)			0.663**<br>(0.292)
AEA signals (2)			0.498**<br>(0.253)			0.390*<br>(0.223)
Constant	2.930***<br>(0.175)	7.217***<br>(1.012)	2.879**<br>(1.178)	1.466***<br>(0.142)	4.545***<br>(0.903)	2.011**<br>(0.959)
Above-median = Below-median<br>(p-values from t-tests)	0.981	0.696	0.281	0.265	0.259	0.096
Observations	417	403	394	417	403	394
Sample	Post-market	Post-market	Post-market	Post-market	Post-market	Post-market

Notes: This table presents the intent-to-treat (ITT) effect, by influencers' Google Scholar citations (median split), of being quote-tweeted by established economists on Twitter on the number of offers received by job market candidates. For a description of the variables, see the table notes in Appendix Table S10. OLS estimates with robust standard errors in parentheses. Significance levels: * $p < 0.10$, ** $p < 0.05$, *** $p < 0.01$.

Table S37. Number of Interviews (ITT) — Heterogeneous Effects by Top 30 Institution

**Dependent variable:**	**Number of Interviews**					
	All Jobs			Tenure-Track Jobs		
	(1)	(2)	(3)	(4)	(5)	(6)
Treatment group	-0.938	-0.201	-0.766	-0.271	0.062	-0.353
	(1.620)	(1.675)	(1.522)	(1.293)	(1.356)	(1.290)
Top 30 PhD institution	5.071***	5.104***	3.730***	5.145***	5.286***	4.506***
	(1.425)	(1.447)	(1.419)	(1.276)	(1.286)	(1.296)
Treatment group × Top 30 PhD institution	4.746**	4.237*	4.328**	3.445*	3.145	3.143*
	(2.291)	(2.295)	(2.089)	(1.958)	(1.978)	(1.837)
URG	1.770	1.671	0.804	0.931	0.686	0.149
	(1.248)	(1.266)	(1.143)	(1.056)	(1.094)	(1.039)
Age		-0.605***	-0.255**		-0.370***	-0.159
		(0.135)	(0.128)		(0.122)	(0.122)
Parental income class		-0.259	-0.511		-0.812	-0.825
		(0.734)	(0.698)		(0.612)	(0.598)
US citizen		1.801	1.535		-0.786	-0.989
		(1.456)	(1.302)		(1.192)	(1.132)
Twitter account		3.658***	2.974***		2.402**	1.970**
		(1.216)	(1.122)		(1.059)	(0.985)
First-time JMC			2.486**			2.020*
			(1.179)			(1.173)
Predoc			0.336			-0.220
			(1.289)			(1.127)
Postdoc			-4.355***			-2.292**
			(1.092)			(1.065)
Publication or R&R (1+)			3.304***			3.767***
			(1.113)			(0.995)
# applications (51-100)			5.833***			4.426***
			(1.498)			(1.428)
# applications (101-150)			7.463***			6.145***
			(1.440)			(1.352)
# applications (151-200)			9.752***			6.921***
			(1.826)			(1.639)
# applications (200+)			13.960***			10.370***
			(1.701)			(1.463)
AEA signal sent: 2			2.580**			1.185
			(1.152)			(1.070)
Constant	13.788***	30.022***	7.651	8.308***	19.885***	3.700
	(1.087)	(4.803)	(5.261)	(0.928)	(4.318)	(4.911)
Treatment × Top 30 + Treatment = 0 (p-values from t-tests)	0.027	0.019	0.023	0.043	0.043	0.059
Observations	417	403	394	417	403	394
Sample	Post-market	Post-market	Post-market	Post-market	Post-market	Post-market

Notes: This table presents the heterogeneous intent-to-treat (ITT) effects of being quote-tweeted by established economists on Twitter on the number of job interviews received by candidates, comparing those from top 30 PhD institutions versus other institutions. OLS estimates with robust standard errors in parentheses. Significance levels: * $p < 0.10$, ** $p < 0.05$, *** $p < 0.01$.

Table S38. Number of Flyouts (ITT) — Heterogeneous Effects by Top 30 Institution

**Dependent variable:**	**Number of Flyouts**					
	All Jobs			Tenure-Track Jobs		
	(1)	(2)	(3)	(4)	(5)	(6)
Treatment group	-0.207<br>(0.639)	0.240<br>(0.647)	0.100<br>(0.626)	-0.004<br>(0.520)	0.151<br>(0.544)	0.072<br>(0.543)
Top 30 PhD institution	2.097***<br>(0.596)	1.805***<br>(0.598)	1.238**<br>(0.621)	1.943***<br>(0.507)	1.759***<br>(0.506)	1.481***<br>(0.532)
Treatment group × Top 30 PhD institution	2.068**<br>(0.941)	2.026**<br>(0.939)	2.072**<br>(0.914)	1.320*<br>(0.781)	1.389*<br>(0.784)	1.333*<br>(0.768)
URG	0.721<br>(0.519)	0.585<br>(0.532)	0.368<br>(0.540)	0.602<br>(0.451)	0.366<br>(0.459)	0.275<br>(0.476)
Age		-0.238***<br>(0.056)	-0.119**<br>(0.059)		-0.148***<br>(0.050)	-0.091*<br>(0.051)
Parental income class		0.545*<br>(0.308)	0.488<br>(0.320)		0.020<br>(0.255)	0.065<br>(0.272)
US citizen		0.429<br>(0.638)	0.421<br>(0.621)		-0.972**<br>(0.445)	-1.056**<br>(0.452)
Twitter account		0.886<br>(0.539)	0.674<br>(0.538)		0.682<br>(0.424)	0.588<br>(0.428)
First-time JMC			1.840***<br>(0.499)			1.026**<br>(0.453)
Predoc			0.535<br>(0.612)			0.001<br>(0.485)
Postdoc			-1.011**<br>(0.481)			-0.326<br>(0.438)
Publication or R&R (1+)			0.917*<br>(0.489)			1.062**<br>(0.414)
# applications (51-100)			2.107***<br>(0.652)			1.537**<br>(0.602)
# applications (101-150)			3.292***<br>(0.691)			2.374***<br>(0.632)
# applications (151-200)			2.934***<br>(0.758)			1.868***<br>(0.663)
# applications (200+)			2.861***<br>(0.667)			1.600***<br>(0.551)
AEA signal sent: 2			0.448<br>(0.507)			0.424<br>(0.458)
Constant	4.259***<br>(0.406)	9.785***<br>(2.033)	1.793<br>(2.389)	2.333***<br>(0.316)	6.682***<br>(1.771)	1.685<br>(1.997)
Treatment × Top 30 + Treatment = 0<br>(p-values from t-tests)	0.014	0.003	0.004	0.049	0.020	0.034
Observations	417	403	394	417	403	394
Sample	Post-market	Post-market	Post-market	Post-market	Post-market	Post-market

Notes: This table presents the heterogeneous intent-to-treat (ITT) effects of being quote-tweeted by established economists on Twitter on the number of flyouts received by candidates, comparing those from top 30 PhD institutions versus other institutions. OLS estimates with robust standard errors in parentheses. Significance levels: * $p < 0.10$, ** $p < 0.05$, *** $p < 0.01$.

Table S39. Number of Offers (ITT) — Heterogeneous Effects by Top 30 Institution

**Dependent variable:**	**Number of Offers**					
	All Jobs			Tenure-Track Jobs		
	(1)	(2)	(3)	(4)	(5)	(6)
Treatment group	0.185<br>(0.281)	0.368<br>(0.281)	0.315<br>(0.282)	0.153<br>(0.233)	0.218<br>(0.236)	0.215<br>(0.245)
Top 30 PhD institution	1.024***<br>(0.311)	0.965***<br>(0.318)	0.714**<br>(0.316)	0.914***<br>(0.252)	0.889***<br>(0.254)	0.841***<br>(0.258)
Treatment group × Top 30 PhD institution	0.210<br>(0.464)	0.201<br>(0.466)	0.203<br>(0.469)	-0.009<br>(0.395)	0.034<br>(0.400)	-0.064<br>(0.402)
URG	0.262<br>(0.233)	0.203<br>(0.238)	0.069<br>(0.236)	0.380*<br>(0.198)	0.286<br>(0.201)	0.244<br>(0.201)
Age		-0.147***<br>(0.025)	-0.099***<br>(0.028)		-0.081***<br>(0.021)	-0.066***<br>(0.022)
Parental income class		0.143<br>(0.154)	0.128<br>(0.148)		-0.127<br>(0.141)	-0.094<br>(0.138)
US citizen		-0.367<br>(0.259)	-0.445*<br>(0.257)		-0.778***<br>(0.187)	-0.866***<br>(0.194)
Twitter account		0.307<br>(0.264)	0.247<br>(0.264)		0.050<br>(0.220)	0.027<br>(0.228)
First-time JMC			0.765***<br>(0.275)			0.259<br>(0.248)
Predoc			0.249<br>(0.340)			-0.117<br>(0.253)
Postdoc			-0.497*<br>(0.267)			-0.115<br>(0.237)
Publication or R&R (1+)			0.598**<br>(0.246)			0.619***<br>(0.214)
# applications (51-100)			1.155***<br>(0.316)			0.695**<br>(0.292)
# applications (101-150)			1.239***<br>(0.340)			0.811***<br>(0.308)
# applications (151-200)			1.179***<br>(0.396)			0.681*<br>(0.355)
# applications (200+)			1.189***<br>(0.327)			0.630**<br>(0.284)
AEA signal sent: 2			0.466*<br>(0.255)			0.356<br>(0.226)
Constant	2.416***<br>(0.193)	6.571***<br>(0.982)	2.975**<br>(1.159)	1.012***<br>(0.154)	3.971***<br>(0.871)	2.094**<br>(0.947)
Treatment × Top 30 + Treatment = 0<br>(p-values from t-tests)	0.285	0.136	0.175	0.648	0.438	0.645
Observations	417	403	394	417	403	394
Sample	Post-market	Post-market	Post-market	Post-market	Post-market	Post-market

Notes: This table presents the heterogeneous intent-to-treat (ITT) effects of being quote-tweeted by established economists on Twitter on the number of job offers received by candidates, comparing those from top 30 PhD institutions versus other institutions. For a description of the variables, see the table notes in Appendix Table S10. OLS estimates with robust standard errors in parentheses. Significance levels: * $p < 0.10$, ** $p < 0.05$, *** $p < 0.01$.

Table S40. Number of Tweets Posted by Job Market Candidates — before and after the Intervention Period

	All Tweets	Econ-relevant Tweets
Treatment group	2.405	1.3278
	(2.446)	(1.727)
Post-intervention period	-4.544***	-3.665***
	(0.864)	(0.727)
URG	2.423	0.914
	(2.245)	(1.602)
Treatment group × Post-intervention period	1.400	1.407
	(1.792)	(1.411)
Constant	11.551***	9.367***
	(1.434)	(1.133)
Observations	353	353

Notes: For job market candidates who reported their Twitter handles, this table presents the Difference-in-Differences (DiD) estimates of how being quote-tweeted by established economists affected job market candidates' own tweet volumes. The treatment group consists of candidates who were quote-tweeted by established economists, while the control group consists of those who were not. Pre-treatment tweet counts cover Sep 28, 2022 - Nov 27, 2022 (two months before the intervention), while Post-treatment tweet counts cover Nov 28, 2022 - Jan 28, 2023 (two months during and after the intervention). DiD estimates are presented with robust standard errors clustered by individual job market candidate in parentheses. Significance levels: * $p < 0.10$, ** $p < 0.05$, *** $p < 0.01$.

4. Top 30 Institutions in and outside the US

Below is a summary of how we obtain the institution rankings:

1. Our list of top 30 institutions in the U.S. is obtained from the Top 30 Econ Schools from the U.S. News directly.
2. Our list of top 30 institutions outside the U.S. is obtained from Top 25% Economics Departments on RePEc (October 2022) after excluding all the U.S. institutions from this list.
3. Three institutions from our list of top 30 institutions in the U.S. are not in Tilburg's Top 100 list: California Institute of Technology, University of Rochester, and Washington University in St. Louis.
4. Six institutions from our list of top 30 institutions outside the U.S. are not in Tilburg's Top 100 list: University of Groningen, Sciences Po, Queen's University, University of Western Ontario, Aix-Marseille University, and CEMFI.
5. Five institutions from our list of top 30 institutions in the U.S. are not in the Top 100 Econ department on RePEc: California Institute of Technology, Carnegie Mellon University, Pennsylvania State University, University of Rochester, and University of North Carolina–Chapel Hill.

Table S41. Top 30 Institutions in the U.S.

Institution	Ranking
Harvard University	top10
Massachusetts Institute of Technology	top10
Stanford University	top10
Princeton University	top10
University of California, Berkeley	top10
University of Chicago	top10
Yale University	top10
Northwestern University	top10
Columbia University	top10
University of Pennsylvania	top10
New York University	top11-20
University of California–Los Angeles	top11-20
University of Michigan	top11-20
California Institute of Technology	top11-20
Cornell University	top11-20
University of California, San Diego	top11-20
University of Wisconsin	top11-20
Duke University	top11-20
University of Minnesota	top11-20
Brown University	top11-20
Carnegie Mellon University	top21-30
Boston University	top21-30
Johns Hopkins University	top21-30
University of Maryland	top21-30
University of Texas–Austin	top21-30
University of California, Davis	top21-30
Boston College	top21-30
Pennsylvania State University–University Park	top21-30
University of Rochester	top21-30
University of North Carolina–Chapel Hill	top21-30
University of Virginia	top21-30
Vanderbilt University	top21-30
Washington University in St. Louis	top21-30

Table S42. Top 30 Institutions outside the U.S.

Institution	Ranking
Paris School of Economics	1
Toulouse School of Economics (TSE)	2
Oxford University	3
Barcelona School of Economics (BSE)	4
University College London (UCL)	5
London School of Economics and Political Science	6
University of British Columbia	7
Universiteit van Tilburg	8
University of Warwick	9
University of Toronto	10
University of Zurich	11
Monash University	12
University of Nottingham	13
University of Cambridge	14
Rijksuniversiteit Groningen	15
Alma Mater Studiorum - Università di Bologna	16
Sciences Po	17
University of Queensland	18
Aarhus Universitet	19
Queen’s University	20
Erasmus Universiteit Rotterdam	21
KU Leuven	22
Vrije Universiteit Amsterdam	23
University of York	24
Universiteit van Amsterdam	25
University of Western Ontario	26
Australian National University	27
Aix-Marseille University	28
CEMFI	29
Københavns Universitet	30

5. Cosine Similarity

Cosine similarity is a widely used measure of semantic similarity between two documents in the area of information retrieval (3). To compute the cosine similarity between two documents, a and b, we converted each document into a collection of words, cleaned them,[†] took their union, and generated two vectors, A and B. Each entry in a vector represents the frequency of a word's appearance in that document. We then calculated the cosine value of the angle, θ, between the two word vectors as follows:

$$\cos\theta = \frac{\mathbf{A}^T \cdot \mathbf{B}}{\|\mathbf{A}\|\|\mathbf{B}\|} = \frac{\sum_{i=1}^{n} A_i B_i}{\sqrt{\sum_{i=1}^{n} A_i^2}\sqrt{\sum_{i=1}^{n} B_i^2}}. \quad \textbf{[S1]}$$

Cosine similarity, $\cos\theta \in [0, 1]$, is therefore a linguistic distance metric. A one-unit increase in the cosine similarity measure corresponds to a transition from a match with no overlapping words ($\cos\theta = 0$) to a perfect match ($\cos\theta = 1$). See the Electronic Companion EC.6. of (4) for examples of how to compute cosine similarity.

In our sample, the mean cosine similarity is 0.50 (stdev 0.079), indicating a good match.[‡] It has been applied in economic research which involves natural language processing (4). The pseudo code is presented as an Algorithm on the next page.

Algorithm 1 Match JMCs to Influencers Based on Cosine Similarity with Embeddings

```
Input:
   JMP abstracts
   Abstracts of influencers' top 20 most cited papers
   email_suffix_jmp - list of email suffixes corresponding to JMP authors' institutions
   email_suffix_influencer - list of email suffixes corresponding to influencers' institutions
Output:
   Influencer_Assignments - DataFrame with JMPs assigned to influencers
Initialize the SentenceTransformer model with "all-MiniLM-L6-v2"
Encode JMP abstracts and influencer abstracts to obtain embeddings_jmp and embeddings_influencer
Compute cosine_scores between embeddings_jmp and embeddings_influencer
Initialize Influencer_Assignments DataFrame with columns ['JMP', 'Influencer', 'Institution Match']
Initialize a list counter to keep track of how many JMPs are assigned to each influencer
for each Influencer in Influencer_data do
    Initialize a counter for JMP assignments to this Influencer
    for each JMP in JMP_data do
        if counter < 5 then
            Extract cosine similarity score for this JMP-Influencer pair from cosine_scores
            Identify if this JMP has the highest similarity score for this Influencer
            if JMP is the best match so far for this Influencer then
                Check if email suffix of JMP matches with email suffix of Influencer
                if email suffixes match then
                    Append to Influencer_Assignments with 'Institution Match' as True
                else
                    Append to Influencer_Assignments with 'Institution Match' as False
                Increment counter for this Influencer
        else
            Continue to the next Influencer
    Update counter with the final count for this Influencer
```

[†] Cleaning involves dropping "stop" words that appear frequently across all documents , such as "and" and "the," as well as converting variants of the same word, such as "consider," "considered," and "considering," into its root "consider."

[‡] Note that because this algorithm limits the maximum number of matches for each influencer to five, there were 30 JMPs left unmatched. These unmatched JMPs were not considered when computing the cosine similarity. We then manually matched these 30 JMPs to influencers.

6. Potential Mechanisms

To better understand our findings, we conduct an exploratory analysis of potential mechanisms driving the positive effects of social media promotion on academic job market outcomes. We examine three potential channels through which our intervention might operate: (1) **attention mechanism** - social media promotion may help JMCs stand out in the highly competitive economics job market, where typically hundreds of candidates compete for the same positions; (2) **endorsement mechanism** - quote-tweets from established scholars may be perceived as endorsements of JMCs, lending credibility to candidates through public recognition from respected figures in their fields; (3) **confidence mechanism** - receiving attention from established scholars may boost JMCs' confidence during interviews and job talks, potentially improving their performance in these critical evaluation stages.

Attention mechanism. Our intervention significantly increases tweet visibility, with JMP tweets in the treatment group receiving about 5 times as many views (4,922 vs. 952) and 4 times as many likes (15.9 vs. 4.1) as the control group. This increased visibility is particularly pronounced for JMCs assigned to high-reach influencers (those with above-median follower counts relative to all influencers in our sample, median: 13,450 followers). The ITT effect on views is almost three times as large for JMCs assigned to high-reach influencers as for those assigned to low-reach influencers ($p < 0.001$, column 3 in Appendix Table S24). The effect on likes is also larger for JMCs assigned to high-reach influencers, although this difference is not statistically significant (12.7 vs. 9.1, $p = 0.166$, column 6 in Appendix Table S24).

Interestingly, despite generating less visibility, we find that JMCs assigned to *low-reach* influencers achieve significantly better job market outcomes, securing 3.6 more interviews ($p = 0.012$, column 6 in Appendix Table S25), 1.4 more flyouts ($p = 0.024$, column 6 in Appendix Table S26), and 0.6 marginally more job offers for tenure-track positions ($p = 0.065$, column 6 in Appendix Table S27) compared to those assigned to high-reach influencers. This finding points to a nuanced relationship between the attention of the academic audience and the attention of a broader, less specialized follower base: we find that low-reach influencers also tweet less frequently during the intervention period (2.3 vs. 6.4 tweets per day, $p = 0.002$).[§] Thus, it is possible that each tweet captures greater attention from the academic audience, which could explain their stronger impact on job market outcomes.

Endorsement mechanism. Endorsement can operate through two channels: explicit endorsement through tweet language and implicit endorsement through academic reputation. Although our quote-tweet templates are designed to be neutral, our influencers are able to modify the text. To examine heterogeneity in treatment effects by tweet language, we ask three raters (one human research assistant and two large language models: ChatGPT-4 and Claude-3) to evaluate the quote-tweets on a 1-5 scale for specificity and endorsement.[¶] Analysis of the ratings reveals relatively low scores for both specificity (mean = 2.0, std = 0.88, median = 2) endorsement (mean = 1.7, std = 0.77, median = 1.3), suggesting most quote-tweets are neutral in tone.[‖]

We find that tweet language has limited impact on job market outcomes. While JMCs promoted with above-median specificity quote-tweets secure 1.4 marginally more flyouts than those with below-median specificity quote-tweets ($p = 0.084$, column 3 in Appendix Table S29), the level of specificity does not significantly affect the number of interviews or job offers (see Appendix Tables S28 and S30). Similarly, the level of endorsement in the quote-tweets does not significantly influence job market outcomes (see Appendix Table S31, S32, and S33). By contrast, academic reputation strongly affects the effectiveness of quote-tweets in achieving better job market outcomes. Specifically, JMCs assigned to influencers with above-median Google Scholar citations (median: 8,590) in our sample secure 2.9 marginally more interviews ($p = 0.051$ column 3 in Appendix Table S34), 2.1 more flyouts ($p = 0.002$, column 3 in Appendix Table S35), and 0.5 marginally more tenure-track offers ($p = 0.096$, column 6 in Appendix Table S36) compared to those assigned to below-median cited influencers.[**] Similarly, JMCs from the top 30 PhD institutions (in or outside the US) secure 4.3 more interviews ($p = 0.023$, column 3 in Appendix Table S37) and 2.1 more flyouts ($p = 0.004$, column 3 in Appendix Table S38) compared to other treated candidates, though the difference in the effect size on offers (0.2 more) is not statistically significant ($p = 0.175$, column 3 in Appendix Table S39). These findings suggest that in academic social media, who shares the work may matter more than how enthusiastically they share it.

Confidence mechanism. We examine whether being quote-tweeted by established scholars boosts candidate confidence during interviews and flyout visits. Since applications were submitted before our intervention, any confidence effects would be manifested in candidates' interview and job talk performance rather than in their ability to secure more interviews. Thus, if increased confidence is the mechanism behind our findings, we should observe larger effects for flyouts and offers compared to the number of interviews.

Our analysis shows greater effects (in relative terms) on the number of flyouts and offers for our sample compared to the number of interviews. However, this pattern may also reflect that our intervention occurred after many employers had already finalized their interview invitation decisions, limiting its potential impact on the initial screening stage. To further examine whether increased candidate confidence can explain our results, we use a difference-in-differences approach to compare changes in tweet volume between the treatment and control groups before and after our intervention. If JMCs experience greater confidence due to the intervention, they might feel more empowered to tweet about their work or other topics related to their academic fields. However, we find no significant differences in either overall tweeting or economics-specific content (see Appendix Table S40), suggesting that increased candidate confidence is unlikely to be a primary mechanism driving our results.

[§] Due to the new data restriction on X (see https://developer.twitter.com/docs/twitter-api/tweets/timelines), which only allows retrieval of the 3,200 most recent tweets per user, we were unable to access eight influencers' posting data from our intervention period when collecting data in April 2024. These eight influencers had posted more than 3,200 tweets between our intervention period and the data collection date, which pushed their intervention-period tweets beyond the API's historical reach. For these eight influencers, we top-code their daily posting activity at 19.2 tweets per day, which represents the top 1% of daily posting frequency among our influencers.

[¶] The level of agreement between human and AI raters is moderate to substantial, with Cohen's kappa values of 0.631 for specificity and 0.592 for endorsement. We use the average score across raters to keep the below- versus above-average groups roughly equal in size. See Appendix 10 for details on the rating instructions and procedure.

[‖] In our sample, three of the JMP tweets were retweeted instead of quote-tweeted. We put these three papers into the below-median group for specificity and endorsement, assigning the lowest ratings of 1, rather than placing them into the "No tweet" group.

[**] The relatively low Pearson correlation (0.286) between influencers' follower count and citation count indicates that academic reputation captures a dimension of influence different from that related to social media visibility (see Appendix Figure S1).

Overall, our analysis of potential mechanisms suggests that increased attention from relevant audiences and association with academic reputation both play a role in our findings. We find that quote-tweets appear most effective when coming from highly-cited scholars or when promoting candidates from top institutions. Our analysis further reveals that influencers with fewer followers have a stronger impact on candidates' job market outcomes. Since these influencers also tweet less frequently, it suggests that their more selective posting may attract more focused attention from academic audiences compared to the broader but less concentrated visibility achieved by frequent posters.

7. Correlates of Job Market Outcomes

This section provides an exploratory analysis of how candidates' personal and academic characteristics correlate with job market outcomes. Columns 3-5 of Table 1 in the main text presents these correlations for all types of jobs, while Appendix Tables S10-S12 additionally include the regression results for tenure track positions.

Two notable patterns emerge regarding candidates' personal characteristics. First, age is negatively associated with job market success, with older candidates receiving fewer interviews, flyouts, and offers. Specifically, each additional year of age corresponds to 0.3 fewer interviews ($p = 0.040$), 0.1 fewer flyouts ($p = 0.037$), and 0.1 fewer offers ($p < 0.001$). Second, US citizens receive 1.1 fewer flyouts ($p = 0.021$) and 0.9 fewer offers ($p < 0.001$) for tenure-track jobs compared to non-US-citizens. Other personal characteristics, such as parental income class, show no significant relationship with job market outcomes. Having a Twitter account is associated with receiving 2.8 more interviews ($p = 0.011$), though this effect does not extend to flyouts and offers.

Regarding candidates' professional characteristics, we find that candidates from top 30 universities (in or outside the US) and those with at least one published or revise-and-resubmit paper have better job market outcomes. Specifically, candidates from top 30 institutions receive 5.8 more interviews ($p < 0.001$), 2.2 more flyouts ($p < 0.001$), and 0.8 more offers ($p = 0.001$) compared to their peers from lower-ranked institutions. Candidates with at least one publication receive 3.4 more interviews ($p = 0.003$), one additional flyout ($p = 0.048$), and 0.6 more offers ($p = 0.015$). Candidates on the job market for the first time also fare better than those who had previously been on the market, receiving 2.5 more interviews ($p = 0.033$), 1.9 more flyouts ($p < 0.001$), and 0.8 more offers ($p = 0.006$). In contrast, postdoctoral experience is negatively associated with job market success, even after controlling for age and being on the job market for the first time. Postdocs receive 4.2 fewer interviews ($p < 0.001$), one fewer flyout ($p = 0.044$), and 0.5 fewer offers ($p = 0.068$).

Our results further show that submitting more job applications is positively associated with success across all stages of the job market, but with diminishing returns at higher application volumes. Compared to candidates applying to 1-50 jobs, those submitting 51-100 applications receive 5.9 more interviews ($p < 0.001$), while those applying to 101-150, 151- 200, and over 200 jobs secure 7.3 ($p < 0.001$), 9.9 ($p < 0.001$), and 13.9 ($p < 0.001$) more interviews, respectively. Candidates submitting more than 50 applications also receive between 2.1 and 3.2 more flyouts ($p \leq 0.002$) and approximately 1.2 more offers ($p \leq 0.003$) compared to those applying to fewer jobs. Finally, the use of the AEA signaling mechanism is associated with 2.7 more interviews ($p = 0.018$) and 0.5 marginally more offers ($p = 0.062$), highlighting the potential benefits of strategic signaling in the academic job market.

8. Post-market Survey

This appendix includes the questions and instructions for the post-market survey. Among the 519 participants randomized to treatment and control conditions, 417 reported their job market outcomes.[††] *Unless otherwise specified, the summary statistics presented in this Appendix are based on the 412 complete survey responses.*

To encourage participants to complete the post-market survey, we offer each JMC an unconditional $10 Amazon gift card.[‡‡] *We send non-respondents an email reminder up to six times. To ensure response accuracy, we follow up on any participants reporting outliers, including those reporting a pre-tax salary below $25K or above $600K as well as those who report more than 50 interviews. Most participants either confirm or revise their responses upon contact.*[§§] *To validate the accuracy of the self-reported salary information, we manually collected salary data for those who accepted job offers at public universities in the US. Among the 50 participants who provided salary information and accepted positions at US public universities, we were able to find official salary information for 31 of them. Among these 31 participants, the Pearson correlation between publicly available salary information in 2023 or 2024 and self-reported salary in 2023 is 0.77.*

Consent to Participate in Survey Research

Principal Investigators: Yan Chen (University of Michigan), Alain Cohn (University of Michigan), Jingyi Qiu (University of Michigan), Alvin Roth (Stanford University)

Online Consent

8.0.0.1. Description. We are conducting a follow-up survey to gain insight into the impact of social media on job market outcomes in economics. In this survey, you will be asked questions about your job applications, job market outcomes, and the use of social media.

[††] We removed three participants from our analysis who had completed the pre-market survey twice using different email addresses and were assigned to both treatment and control conditions. Of the 417 participants, 412 completed the post-market survey, and five emailed us their job market outcomes, one of whom had partially completed the survey before the part E.3. This participant's response was included in the summary statistics.

[‡‡] All monetary figures are in US dollars.

[§§] Four out of five participants with salaries above $600,000 revised their salary, while one confirmed it. Among six participants with salaries below $25,000, one confirmed, three revised, one did not respond (coded as "non-disclosed"), and one was not contacted because their job position and the exchange rate suggested a low salary. Seven out of 11 participants with more than 50 interviews confirmed their number, while four made revisions. We conjecture that the virtual interview format may have contributed to the high number of interviews for some participants.

8.0.0.2. Duration. The survey should take 5-10 minutes. Your participation is voluntary, and you may exit the survey at any time.

8.0.0.3. What will happen if you take part in this study. If you agree to participate, your responses might be linked to American Economic Association institutional records to draw information on job market outcomes. Once these data have been extracted, any information that can directly identify you will be removed from the data file and stored separately as part of the project.

8.0.0.4. What will be done with the information collected from the survey. The data are being collected by the research team (Yan Chen, Alain Cohn, Jingyi Qiu and Alvin Roth). Your responses will be held confidential. This means that only the research team will be able to access any identifying information about respondents. Additionally, your data will be anonymized and results will only be analyzed and reported at the aggregate level. Data may be stored in a repository for future research and you may be asked to participate in future surveys.

8.0.0.5. Benefits and risks of participation. In appreciation of your consideration for participating in our survey, we are offering a $10 Amazon gift card to you regardless of your survey completion. Moreover, the results of this study has the potential to help future cohorts of job market candidates. This study involves no more than minimal risk. You may discontinue participation at any time during the research activity.

8.0.0.6. Contact information. If you have questions about this study, you may contact Jingyi Qiu (jaqiu@umich.edu).

The University of Michigan Institutional Review Board Health Sciences and Behavioral Sciences has determined that this study is no more than minimal risk and exempt from on-going IRB oversight (IRB Study Number: HUM00221663).

Study Consent:

- No, I do not wish to participate in the survey. Please exit this survey now.
- Yes, I agree to participate in the survey. Please proceed to the survey.

8.1. Personal background.

1. What was your title or position in December 2022?
   (a) Ph.D. Student / Ph.D. Candidate (79.90%)
   (b) Postdoc (12.59%)
   (c) Lecturer (1.21%)
   (d) Assistant Professor (2.91%)
   (e) Other, please specify (3.39%)

   (Count 413)

8.2. Number of jobs JMC applied for.

1. How many jobs did you apply for?
   (a) 1-50 jobs (12.35%)
   (b) 51-100 jobs (17.19%)
   (c) 101-150 jobs (26.88%)
   (d) 151-200 jobs (19.61%)
   (e) 201+ jobs (23.97%)

   (Count 413)
2. Did you apply primarily for academic or non-academic jobs? [1 = primarily academic, 5 = primarily non-academic]
   (a) 1
   (b) 2
   (c) 3
   (d) 4
   (e) 5

   (Count 413, Mean 1.60, Std Dev 0.88, Median 1, Min 1, Max 5)
3. Did you send AEA job market signals?
   (a) Yes, I sent one signal (3.87%)
   (b) Yes, I sent two signals (76.27%)

   (c) No (19.85%)

   (Count 413)

4. If yes: How many interviews or fly-outs or job offers did you receive from the institutions/employers to which you send signals?

   (a) Interviews: 0/1/2
   (Count 331, Mean 0.57, Std Dev 0.69, Median 0, Min 0, Max 2)
   (b) Fly-outs: 0/1/2 (Count 331, Mean 0.24, Std Dev 0.49, Median 0, Min 0, Max 2)
   (c) Job offers: 0/1/2 (Count 331, Mean 0.14, Std Dev 0.38, Median 0, Min 0, Max 2)

8.3. Interviews/fly-outs/offers received by types.

1. How many interviews did you receive for the following types of jobs?

   (a) Tenure-track positions (or equivalent) at a college or university
   (Count 417, Mean 11.74, Std Dev 10.38, Median 9, Min 0, Max 60)
   (b) Non-tenure track positions at college or university, such as postdoc positions
   (Count 412, Mean 2.40, Std Dev 3.06, Median 2, Min 0, Max 22)
   (c) Public-sector: Government, non-profit and quasi-governmental organizations, such as central banks, IMF, World Bank
   (Count 412, Mean 1.77, Std Dev 2.98, Median 1, Min 0, Max 16)
   (d) Contract research organizations / think tanks, such as RAND, Mathematica, Research Triangle Institute)
   (Count 412, Mean 0.42, Std Dev 1.51, Median 0, Min 0, Max 25)
   (e) Private-sector: banking, finance, business, industry, consulting
   (Count 412, Mean 1.21, Std Dev 2.14, Median 0, Min 0, Max 12)

2. How many fly-outs (on-site visits) did you receive for the following types of jobs?

   (a) Tenure-track positions (or equivalent) at a college or university
   (Count 412, Mean 3.80, Std Dev 4.12, Median 2, Min 0, Max 24)
   (b) Non-tenure track positions at college or university, such as postdoc positions
   (Count 412, Mean 0.77, Std Dev 1.35, Median 0, Min 0, Max 12)
   (c) Public-sector: Government, non-profit and quasi-governmental organizations, such as central banks, IMF, World Bank
   (Count 412, Mean 0.82, Std Dev 1.64, Median 0, Min 0, Max 12)
   (d) Contract research organizations / think tanks, such as RAND, Mathematica, Research Triangle Institute)
   (Count 412, Mean 0.13, Std Dev 0.42, Median 0, Min 0, Max 3)
   (e) Private-sector: banking, finance, business, industry, consulting
   (Count 412, Mean 0.41, Std Dev 1.01, Median 0, Min 0, Max 6)

3. How many job offers did you receive for the following types of jobs?

   (a) Tenure-track positions (or equivalent) at a college or university
   (Count 417, Mean 1.68, Std Dev 2.02, Median 1, Min 0, Max 14)
   (b) Non-tenure track positions at college or university, such as postdoc positions
   (Count 412, Mean 0.74, Std Dev 1.04, Median 0, Min 0, Max 5)
   (c) Public-sector: Government, non-profit and quasi-governmental organizations, such as central banks, IMF, World Bank
   (Count 412, Mean 0.42, Std Dev 0.91, Median 0, Min 0, Max 7)
   (d) Contract research organizations / think tanks, such as RAND, Mathematica, Research Triangle Institute)
   (Count 412, Mean 0.09, Std Dev 0.36, Median 0, Min 0, Max 4)
   (e) Private-sector: banking, finance, business, industry, consulting
   (Count 412, Mean 0.22, Std Dev 0.58, Median 0, Min 0, Max 4)

4. (Optional) If you kept a record of your job applications, including the interview invitations, fly-out invitations, and job offers you received, and you are willing to share with us, could you upload your record here? We will never share the file outside of the research team.

5. Did you receive any very early or short-duration offers that made it hard to wait for other interviews/fly-outs/offers?

   (a) Yes (47.8%)
   (b) No (52.2%)

6. Did you register for and participate in the AEA scramble?

   (a) Yes (16.0%)
   (b) No (84.0%)

8.4. Job offer acceptance.

1. Did you accept a job offer?
   (a) Yes (91.7%)
   (b) No (8.3%)
2. What type of job offer did you accept?
   (a) Academia (college, university) (76.98%)
   (b) Government, including central banks (9.26%)
   (c) Non-profit or quasi-governmental organizations (e.g., IMF, World Bank) (3.97%)
   (d) Contract research organizations / think tanks (e.g., RAND, Mathematica, Research Triangle Institute) (3.70%)
   (e) Private-sector: tech, banking, finance, business, industry, consulting (5.56%)
   (f) Other, please specify___ (0.53%)

   (Count 378)
3. What is the name of the institution or organization at which you will be working?
4. Which of the following best describes your position?
   (a) Tenure Track Assistant Professor (50.40%)
   (b) Lecturer (4.00%)
   (c) Adjunct (0%)
   (d) Visiting Professor (2.67%)
   (e) Postdoctoral Fellow (18.13%)
   (f) Research Scientist (12.80%)
   (g) Other, please specify___ (12.00%)

   (Count 375)
5. In what country is your new job?
   (a) –Select Country–
6. How satisfied are you with your job placement?
   Rating: Not at all satisfied (1) - Very satisfied (7)
   7: (35.20%), 6: (31.20%), 5: (18.93%), 4: (6.40%), 3: (3.47%), 2: (3.20%), 1: (1.60%)
7. (Optional) At your new job, what will be your annual pre-tax salary for the coming year? If your base salary is not in USD, please report the approximate value in USD. (Count 281, Mean 107,969.42, Std Dev 62,582.35, Median 90,000, Min 140,000, Max 600,000)

8.5. Twitter.

1. Did you promote your JMP on Twitter? (check all that apply)
   (a) Yes, I wrote a separate tweet about my JMP BEFORE the Econ Job Market Helper tweeted my JMP (19.00%)
   (b) Yes, I wrote a separate tweet about my JMP AFTER the Econ Job Market Helper tweeted my JMP (10.18%)
   (c) Yes, I retweeted/quote-tweeted my JMP tweet posted by the Econ Job Market Helper (26.47%)
   (d) No, I did not promote my JMP on Twitter (exclusive choice) (44.34%)

   (Count 408)
2. Did anyone else besides Econ Job Market Helper tweet about your JMP? (for example, your department placement officer, your advisor)
   (a) Yes, my placement officer (11.21%)
   (b) Yes, my advisor(s) (12.84%)
   (c) Yes, my co-author(s) (7.05%)
   (d) Yes, one or more renowned researcher (20.80%)
   (e) Yes, one or more researchers that I do not know (17.54%)
   (f) No, I am not on Twitter / I am not sure (30.56%)

   (Count 408)

8.6. Academic background.

1. As of November 2022, what was your research record?

   (a) Number of working papers: ______ (Count 408, Mean 3.04, Std Dev 1.60, Median 3, Min 0, Max 20)

   (b) Number of articles published or forthcoming in peer-reviewed journals or proceedings: ______ (Count 408, Mean 1.21, Std Dev 2.52, Median 0, Min 0, Max 20)

   (c) Number of R&R (Revised and Resubmitted, or under revision for resubmission) articles in peer-reviewed journals or proceedings: ______ (Count 408, Mean 0.368, Std Dev 0.66, Median 0, Min 0, Max 4)

2. Please upload the latest version of your CV as .pdf:

3. Please upload the latest version of your job market paper as .pdf:

4. What is the URL of your personal webpage?

   - My personal webpage URL is ____________
   - I don't have a personal webpage.

9. Prediction Study

This appendix includes the questions for the prediction study survey.[¶¶] *Among 80 economist influencers who received our prediction study survey, 31 influencers made predictions. On the Social Science Prediction Platform, 75 SSPP experts completed the prediction survey, of which 73 made predictions. Two SSPP experts reported that they had seen the results of this study at a conference and were therefore excluded from making forecasts. We therefore report the summary statistics of these influencers and SSPP experts respectively.*

The survey outlined our experimental design and provided the average and standard deviation of the job market outcomes of the control group, along with the sample size. SSPP experts and influencers were then asked to predict the expected increase in these outcomes for the treated participants using a 0 to 100% slider scale.[***] *The three most accurate predictors within each group received an Amazon gift card of $100.*

Principal Investigators: Yan Chen (Michigan), Alain Cohn (Michigan), Jingyi Qiu (Michigan), Alvin Roth (Stanford)

Consent Form

9.0.0.1. Task and duration. We will ask you to make predictions about the effects of social media promotion on job market outcomes. It should take you 5 minutes or less to complete the survey.

9.0.0.2. Compensation. The three most accurate colleagues will each receive a $100 Amazon gift card.

9.0.0.3. What will be done with the information collected from the survey. Your responses will be held confidential. Your data will be anonymized and results will only be analyzed and reported at the aggregate level.

9.0.0.4. Contact information. If you have questions about this study, you may contact Jingyi Qiu (jaqiu@umich.edu).

The University of Michigan Institutional Review Board Health Sciences and Behavioral Sciences has determined that this study is no more than minimal risk and exempt from on-going IRB oversight (IRB Study Number: HUM00221663).

Please indicate, in the box below, that you have read and understood this consent form, and that you agree to participate in this study.

- Yes, I agree to participate in the survey. Please proceed to the survey.
- No, I do not wish to participate in the survey. Please exit this survey now.

9.1. Experimental Design.

In November and December 2022, we conducted a Twitter experiment with econ job market candidates, which aims to assess the impact of social media promotion on job market outcomes.

9.1.0.1. Study details. We invited students who were on the economics job market to fill out a survey about their academic background and social media use. At the end of the survey, we asked them to summarize their job market papers (JMPs) so that we can tweet about it on our Twitter account, Econ Job Market Helper.

9.1.0.2. Treatment. We asked influential economists to "quote tweet" a random subset of the JMPs from candidates in their field on Twitter. We provided templates for the quote tweets (e.g., Retweeting a JMP from @*econ_jmp* to help publicize job market candidates in my field).

Now, we want to invite you to predict the results of this study.

The three most accurate colleagues will each receive a $100 Amazon gift card.

9.1.0.3. Questions.

1. Have you seen the results of this study at a conference?
   (a) Yes, I have seen the results of this study at a conference (Influencers: 0%, SSPP: 2.67%)
   (b) No, I haven't seen the results of this study (Influencers: 100%, SSPP: 97.33%)
2. Total number of job market candidates in our sample: 420

   Candidates in the control group on average received: 16.8 job interviews (standard deviation: 10.83)

   By what percentage did treated job market candidates receive more job interviews on average?

   If you predict there are no treatment effects on interviews, please move the slider to 0.

[¶¶] The sample size provided to influencers and SSPP experts in the prediction study was 420, as we were unaware at that point that some participants had completed the pre-market survey twice using different email addresses and were assigned to both treatment and control conditions, potentially reporting job market outcomes twice. The actual sample size used in the analysis is 417, after removing the duplicate participants.

[***] The survey design excluded options for predicting negative treatment effects, potentially skewing predicted outcomes upward.

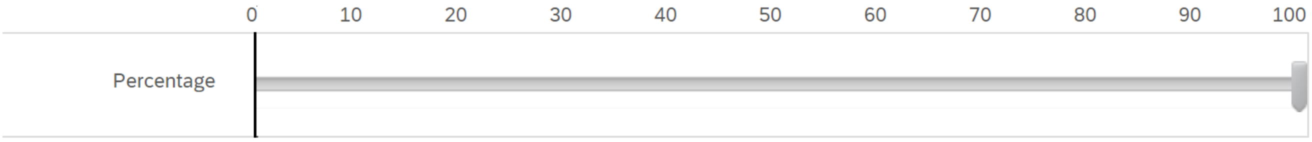
0
10
20
30
40
50
60
70
80
90
100
Percentage

(Influencers: Mean 11.71, Std Dev 9.22, Median 9, Min 0, Max 34)
(SSPP: Mean 17.59, Std Dev 18.73, Median 11, Min 0, Max 83)

3. Total number of job market candidates in our sample: 420

   Candidates in the control group on average received: 5.5 flyouts (standard deviation: 4.44)

   By what percentage did treated job market candidates receive more flyouts on average?

   If you predict there are no treatment effects on flyouts, please move the slider to 0.

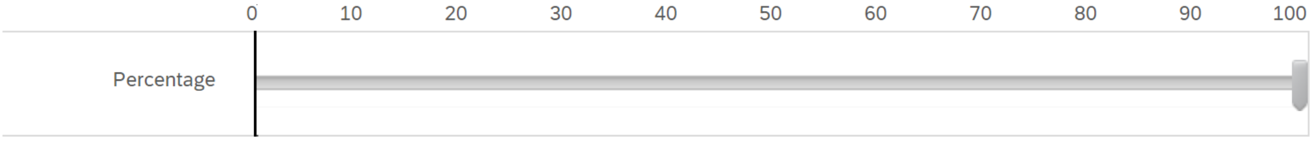

(Influencers: Mean 7.55, Std Dev 9.04, Median 4, Min 0, Max 35)
(SSPP: Mean 12.63, Std Dev 19.74, Median 6, Min 0, Max 100)

4. Total number of job market candidates in our sample: 420

   Candidates in the control group on average received: 3.0 job offers (standard deviation: 2.28)

   By what percentage did treated job market candidates receive more job offers on average?

   If you predict there are no treatment effects on job offers, please move the slider to 0.

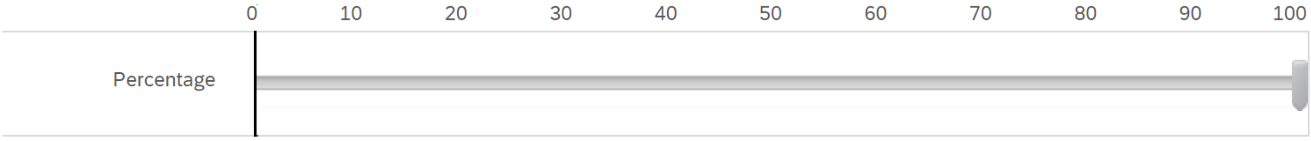

(Influencers: Mean 4.16, Std Dev 5.94, Median 2, Min 0, Max 21)
(SSPP: Mean 10.71, Std Dev 18.26, Median 4, Min 0, Max 100)

5. How confident are you with your predictions?
   (a) Not confident at all (Influencers: 35.48%, SSPP: 12.33%)
   (b) Slightly confident (Influencers: 38.71%, SSPP: 39.73%)
   (c) Moderately confident (Influencers: 25.81%, SSPP: 31.51%)
   (d) Confident (Influencers: 0%, SSPP: 12.33%)
   (e) Very confident (Influencers: 0%, SSPP: 4.11%)

6. (For economist influencers only) Are you still active on Twitter?
   (a) Yes, I am active on Twitter (Influencers: 83.87%)
   (b) No, I was active on Twitter but not anymore (Influencers: 16.13%)

7. (For SSPP experts only) Are you still active on Twitter?
   (a) Yes, I am active on Twitter (SSPP: 53.42%)
   (b) Yes, I was active on Twitter but not anymore (SSPP: 21.92%)
   (c) No, I am not on Twitter (SSPP: 24.66%)

8. (For economist influencers only) Would you like to be acknowledged in this study?
   (a) Yes, I would like to be acknowledged in this study (Influencers: 54.84%)
   (b) No, I do not want to be acknowledged in this study (Influencers: 45.16%)

9. (For economist influencers only) If you would like to be acknowledged in this study, what is the name you want us to display?

10. Instructions for Rating Quote-tweets

The instruction given to the human and GenAI raters used quote-tweets from our influencers as examples. To preserve the anonymity of our influencers and participants, we replace those examples with our made-up example in Appendix Figure S2. The raters received the same instructions.

Endorsement: From 1 (no endorsement) up to 5 (very strong endorsement).

1 - No endorsement: The quote tweet does not contain any words or phrases indicating support, appreciation, or recommendation of the job market candidate or their job market paper (JMP).

Example: To promote young scholars in my field, I am retweeting a JMP on curiosity-driven news consumption.

5 - Very strong endorsement: The quote tweet contains strong expressions of endorsement such as "the best" or "transformative" JMP.

Example: A very cool job market study of over 100,000 WeChat news articles reveals how curiosity drives news consumption. Their findings show that people are more likely to read articles when the headline sparks a salient question, the content seems more important, and the topics are more surprising.

Specificity: From 1 (not specific at all) up to 5 (very specific).

1 - Not Specific At All: The tweet provides no additional information about the job market paper's approach, findings, or the unique aspects of their research. It is generic and does not offer any details beyond possibly mentioning the candidate's name or field.

Example: Take a look at this young scholar in my field. Here is their JMP on curiosity-driven news consumption.

5 - Very specific: The tweet is very detailed, providing in-depth information about the job market paper's research approach, detailed findings, and the unique aspects of their JMP. It might include specific results, the significance of the research, and how it contributes to the field.

Example: A very cool job market study illustrates how curiosity drives news consumption. Their findings show people are more likely to read articles when the headline sparks a salient question, the content seems more important, and the topics are more surprising.

References

1. S Lundberg, J Stearns, Women in economics: Stalled progress. *J. Econ. Perspectives* **33**, 3–22 (2019).
2. A Bayer, GA Hoover, E Washington, How you can work to increase the presence and improve the experience of black, latinx, and native american people in the economics profession. *J. Econ. Perspectives* **34**, 193–219 (2020).
3. A Singhal, , et al., Modern information retrieval: A brief overview. *IEEE Data Eng. Bull.* **24**, 35–43 (2001).
4. Y Chen, R Farzan, R Kraut, I YeckehZaare, AF Zhang, Motivating experts to contribute to digital public goods: A personalized field experiment on wikipedia. *Manag. Sci.* **70**, 3264–3280 (2024).
